# Supplementary figures and images for: Enterovirus A71 3AB protein facilitates immune evasion by blocking cGAS recognition of mtDNA (part 1 of 2)
Source: EMBO Rep. 2026 Apr 2;27(10):2675–702. doi: 10.1038/s44319-026-00756-x (PMC13219398; doi:10.1038/s44319-026-00756-x)

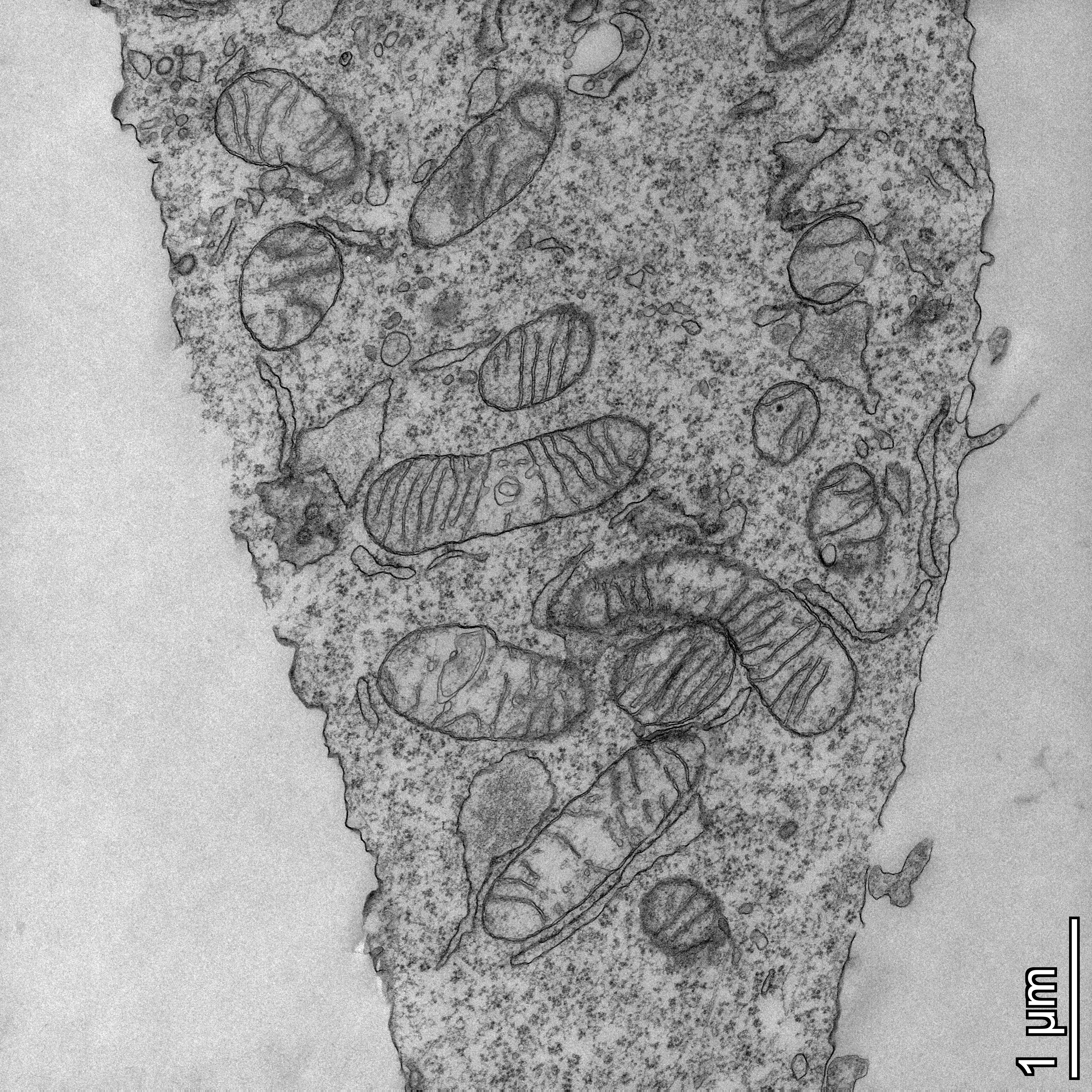

Supplement: Supplementary file 2 — Source data Fig. 1 [file 44319_2026_756_MOESM2_ESM.zip › Figure1/1A/Control.tif]

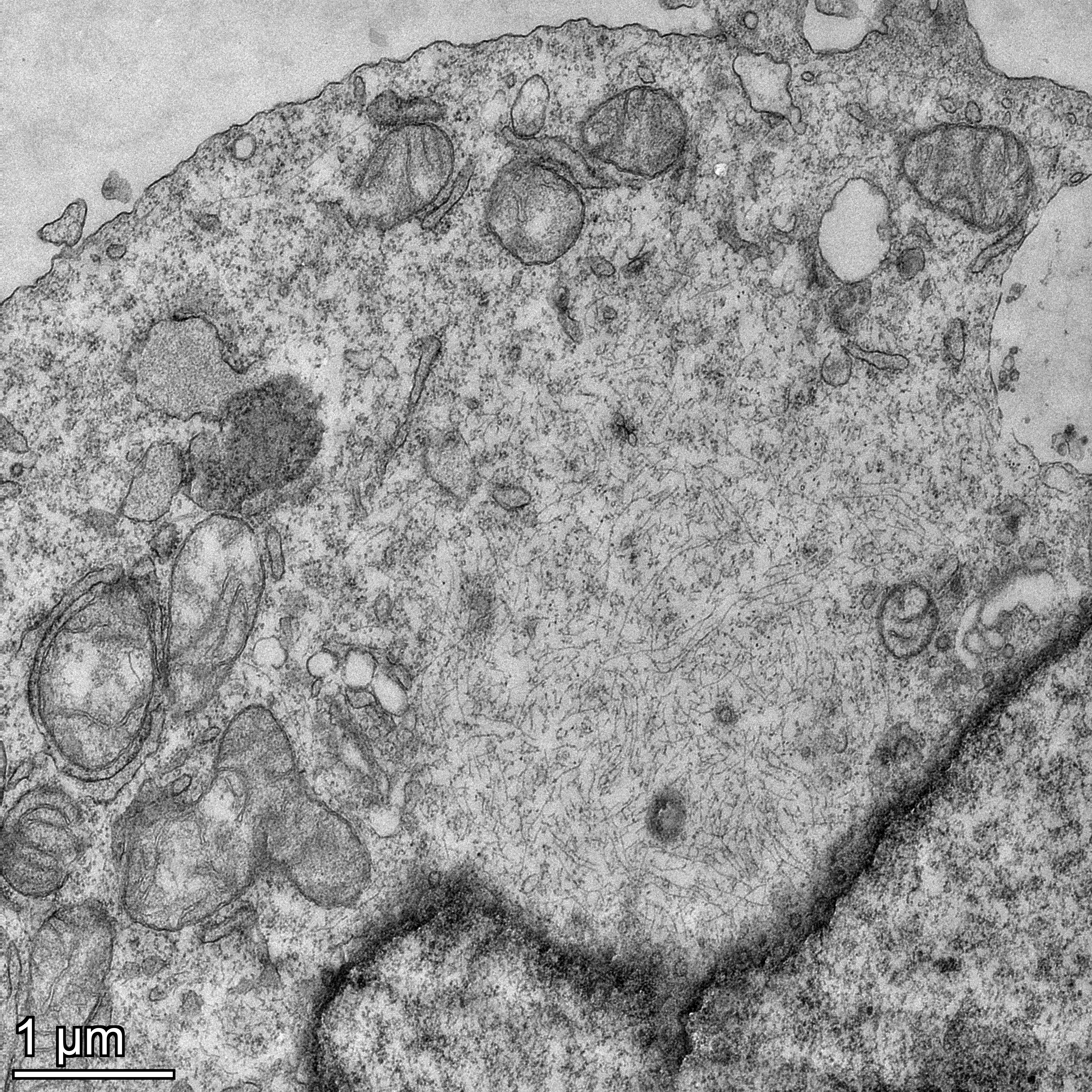

Supplement: Supplementary file 2 — Source data Fig. 1 [file 44319_2026_756_MOESM2_ESM.zip › Figure1/1A/EV-A71.tif]

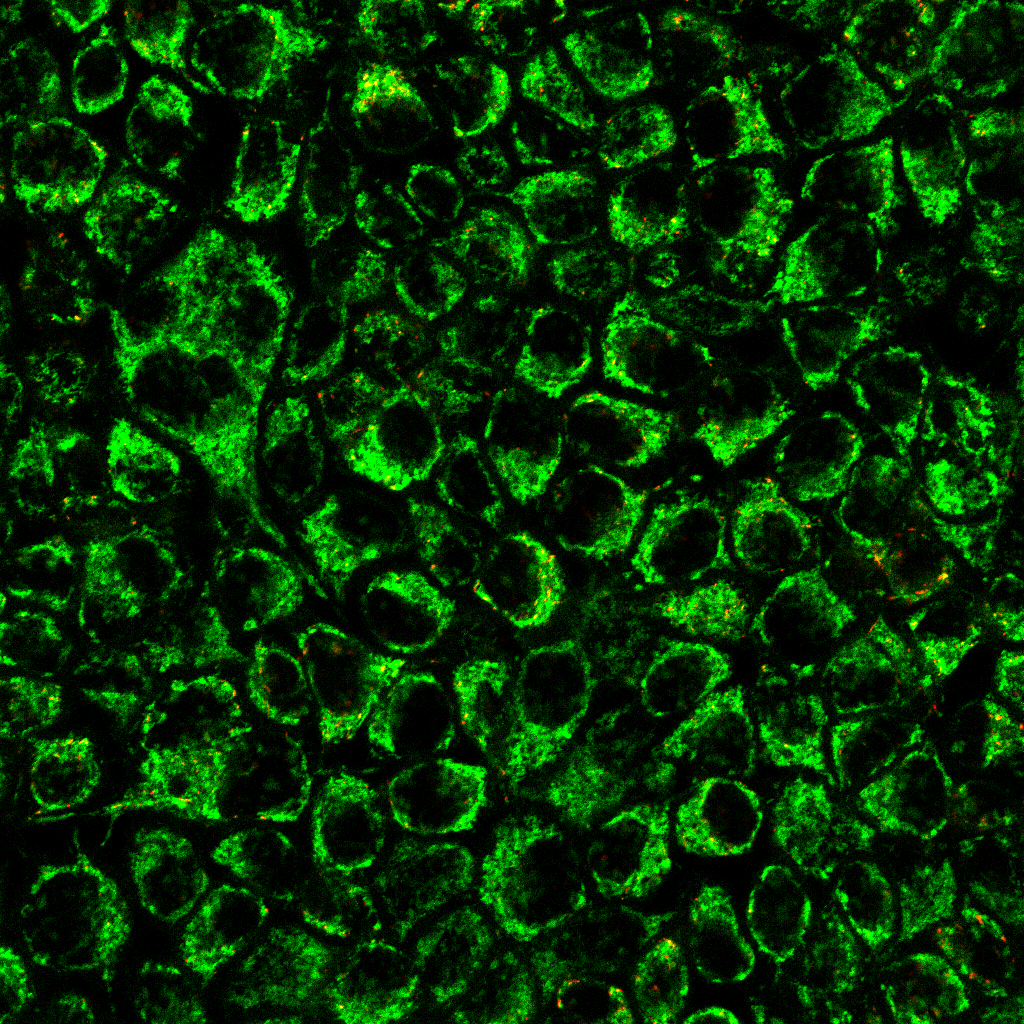

Supplement: Supplementary file 2 — Source data Fig. 1 [file 44319_2026_756_MOESM2_ESM.zip › Figure1/1B/HeLa-CCCP.tif]

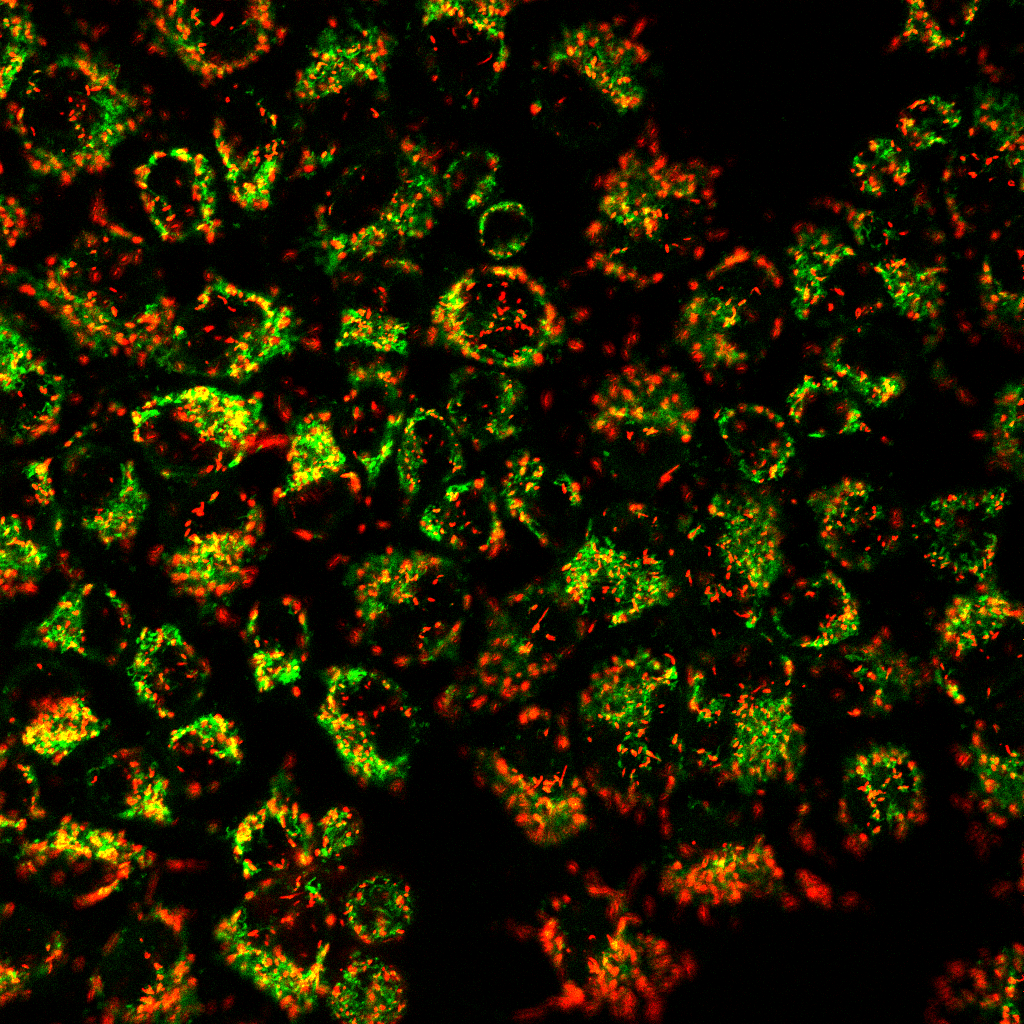

Supplement: Supplementary file 2 — Source data Fig. 1 [file 44319_2026_756_MOESM2_ESM.zip › Figure1/1B/HeLa-Control.tif]

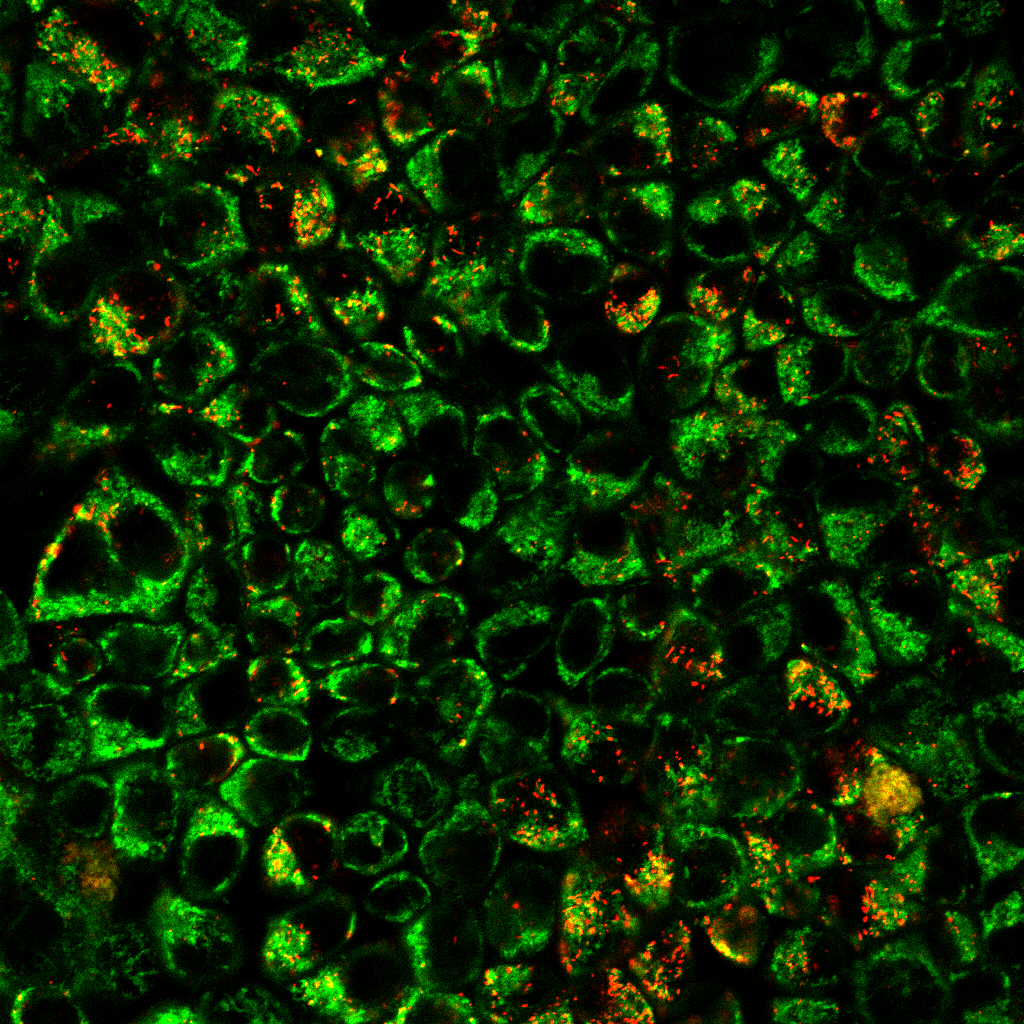

Supplement: Supplementary file 2 — Source data Fig. 1 [file 44319_2026_756_MOESM2_ESM.zip › Figure1/1B/HeLa-EV-A71.tif]

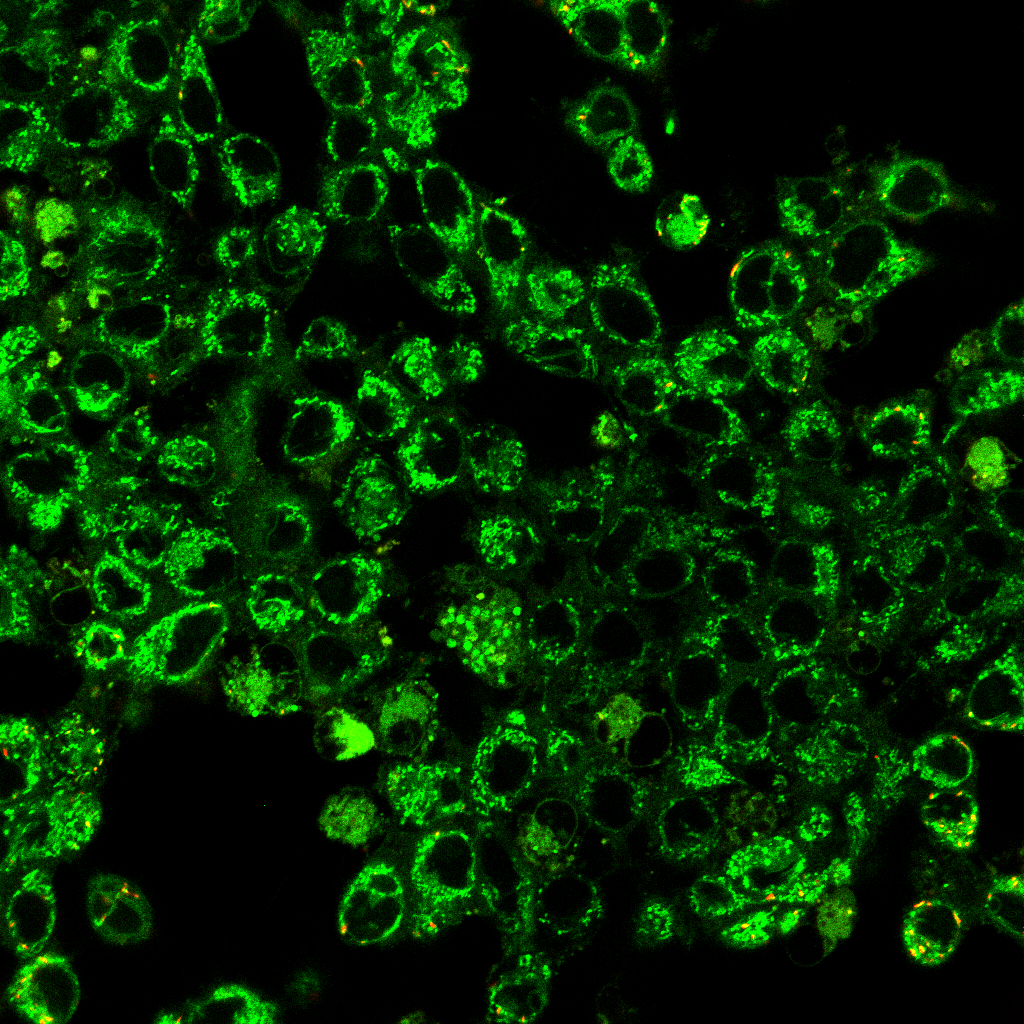

Supplement: Supplementary file 2 — Source data Fig. 1 [file 44319_2026_756_MOESM2_ESM.zip › Figure1/1B/RD-CCCP.tif]

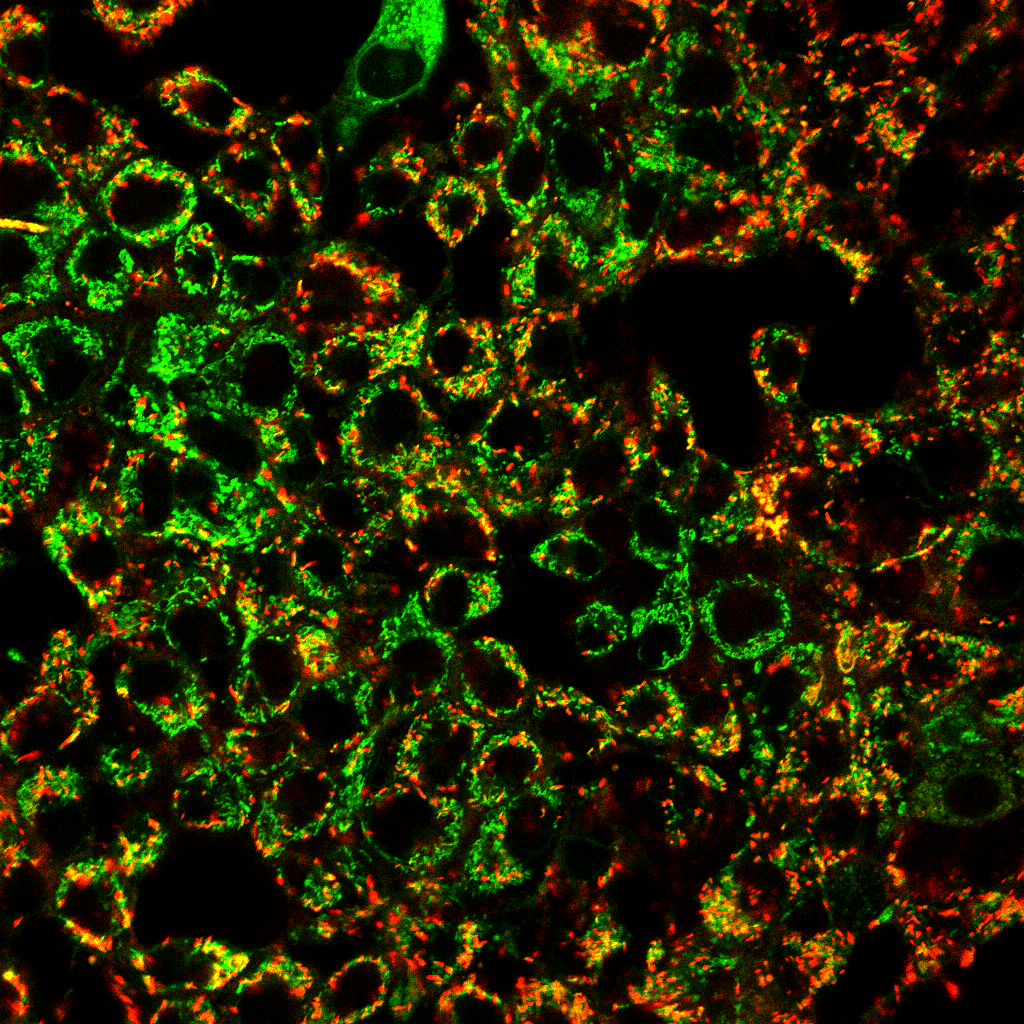

Supplement: Supplementary file 2 — Source data Fig. 1 [file 44319_2026_756_MOESM2_ESM.zip › Figure1/1B/RD-Control.tif]

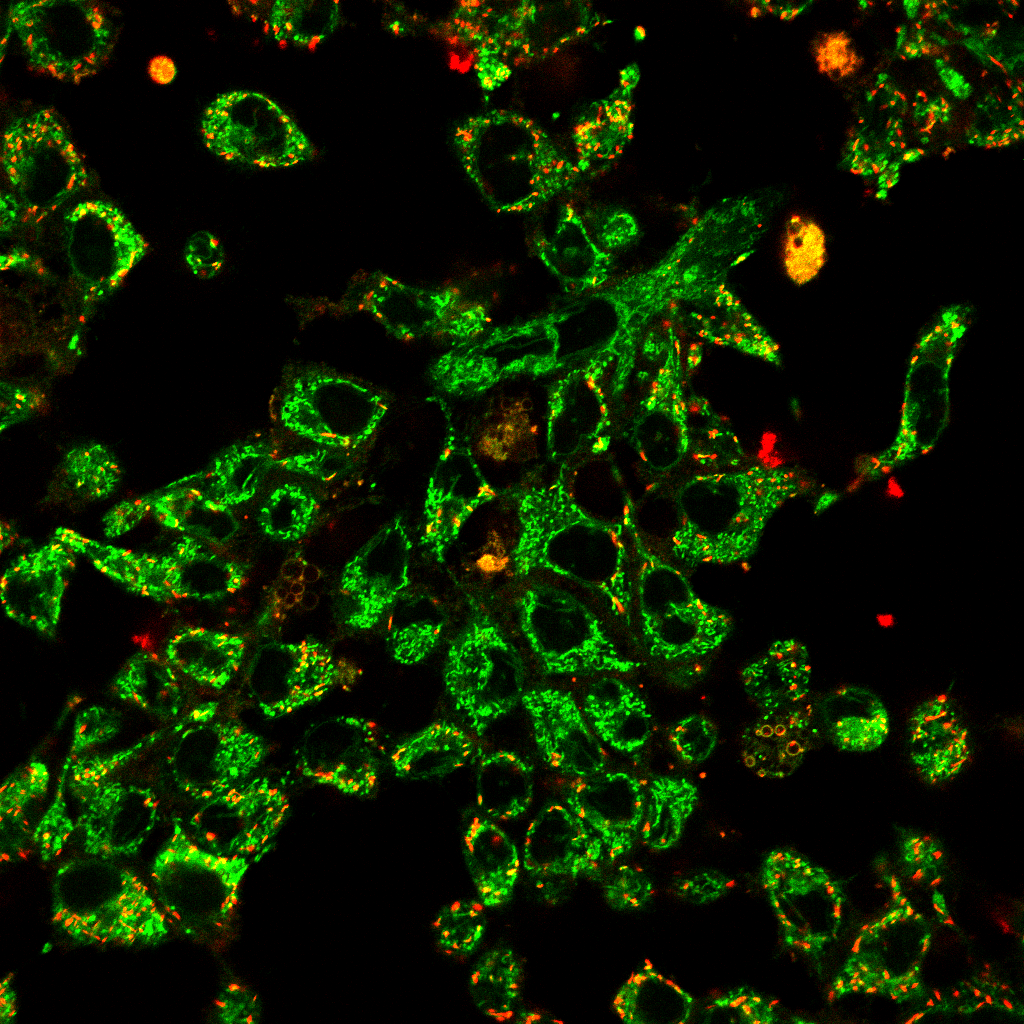

Supplement: Supplementary file 2 — Source data Fig. 1 [file 44319_2026_756_MOESM2_ESM.zip › Figure1/1B/RD-EV-A71.tif]

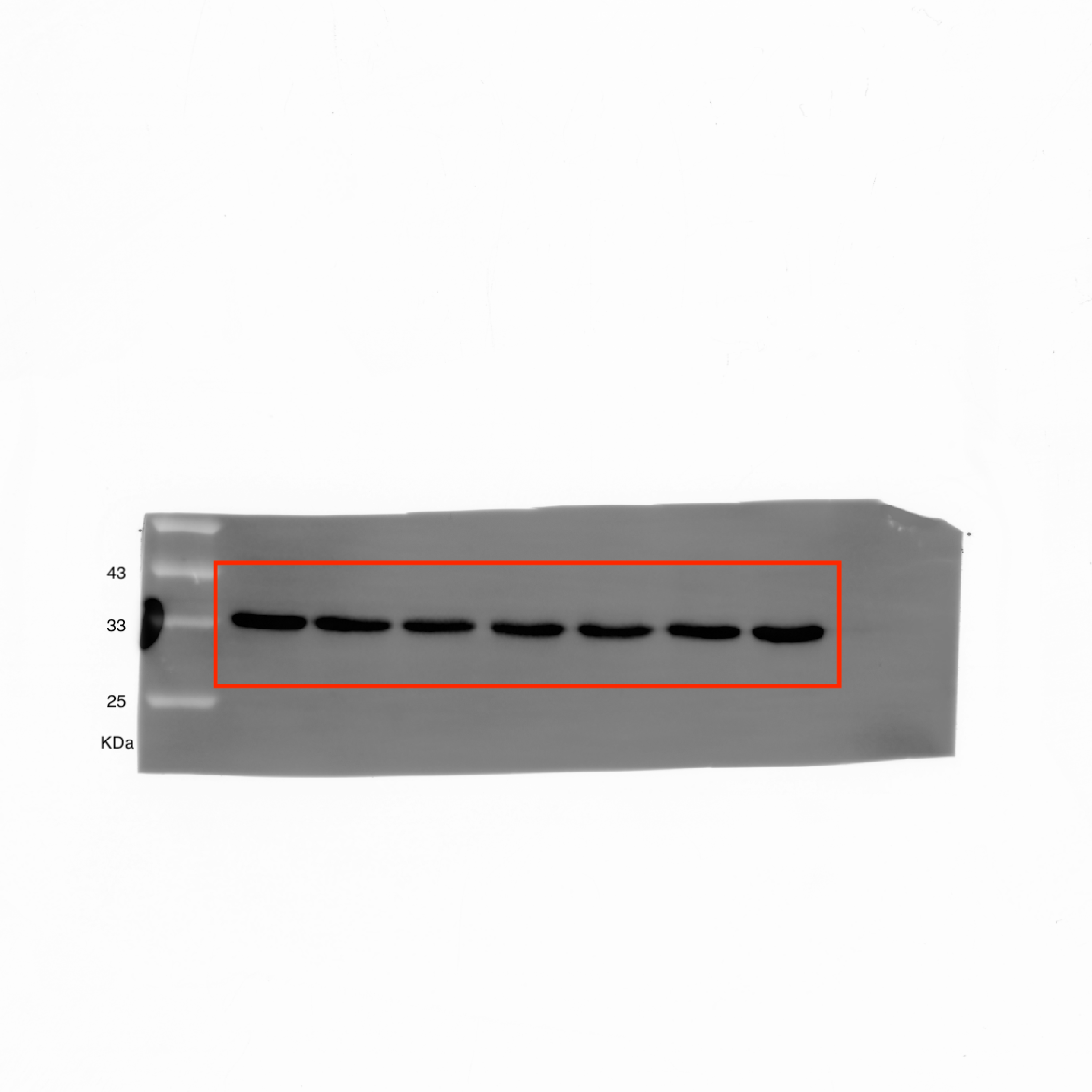

Supplement: Supplementary file 2 — Source data Fig. 1 [file 44319_2026_756_MOESM2_ESM.zip › Figure1/1D/western GAPDH.tif]

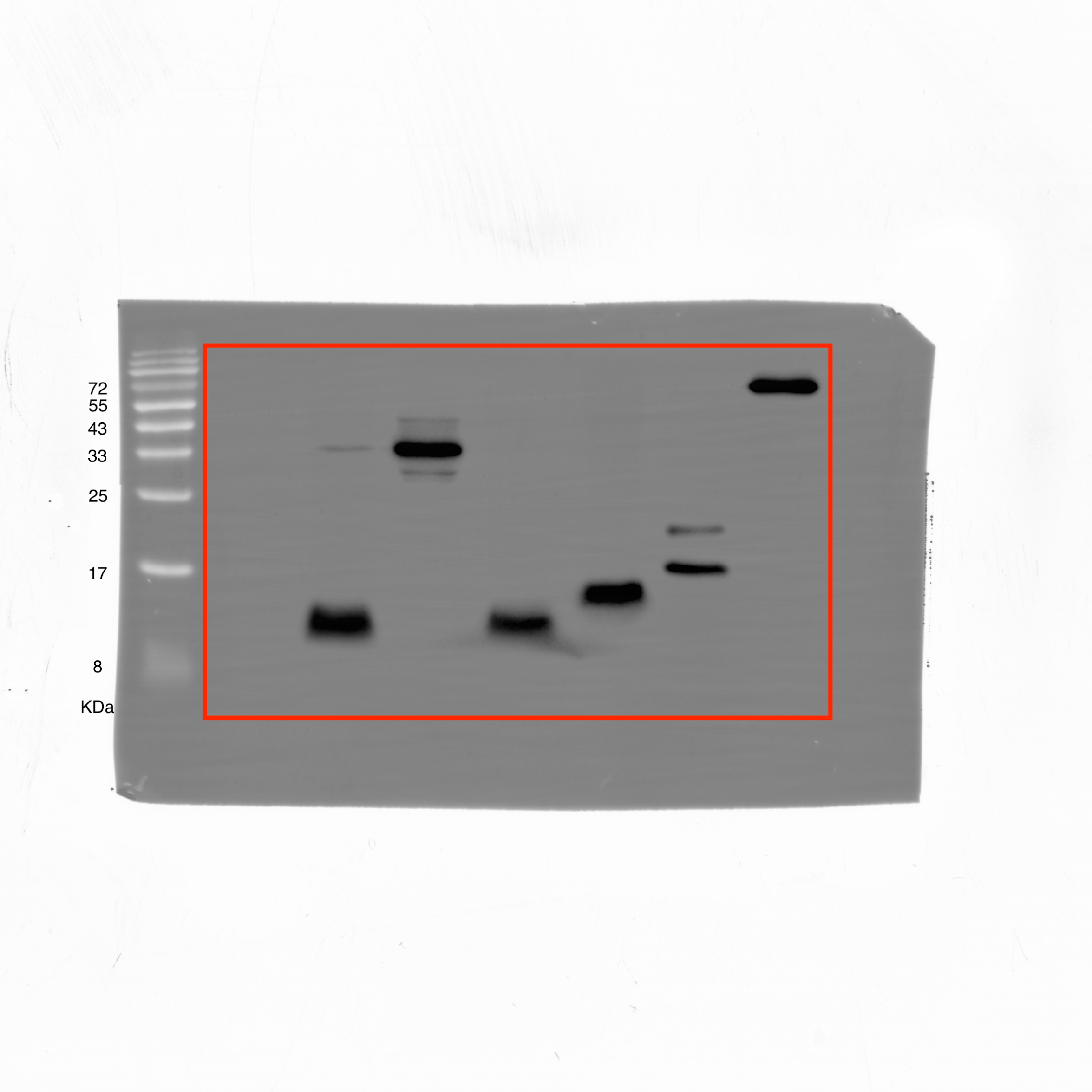

Supplement: Supplementary file 2 — Source data Fig. 1 [file 44319_2026_756_MOESM2_ESM.zip › Figure1/1D/western HA.tif]

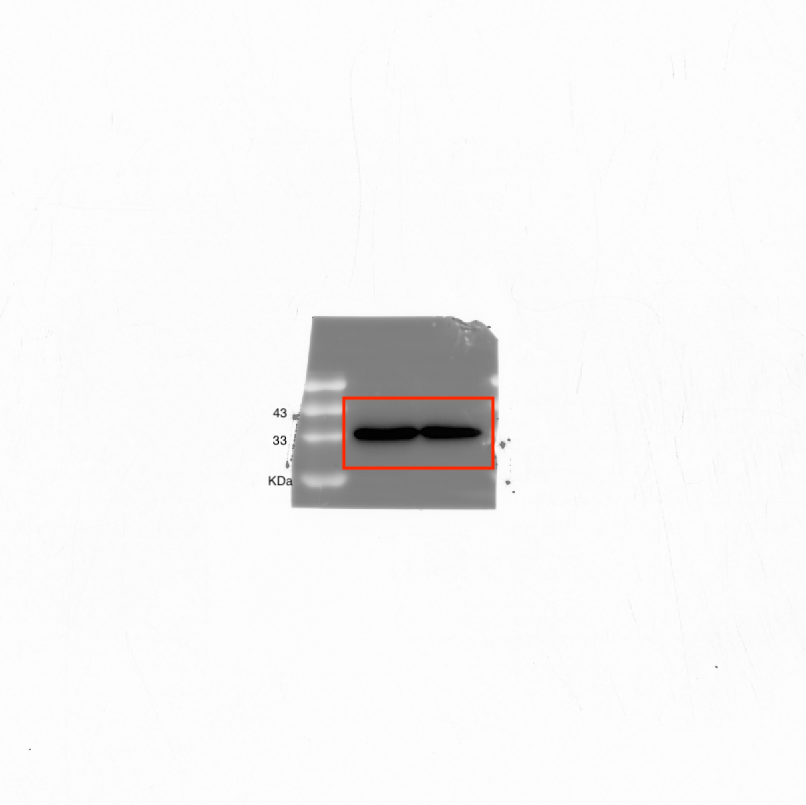

Supplement: Supplementary file 3 — Source data Fig. 2 [file 44319_2026_756_MOESM3_ESM.zip › Figure2/2F/western GAPDH.tif]

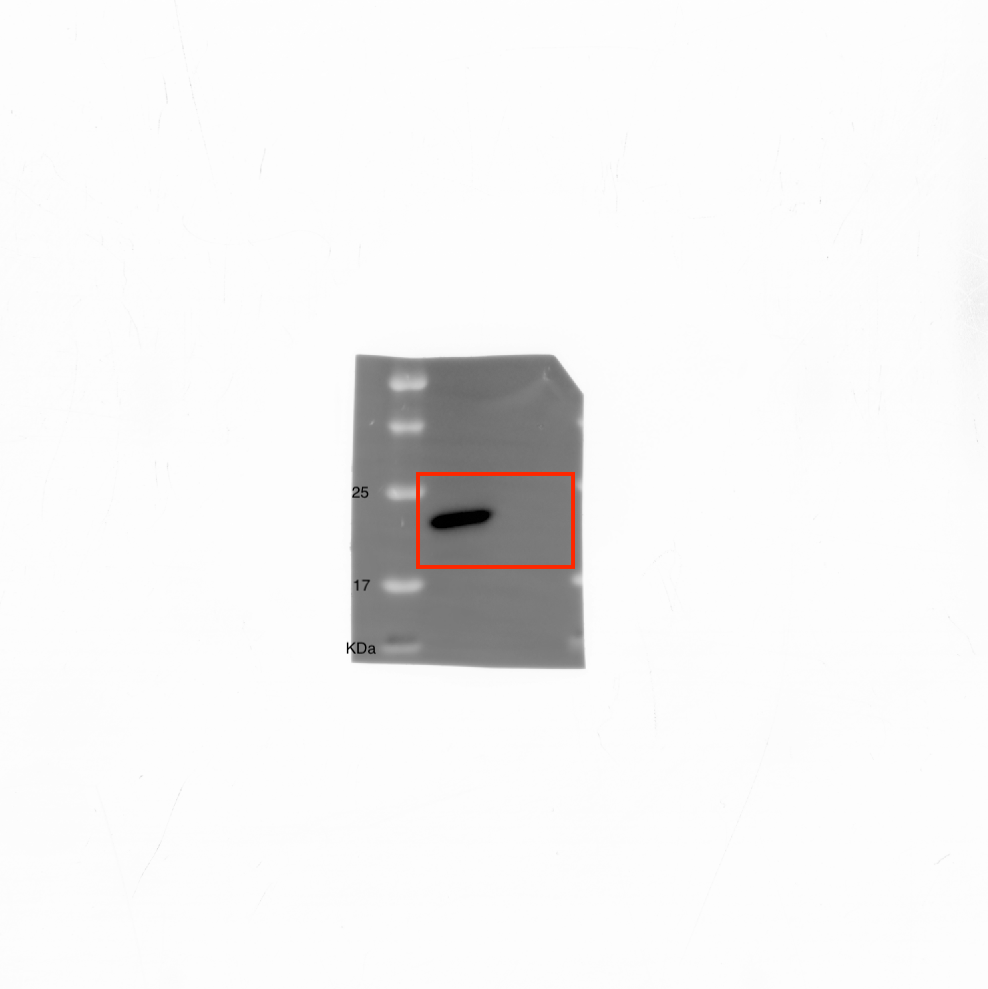

Supplement: Supplementary file 3 — Source data Fig. 2 [file 44319_2026_756_MOESM3_ESM.zip › Figure2/2F/western TFAM.tif]

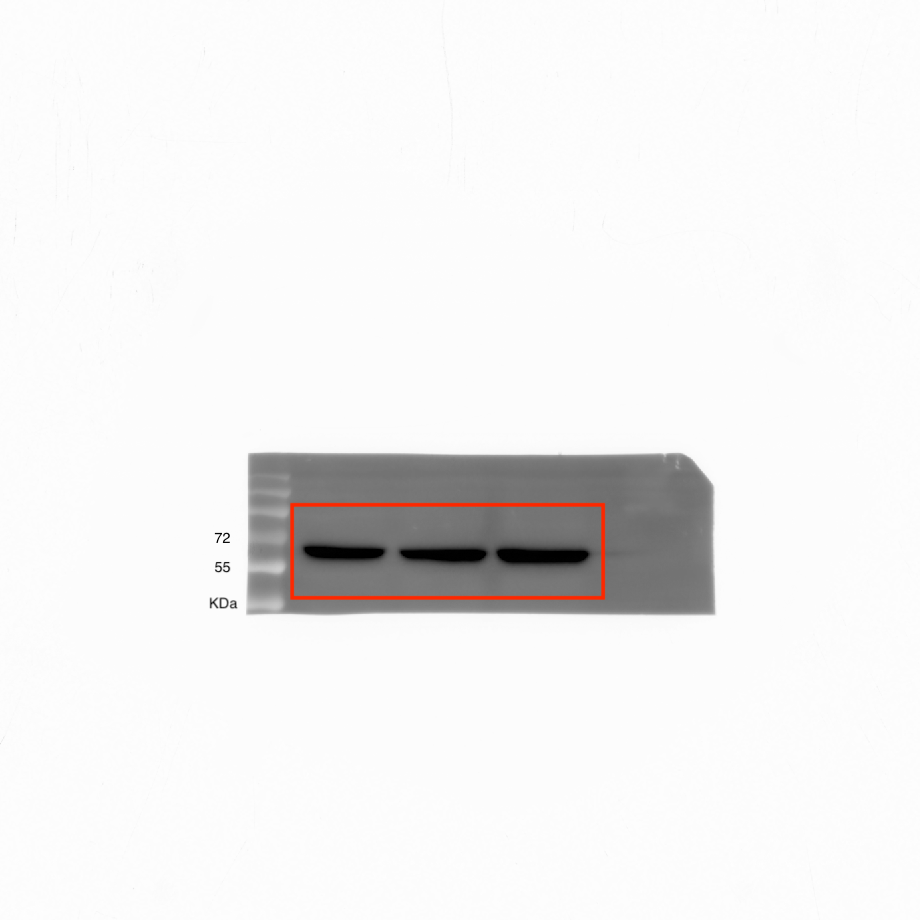

Supplement: Supplementary file 3 — Source data Fig. 2 [file 44319_2026_756_MOESM3_ESM.zip › Figure2/2I/western cGAS.tif]

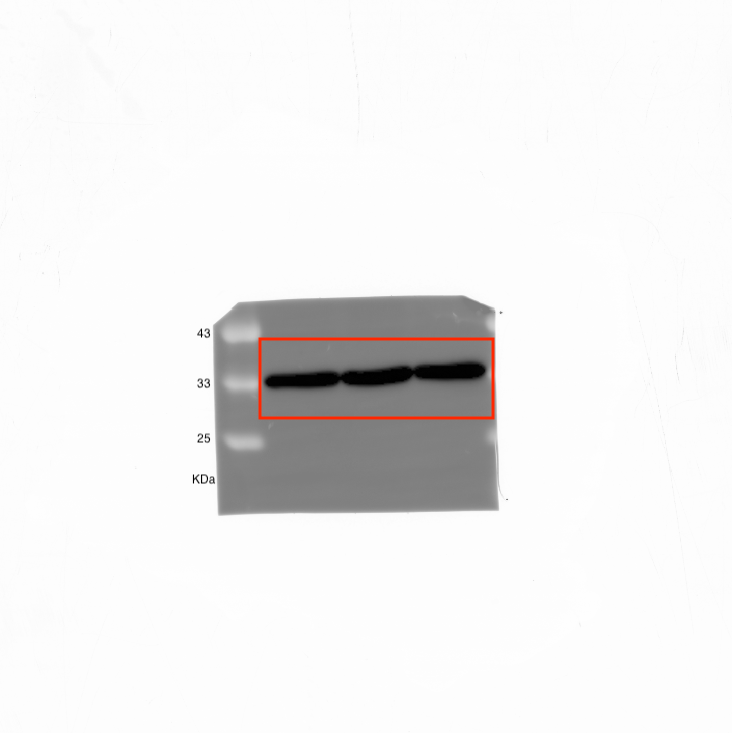

Supplement: Supplementary file 3 — Source data Fig. 2 [file 44319_2026_756_MOESM3_ESM.zip › Figure2/2I/western GAPDH.tif]

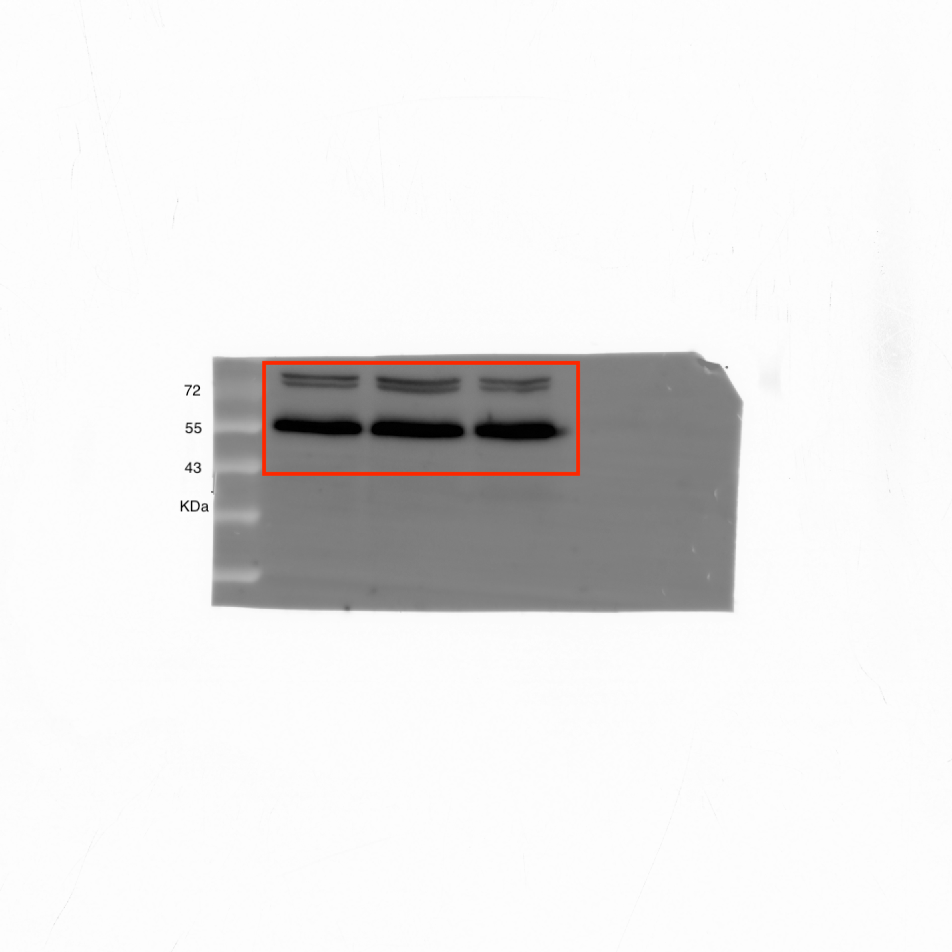

Supplement: Supplementary file 3 — Source data Fig. 2 [file 44319_2026_756_MOESM3_ESM.zip › Figure2/2I/western IRF3.tif]

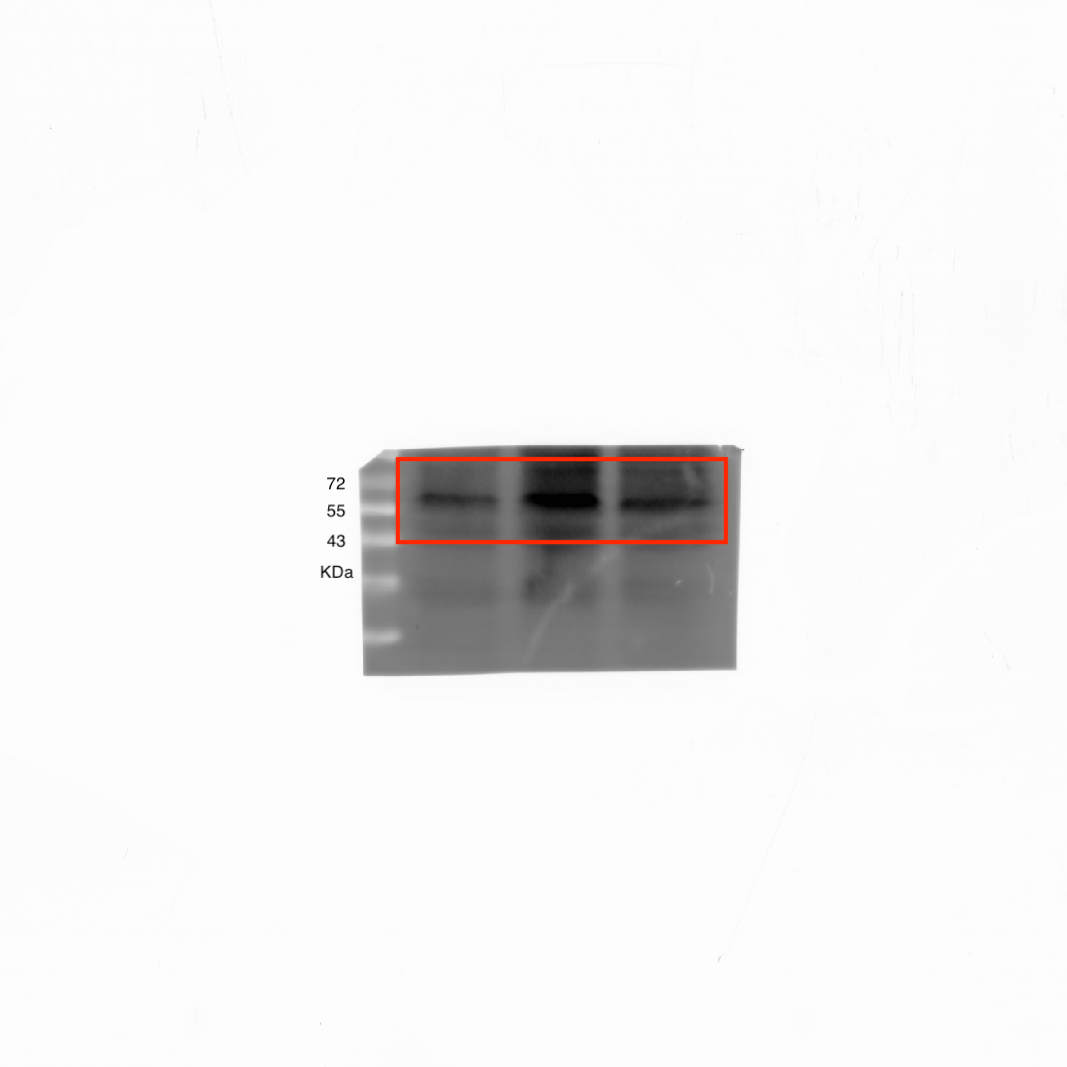

Supplement: Supplementary file 3 — Source data Fig. 2 [file 44319_2026_756_MOESM3_ESM.zip › Figure2/2I/western p-IRF3.tif]

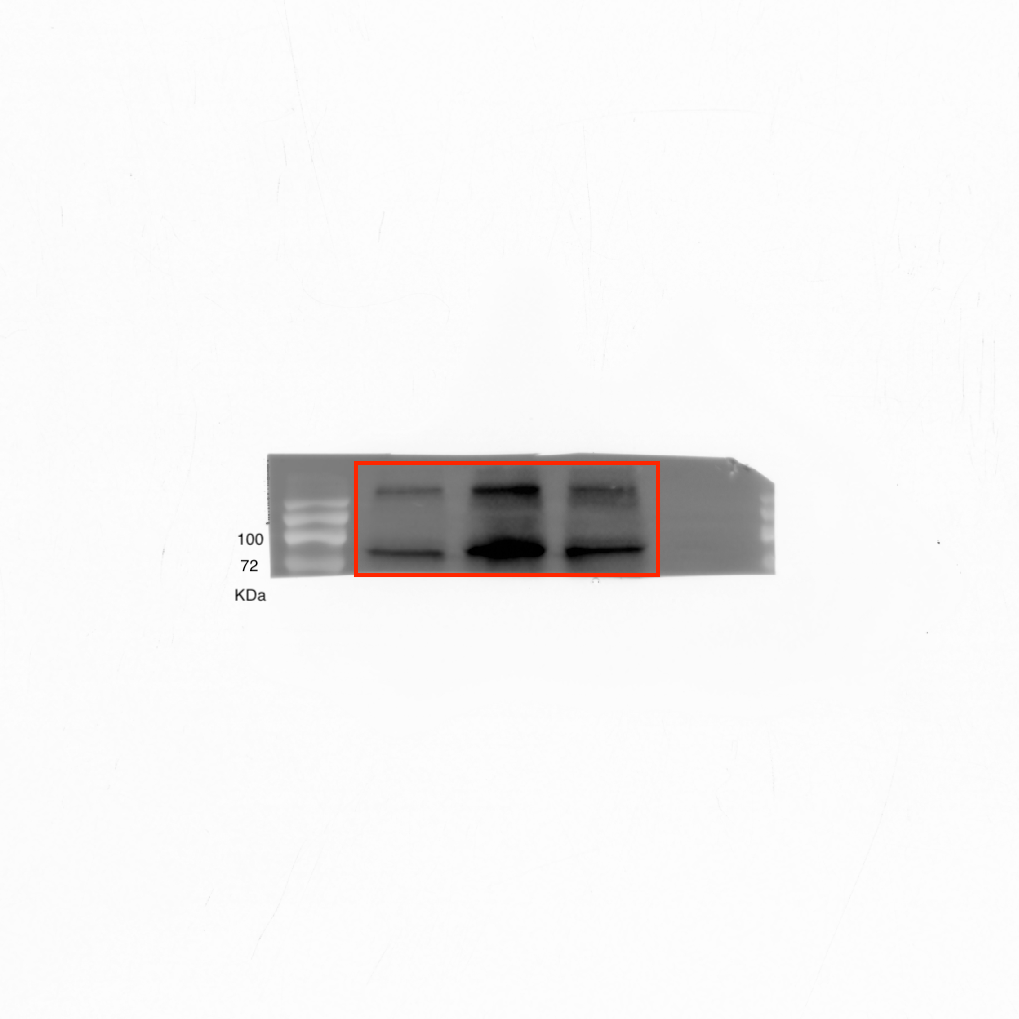

Supplement: Supplementary file 3 — Source data Fig. 2 [file 44319_2026_756_MOESM3_ESM.zip › Figure2/2I/western p-TBK1.tif]

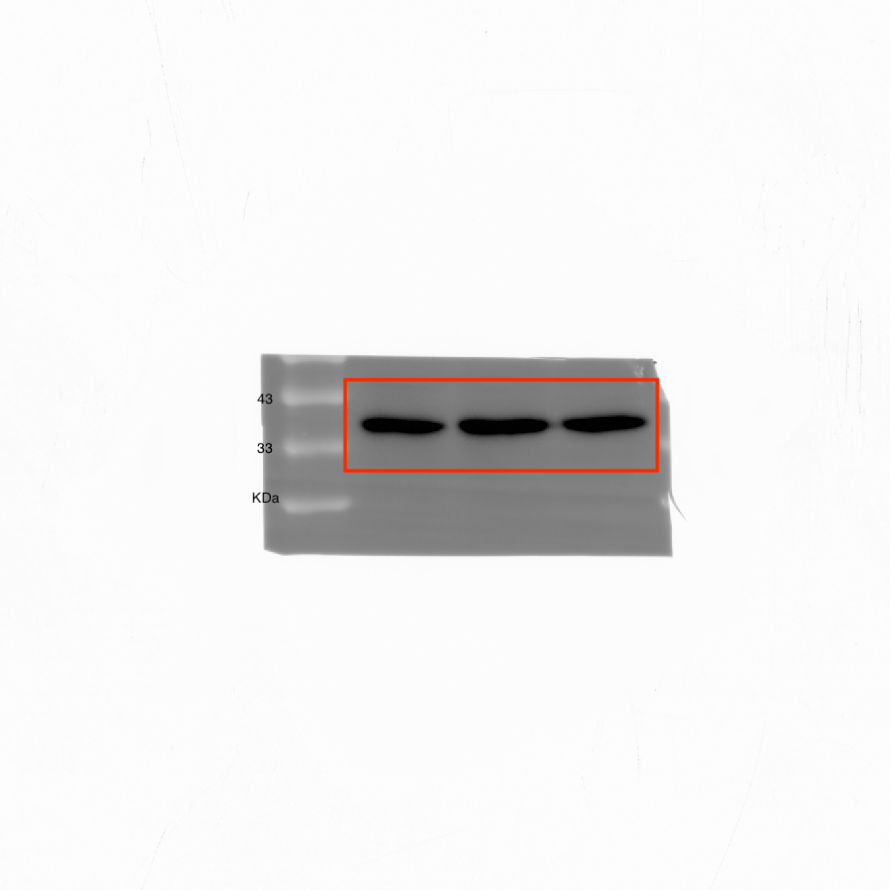

Supplement: Supplementary file 3 — Source data Fig. 2 [file 44319_2026_756_MOESM3_ESM.zip › Figure2/2I/western STING.tif]

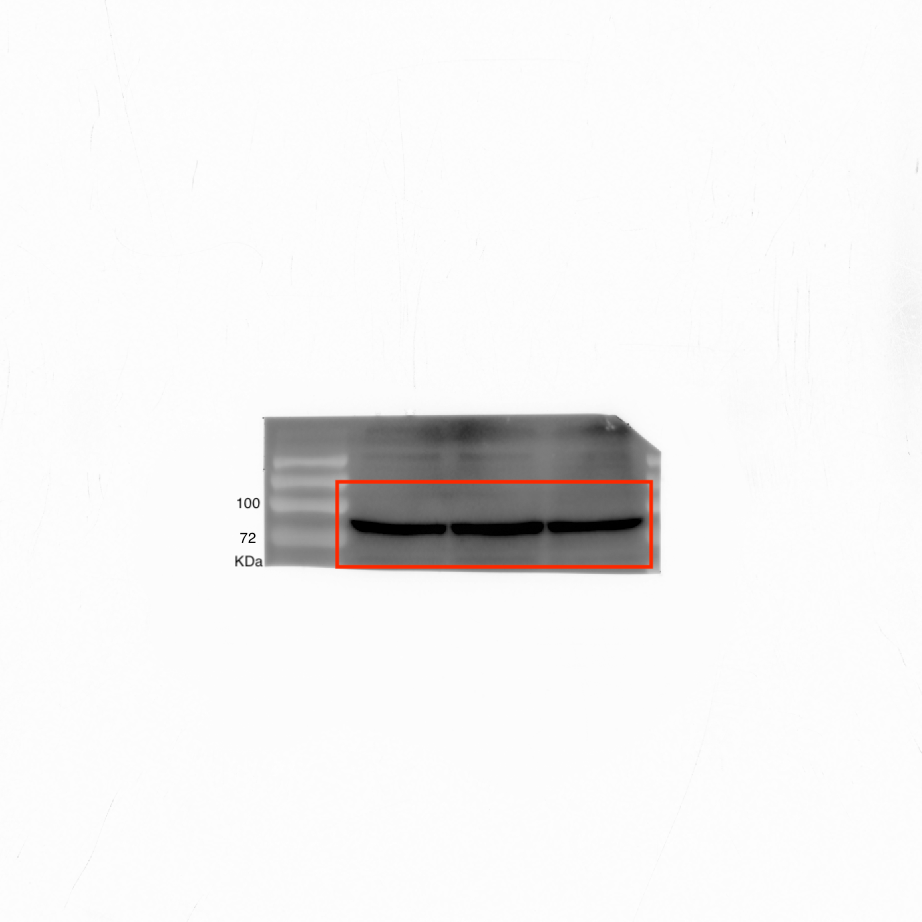

Supplement: Supplementary file 3 — Source data Fig. 2 [file 44319_2026_756_MOESM3_ESM.zip › Figure2/2I/western TBK1.tif]

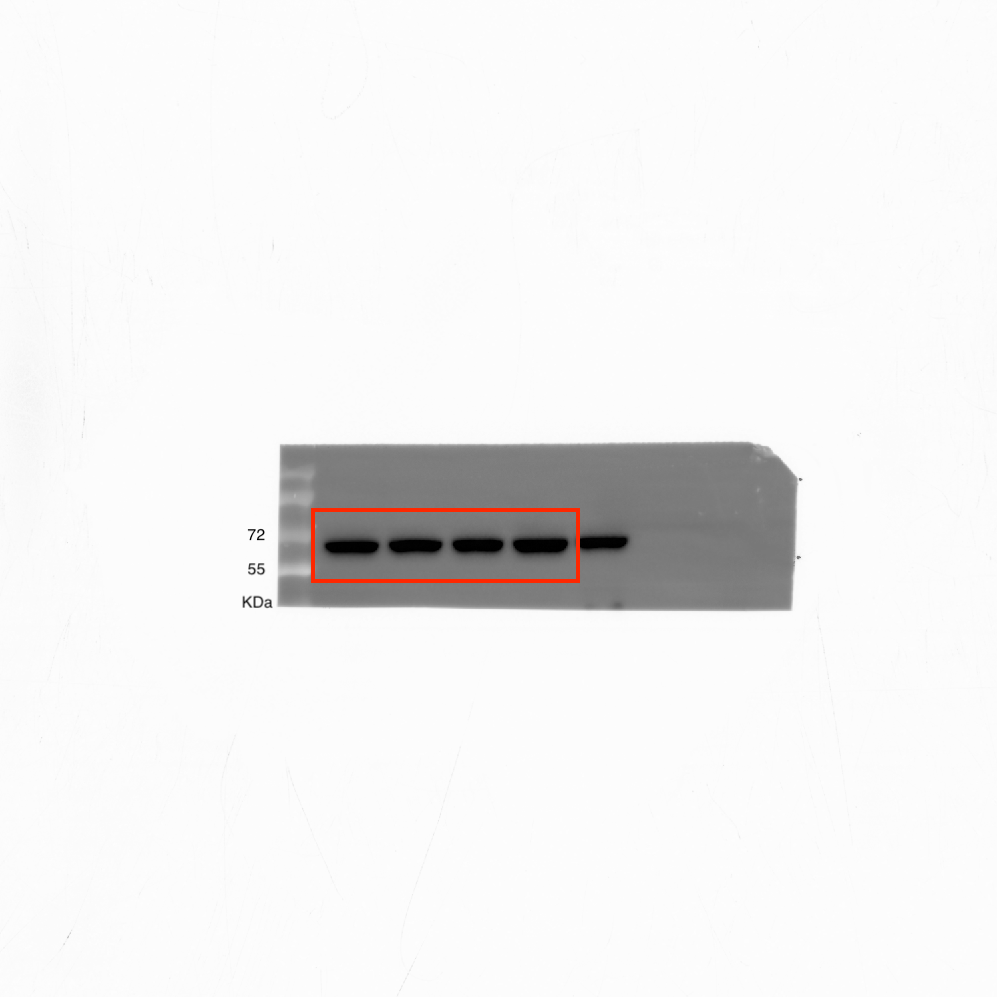

Supplement: Supplementary file 4 — Source data Fig. 3 [file 44319_2026_756_MOESM4_ESM.zip › Figure3/3A/western cGAS.tif]

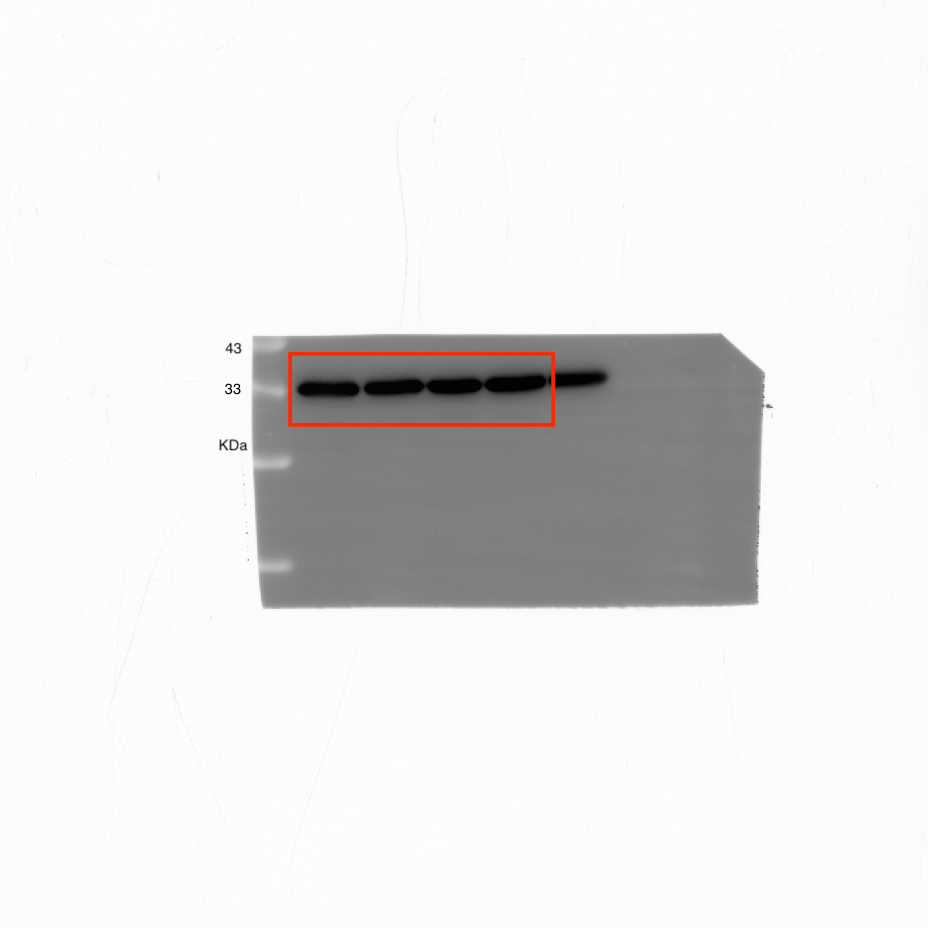

Supplement: Supplementary file 4 — Source data Fig. 3 [file 44319_2026_756_MOESM4_ESM.zip › Figure3/3A/western GAPDH.tif]

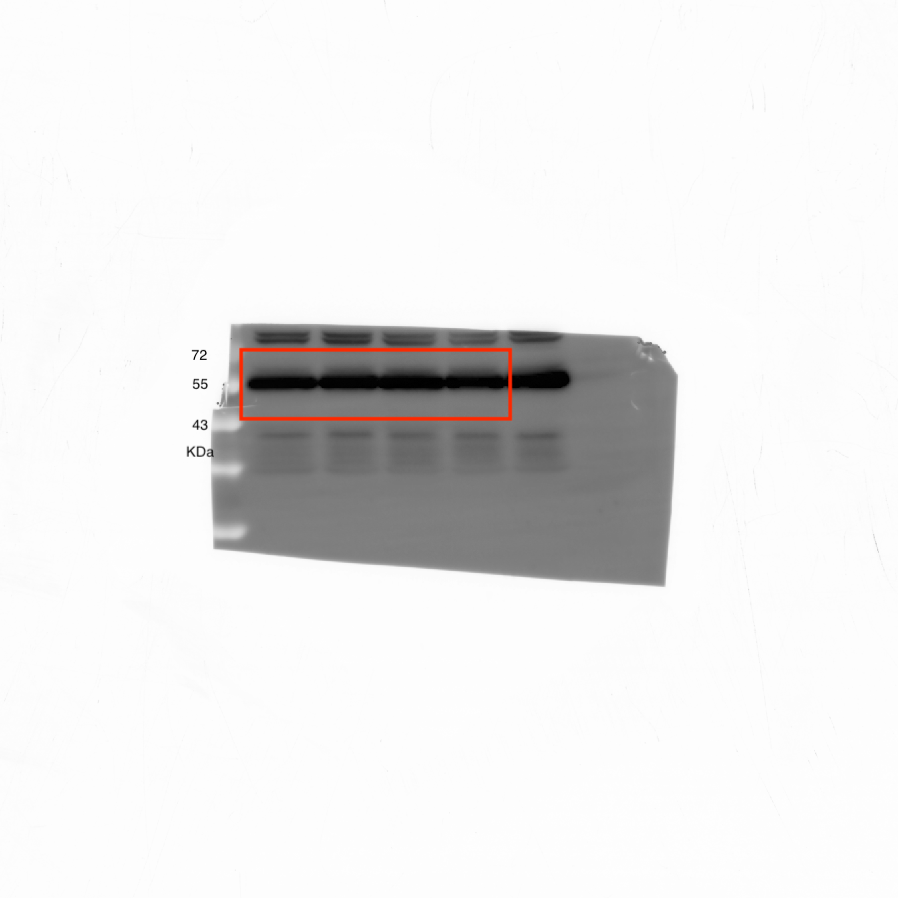

Supplement: Supplementary file 4 — Source data Fig. 3 [file 44319_2026_756_MOESM4_ESM.zip › Figure3/3A/western IRF3.tif]

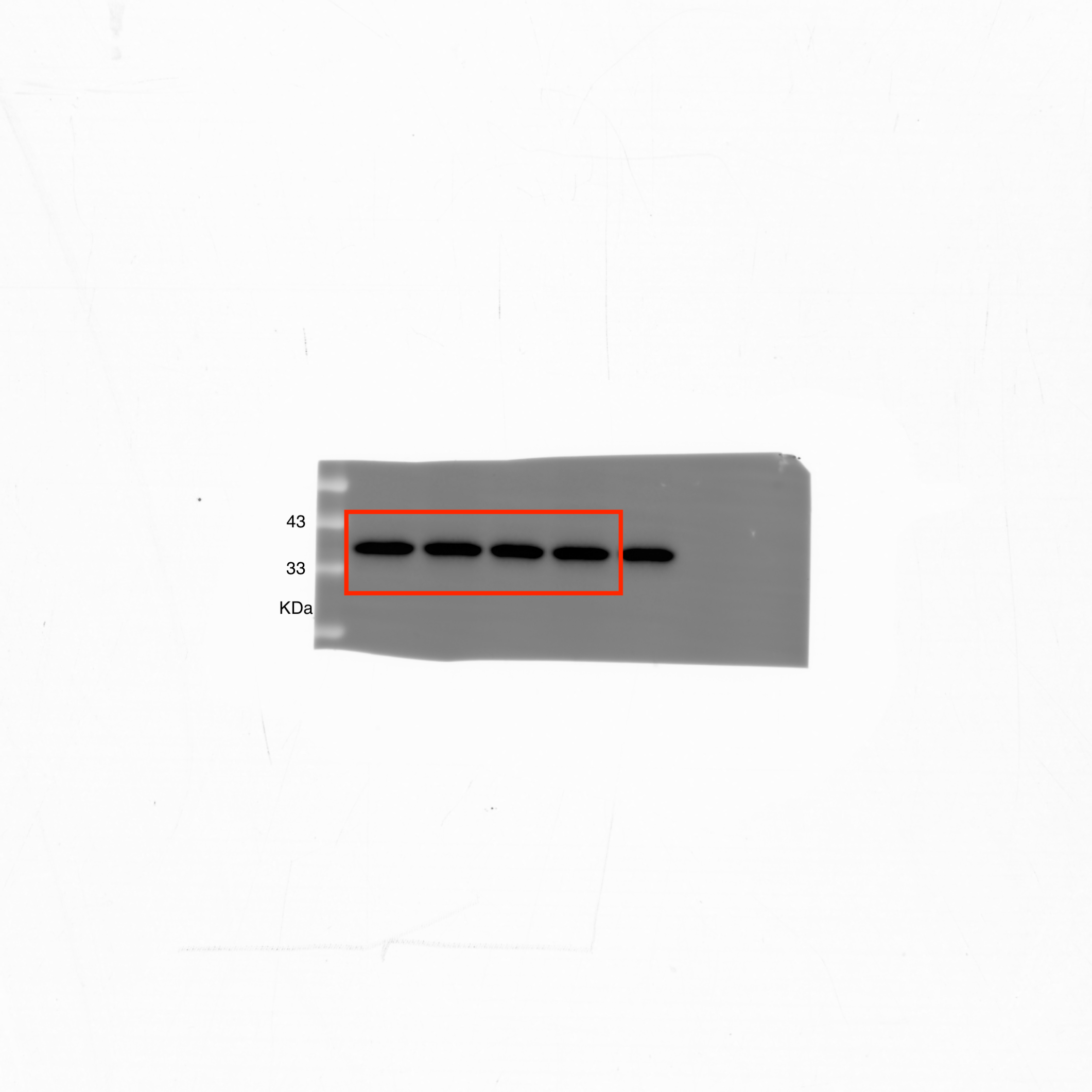

Supplement: Supplementary file 4 — Source data Fig. 3 [file 44319_2026_756_MOESM4_ESM.zip › Figure3/3A/western STING.tif]

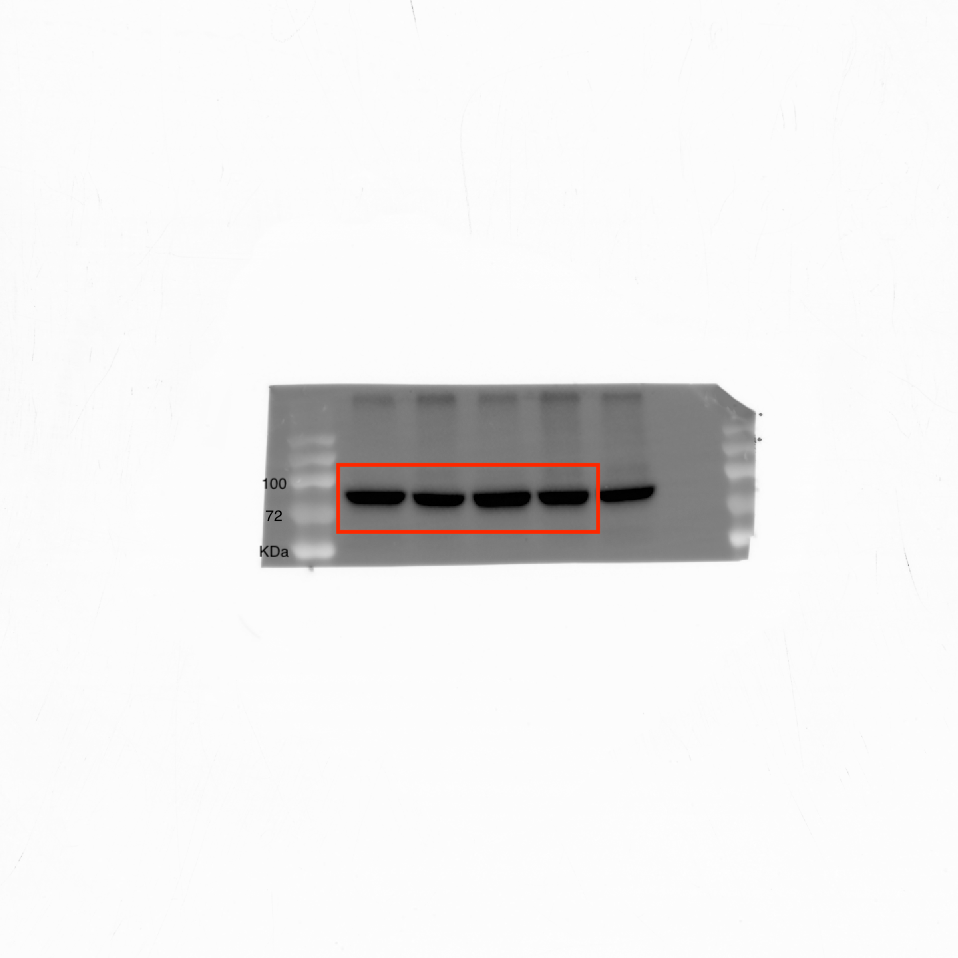

Supplement: Supplementary file 4 — Source data Fig. 3 [file 44319_2026_756_MOESM4_ESM.zip › Figure3/3A/western TBK1.tif]

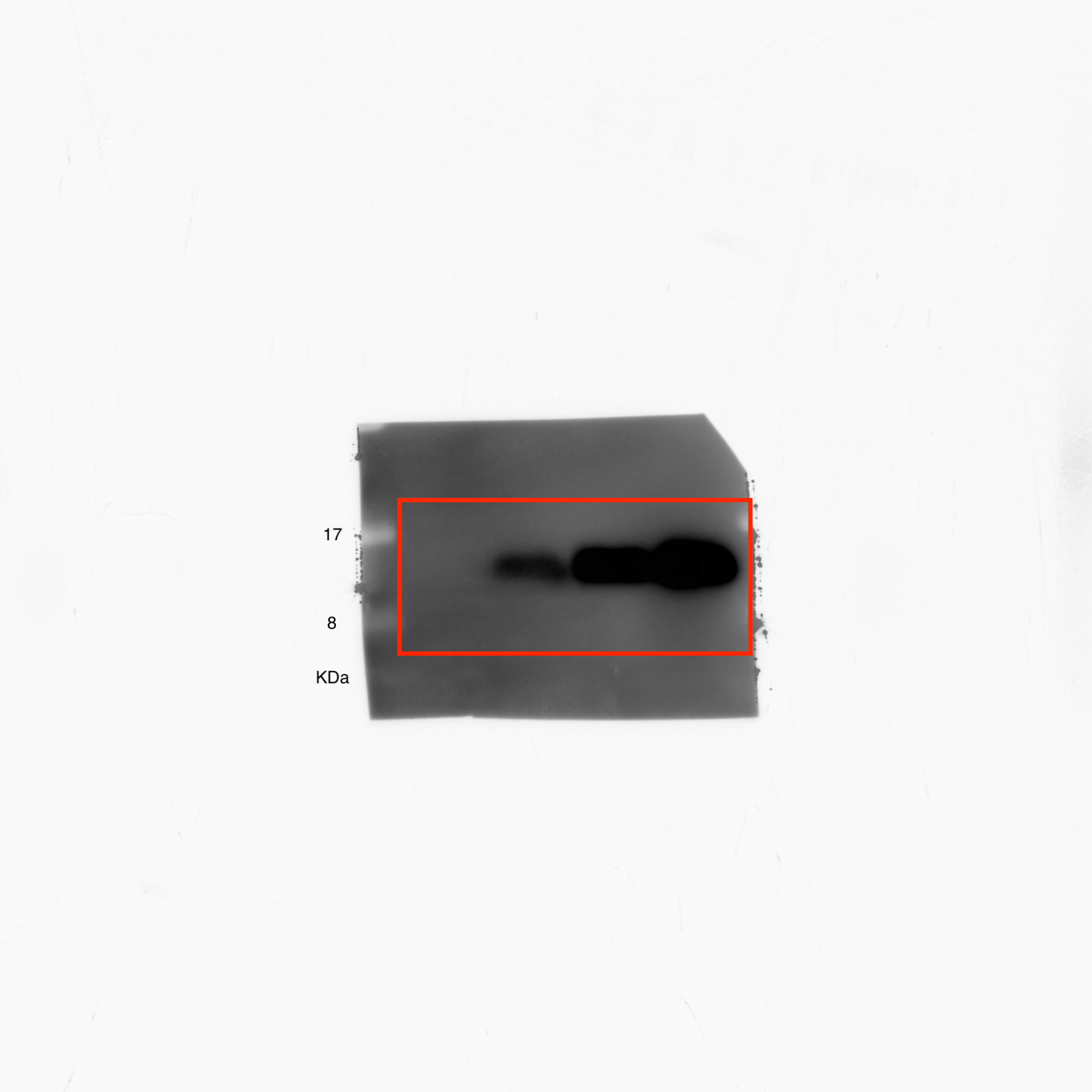

Supplement: Supplementary file 4 — Source data Fig. 3 [file 44319_2026_756_MOESM4_ESM.zip › Figure3/3L/western EVA-71 3AB.tif]

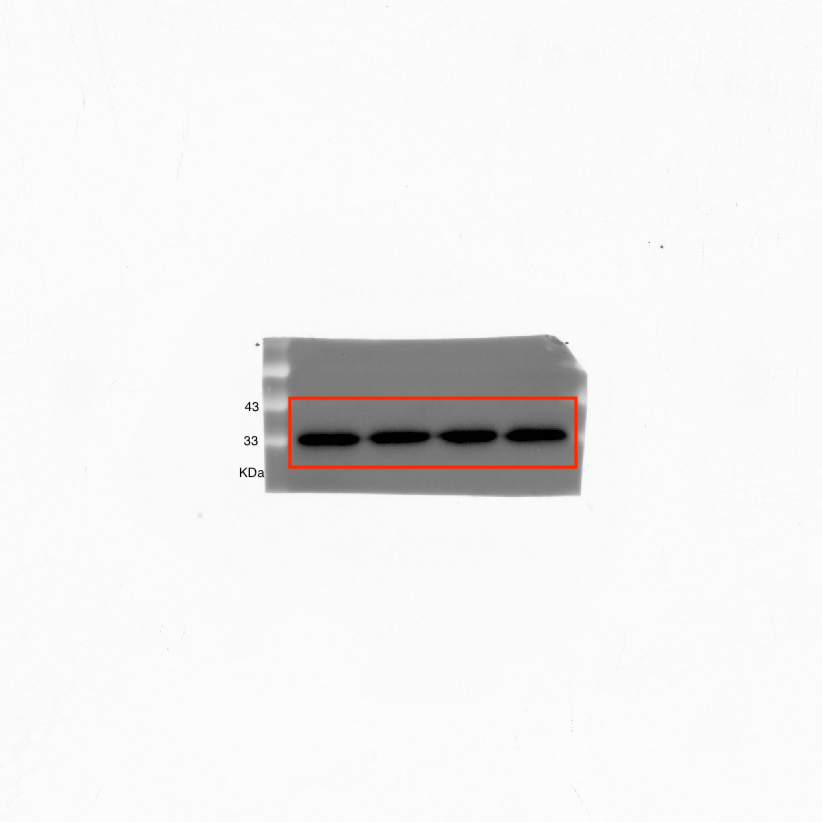

Supplement: Supplementary file 4 — Source data Fig. 3 [file 44319_2026_756_MOESM4_ESM.zip › Figure3/3L/western GAPDH.tif]

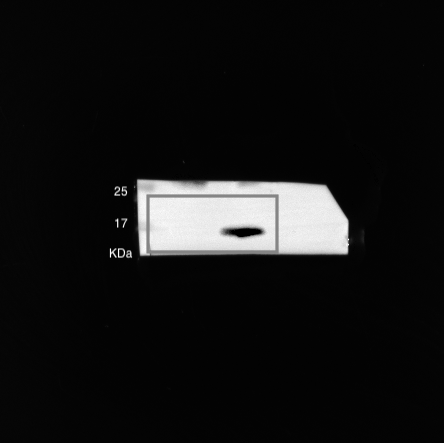

Supplement: Supplementary file 5 — Source data Fig. 4 [file 44319_2026_756_MOESM5_ESM.zip › Figure4/4B/western Input-HA.tif]

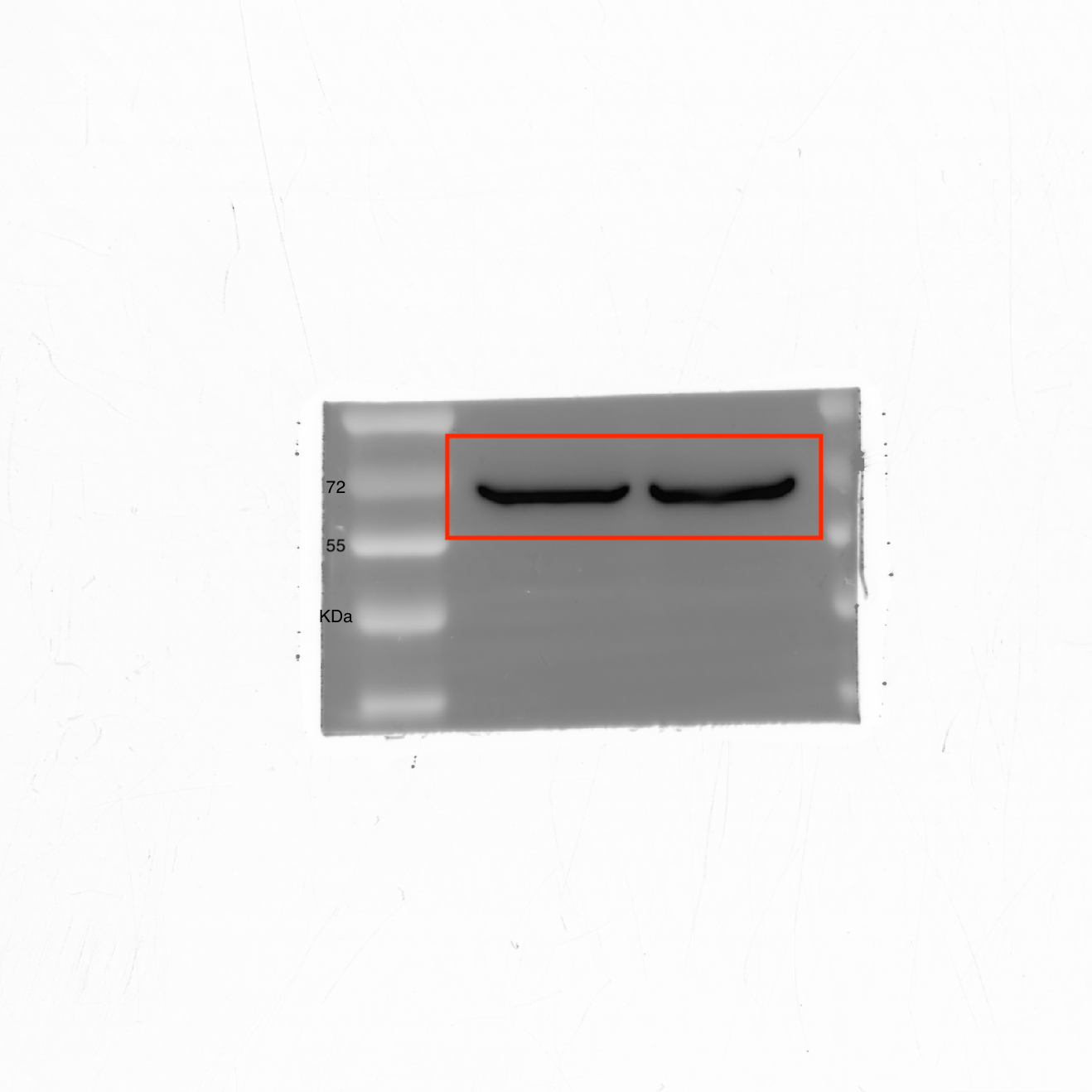

Supplement: Supplementary file 5 — Source data Fig. 4 [file 44319_2026_756_MOESM5_ESM.zip › Figure4/4B/western Input-Myc.tif]

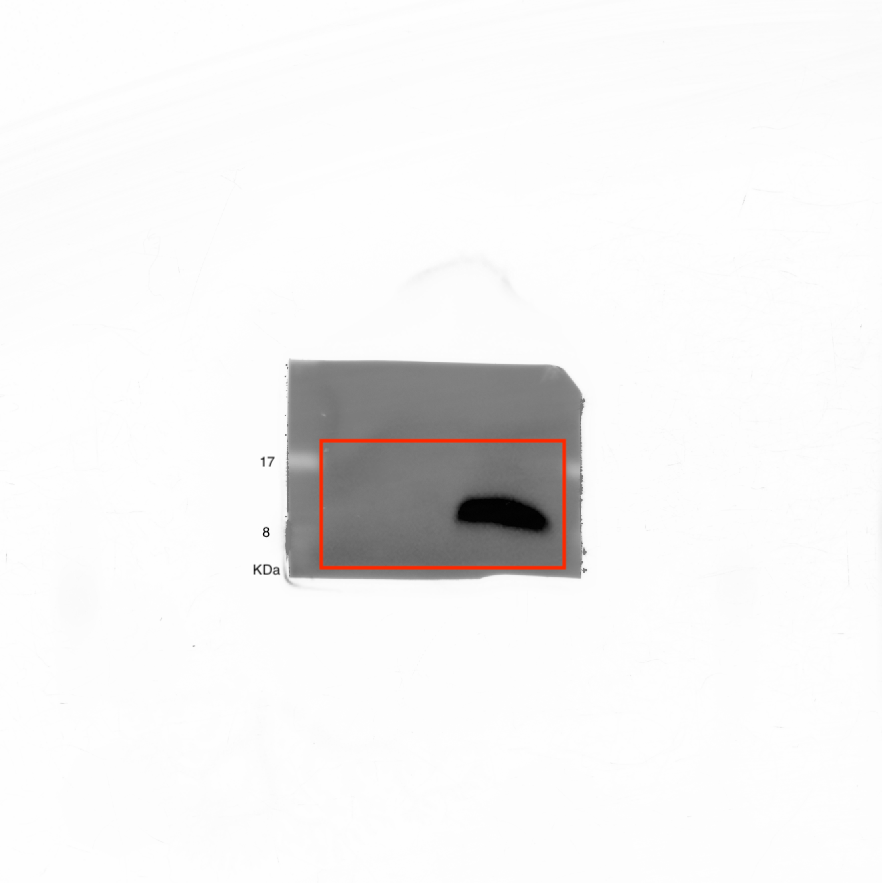

Supplement: Supplementary file 5 — Source data Fig. 4 [file 44319_2026_756_MOESM5_ESM.zip › Figure4/4B/western IP-HA.tif]

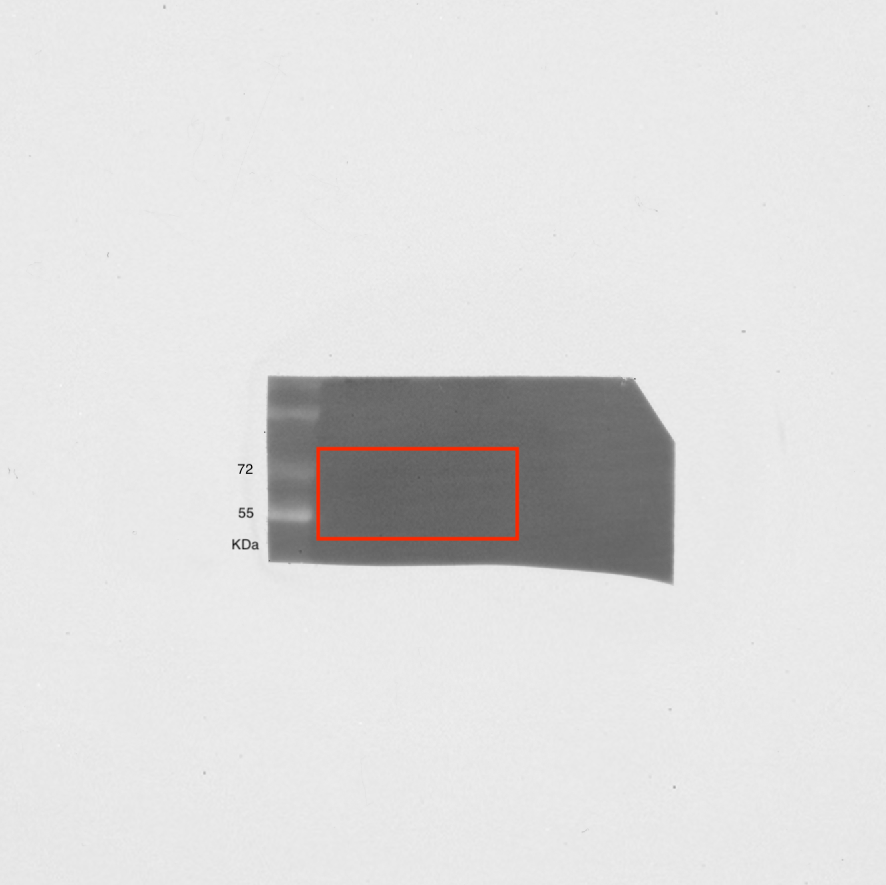

Supplement: Supplementary file 5 — Source data Fig. 4 [file 44319_2026_756_MOESM5_ESM.zip › Figure4/4B/western IP-Myc.tif]

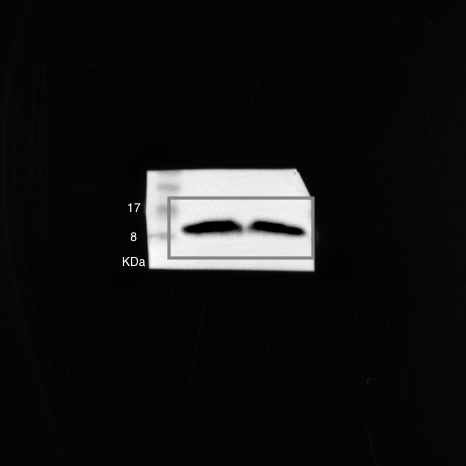

Supplement: Supplementary file 5 — Source data Fig. 4 [file 44319_2026_756_MOESM5_ESM.zip › Figure4/4C/western Input-HA.tif]

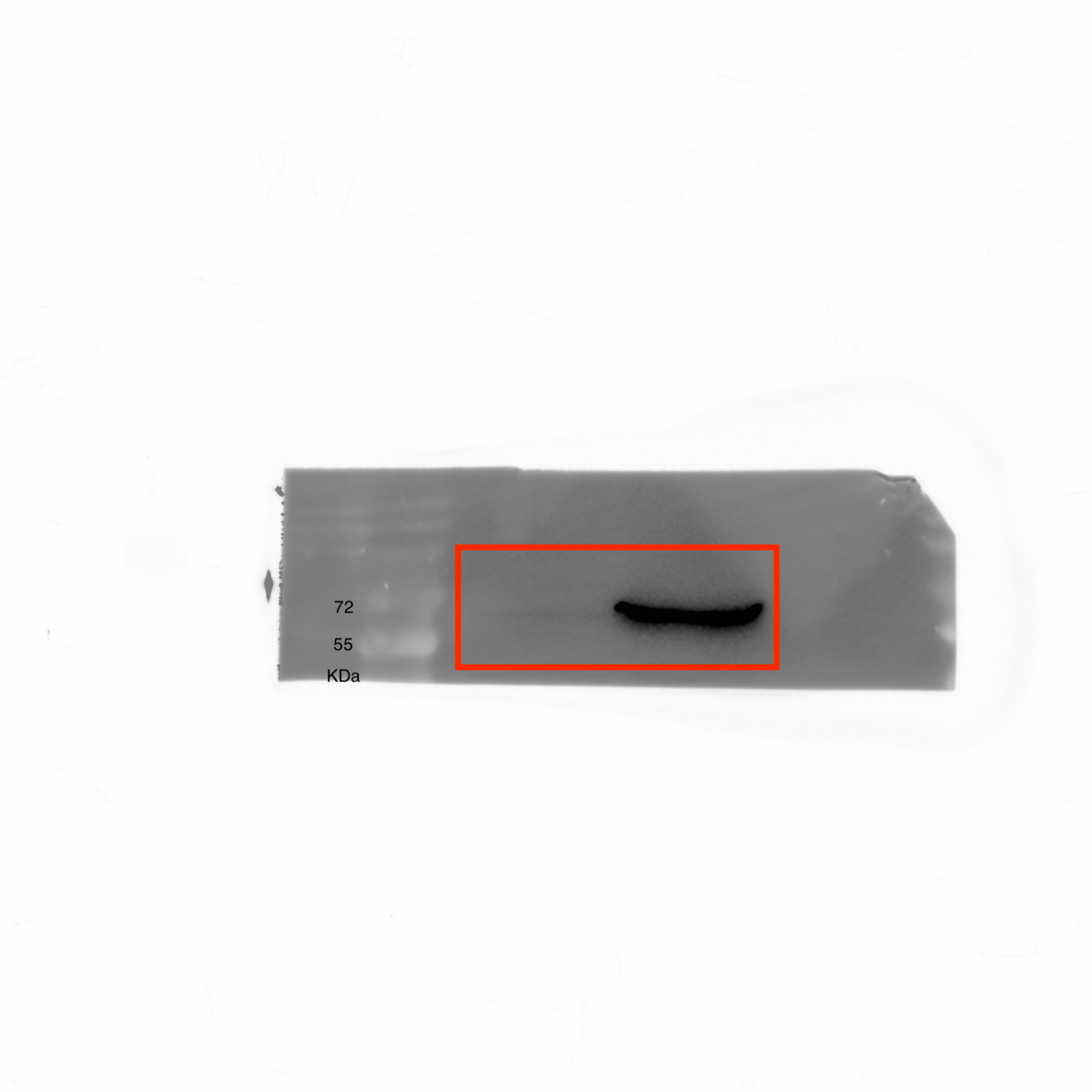

Supplement: Supplementary file 5 — Source data Fig. 4 [file 44319_2026_756_MOESM5_ESM.zip › Figure4/4C/western Input-Myc.tif]

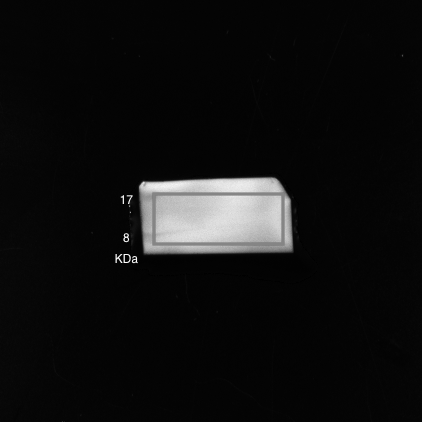

Supplement: Supplementary file 5 — Source data Fig. 4 [file 44319_2026_756_MOESM5_ESM.zip › Figure4/4C/western IP-HA.tif]

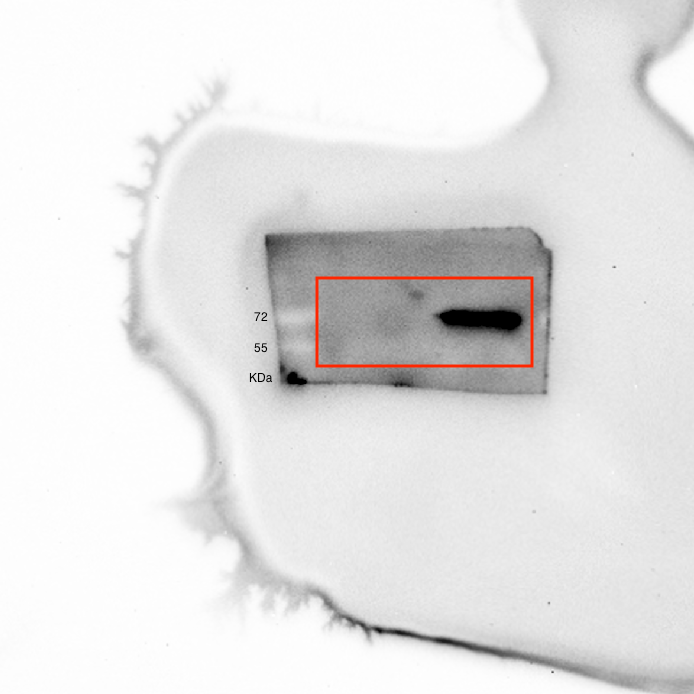

Supplement: Supplementary file 5 — Source data Fig. 4 [file 44319_2026_756_MOESM5_ESM.zip › Figure4/4C/western IP-Myc.tif]

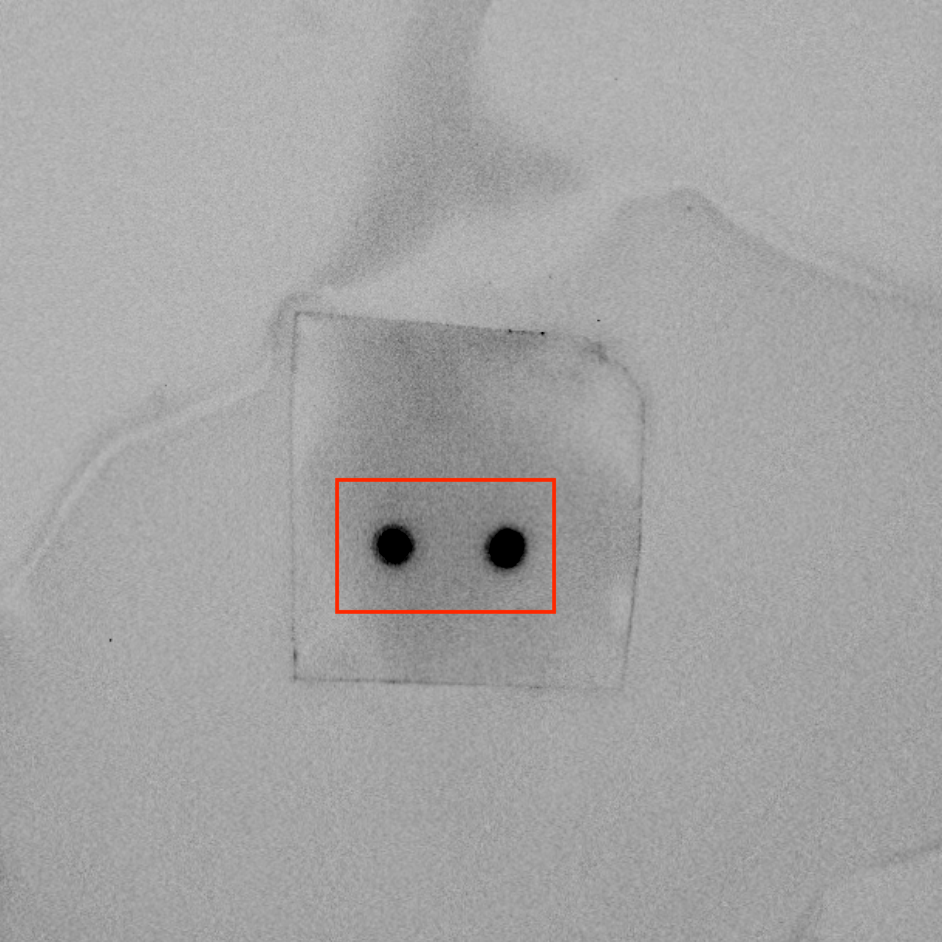

Supplement: Supplementary file 5 — Source data Fig. 4 [file 44319_2026_756_MOESM5_ESM.zip › Figure4/4D/dot blot Input-BrdU.tif]

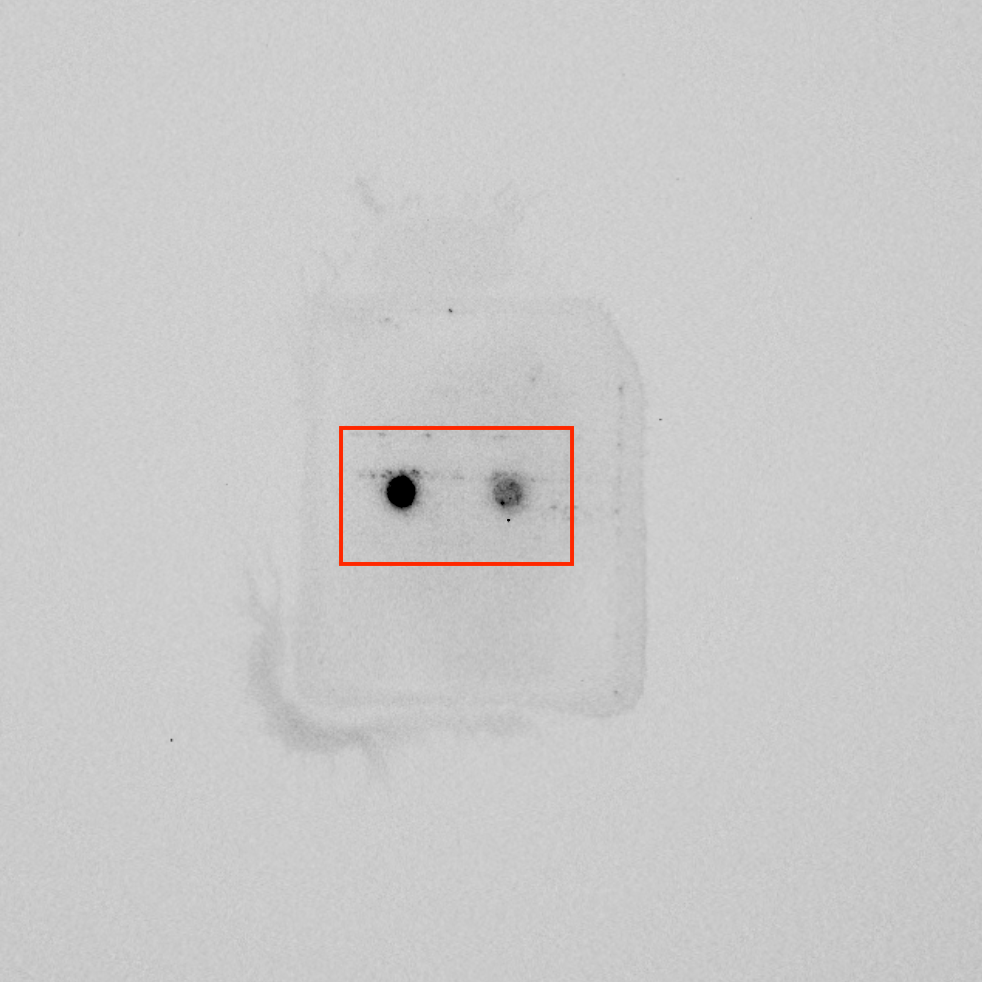

Supplement: Supplementary file 5 — Source data Fig. 4 [file 44319_2026_756_MOESM5_ESM.zip › Figure4/4D/dot blot Ip-BrdU.tif]

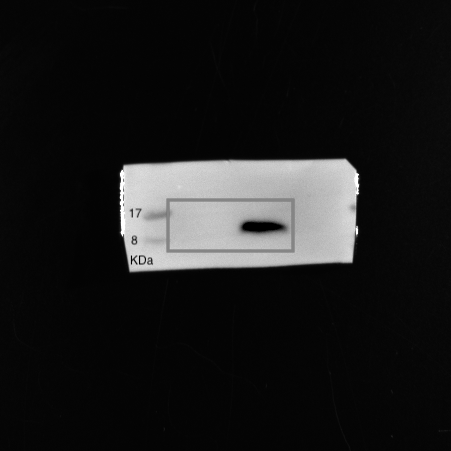

Supplement: Supplementary file 5 — Source data Fig. 4 [file 44319_2026_756_MOESM5_ESM.zip › Figure4/4D/western Input-HA.tif]

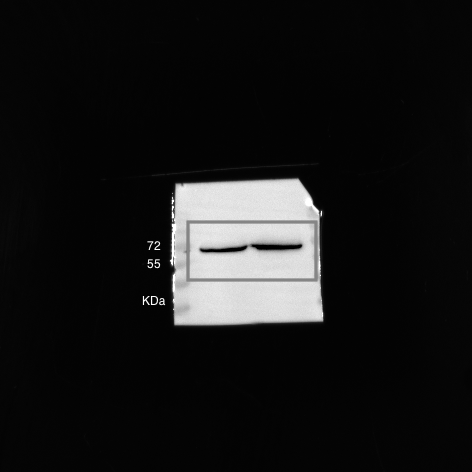

Supplement: Supplementary file 5 — Source data Fig. 4 [file 44319_2026_756_MOESM5_ESM.zip › Figure4/4D/western Input-Myc.tif]

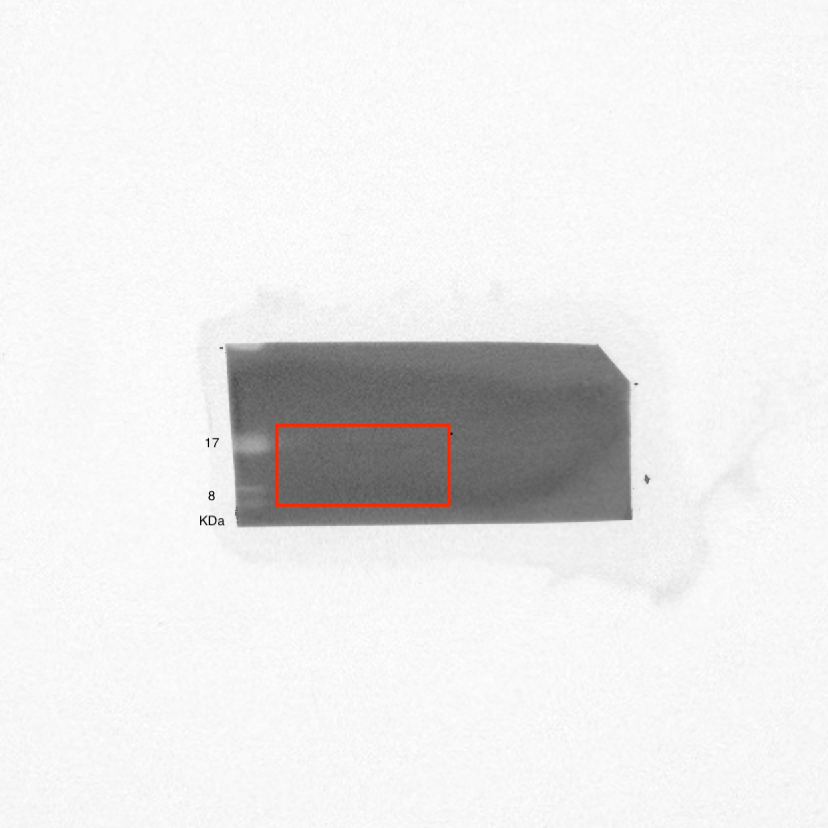

Supplement: Supplementary file 5 — Source data Fig. 4 [file 44319_2026_756_MOESM5_ESM.zip › Figure4/4D/western Ip-HA.tif]

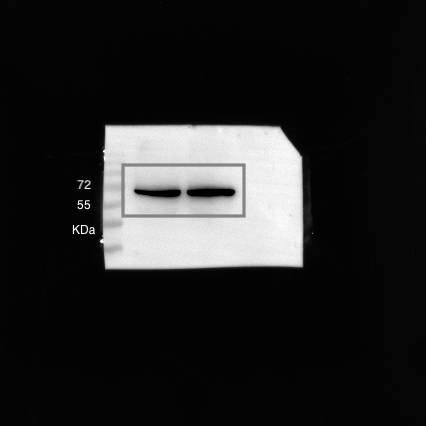

Supplement: Supplementary file 5 — Source data Fig. 4 [file 44319_2026_756_MOESM5_ESM.zip › Figure4/4D/western Ip-Myc.tif]

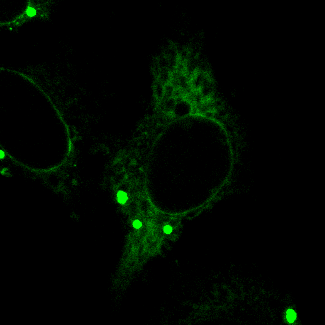

Supplement: Supplementary file 5 — Source data Fig. 4 [file 44319_2026_756_MOESM5_ESM.zip › Figure4/4F/mtDNA+cGAS/cGAS-GFP.tif]

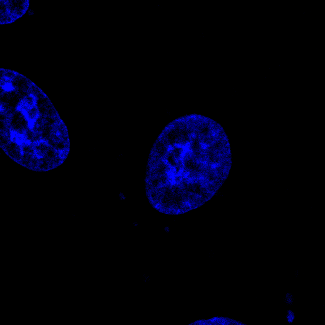

Supplement: Supplementary file 5 — Source data Fig. 4 [file 44319_2026_756_MOESM5_ESM.zip › Figure4/4F/mtDNA+cGAS/DAPI.tif]

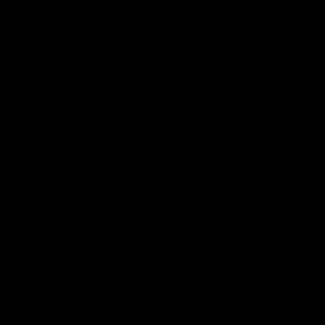

Supplement: Supplementary file 5 — Source data Fig. 4 [file 44319_2026_756_MOESM5_ESM.zip › Figure4/4F/mtDNA+cGAS/mcherry-3AB.tif]

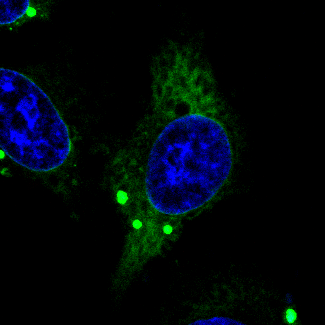

Supplement: Supplementary file 5 — Source data Fig. 4 [file 44319_2026_756_MOESM5_ESM.zip › Figure4/4F/mtDNA+cGAS/Merge.tif]

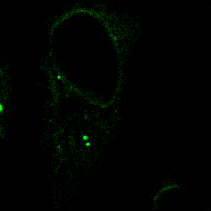

Supplement: Supplementary file 5 — Source data Fig. 4 [file 44319_2026_756_MOESM5_ESM.zip › Figure4/4F/mtDNA+cGAS+3AB/cGAS-GFP.tif]

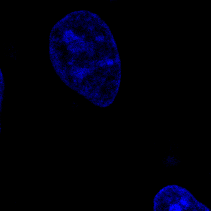

Supplement: Supplementary file 5 — Source data Fig. 4 [file 44319_2026_756_MOESM5_ESM.zip › Figure4/4F/mtDNA+cGAS+3AB/DAPI.tif]

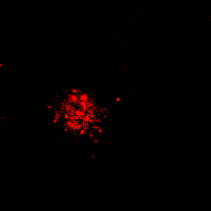

Supplement: Supplementary file 5 — Source data Fig. 4 [file 44319_2026_756_MOESM5_ESM.zip › Figure4/4F/mtDNA+cGAS+3AB/mcherry-3AB.tif]

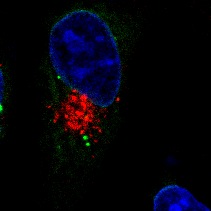

Supplement: Supplementary file 5 — Source data Fig. 4 [file 44319_2026_756_MOESM5_ESM.zip › Figure4/4F/mtDNA+cGAS+3AB/Merge.tif]

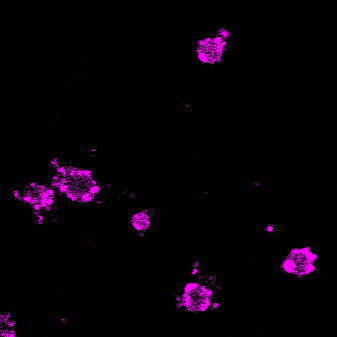

Supplement: Supplementary file 5 — Source data Fig. 4 [file 44319_2026_756_MOESM5_ESM.zip › Figure4/4H/10min/3AB.tif]

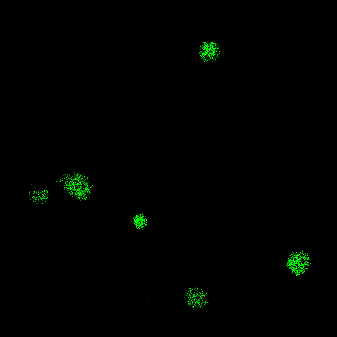

Supplement: Supplementary file 5 — Source data Fig. 4 [file 44319_2026_756_MOESM5_ESM.zip › Figure4/4H/10min/cGAS.tif]

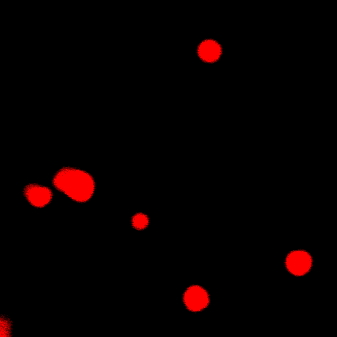

Supplement: Supplementary file 5 — Source data Fig. 4 [file 44319_2026_756_MOESM5_ESM.zip › Figure4/4H/10min/DNA.tif]

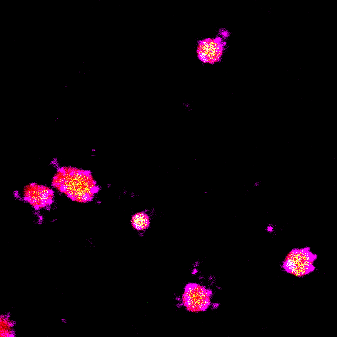

Supplement: Supplementary file 5 — Source data Fig. 4 [file 44319_2026_756_MOESM5_ESM.zip › Figure4/4H/10min/Merge.tif]

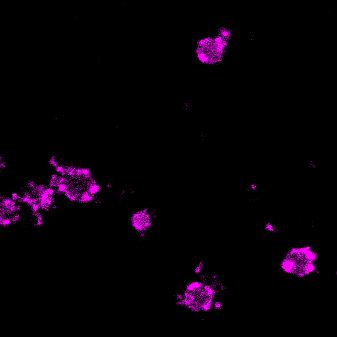

Supplement: Supplementary file 5 — Source data Fig. 4 [file 44319_2026_756_MOESM5_ESM.zip › Figure4/4H/11min/3AB.tif]

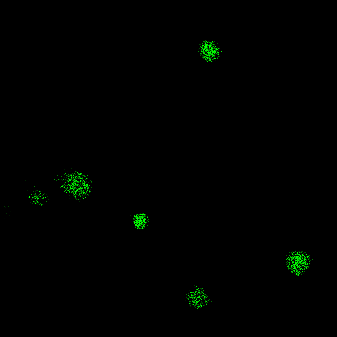

Supplement: Supplementary file 5 — Source data Fig. 4 [file 44319_2026_756_MOESM5_ESM.zip › Figure4/4H/11min/cGAS.tif]

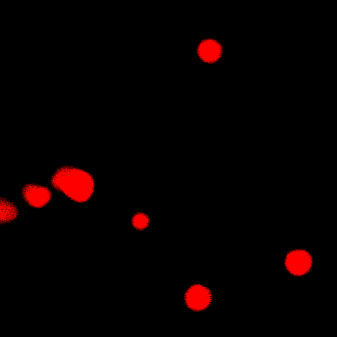

Supplement: Supplementary file 5 — Source data Fig. 4 [file 44319_2026_756_MOESM5_ESM.zip › Figure4/4H/11min/DNA.tif]

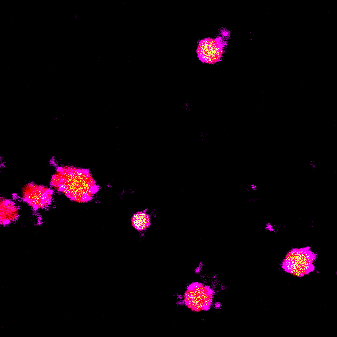

Supplement: Supplementary file 5 — Source data Fig. 4 [file 44319_2026_756_MOESM5_ESM.zip › Figure4/4H/11min/Merge.tif]

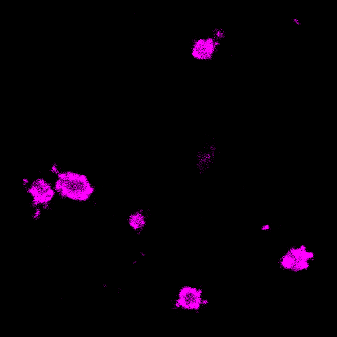

Supplement: Supplementary file 5 — Source data Fig. 4 [file 44319_2026_756_MOESM5_ESM.zip › Figure4/4H/3min/3AB.tif]

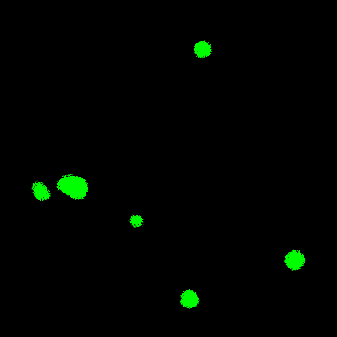

Supplement: Supplementary file 5 — Source data Fig. 4 [file 44319_2026_756_MOESM5_ESM.zip › Figure4/4H/3min/cGAS.tif]

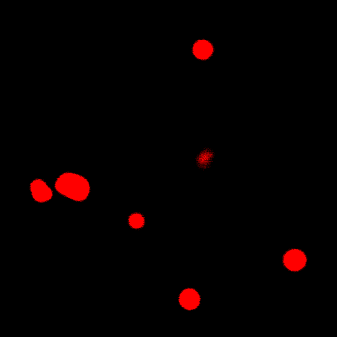

Supplement: Supplementary file 5 — Source data Fig. 4 [file 44319_2026_756_MOESM5_ESM.zip › Figure4/4H/3min/DNA.tif]

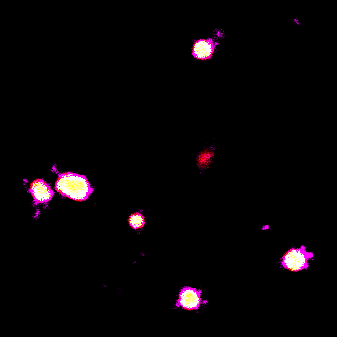

Supplement: Supplementary file 5 — Source data Fig. 4 [file 44319_2026_756_MOESM5_ESM.zip › Figure4/4H/3min/Merge.tif]

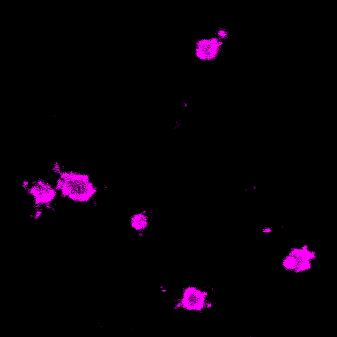

Supplement: Supplementary file 5 — Source data Fig. 4 [file 44319_2026_756_MOESM5_ESM.zip › Figure4/4H/6min/3AB.tif]

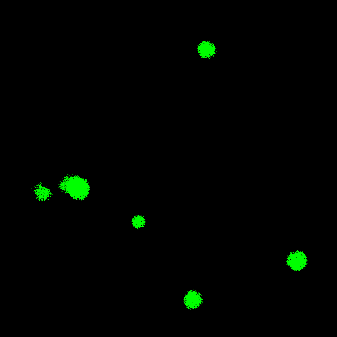

Supplement: Supplementary file 5 — Source data Fig. 4 [file 44319_2026_756_MOESM5_ESM.zip › Figure4/4H/6min/cGAS.tif]

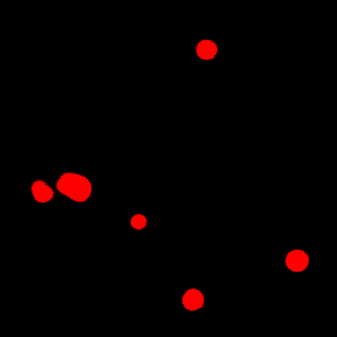

Supplement: Supplementary file 5 — Source data Fig. 4 [file 44319_2026_756_MOESM5_ESM.zip › Figure4/4H/6min/DNA.tif]

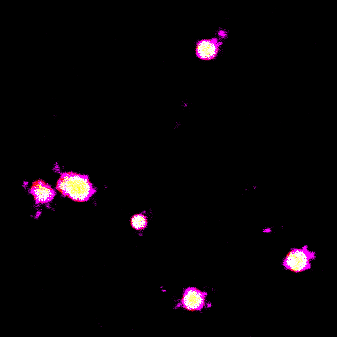

Supplement: Supplementary file 5 — Source data Fig. 4 [file 44319_2026_756_MOESM5_ESM.zip › Figure4/4H/6min/Merge.tif]

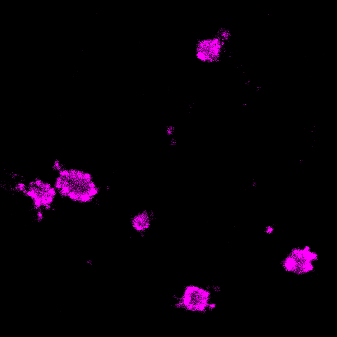

Supplement: Supplementary file 5 — Source data Fig. 4 [file 44319_2026_756_MOESM5_ESM.zip › Figure4/4H/8min/3AB.tif]

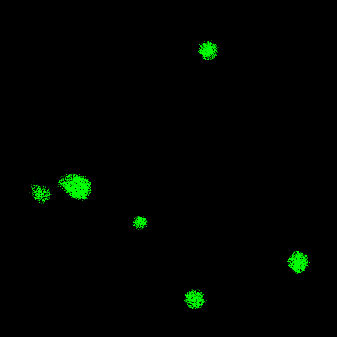

Supplement: Supplementary file 5 — Source data Fig. 4 [file 44319_2026_756_MOESM5_ESM.zip › Figure4/4H/8min/cGAS.tif]

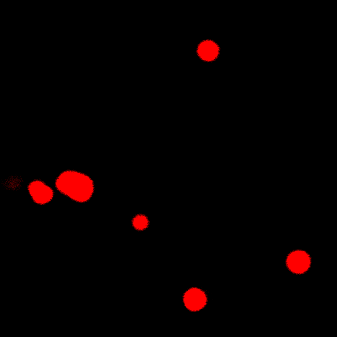

Supplement: Supplementary file 5 — Source data Fig. 4 [file 44319_2026_756_MOESM5_ESM.zip › Figure4/4H/8min/DNA.tif]

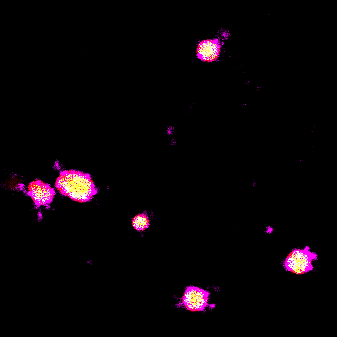

Supplement: Supplementary file 5 — Source data Fig. 4 [file 44319_2026_756_MOESM5_ESM.zip › Figure4/4H/8min/Merge.tif]

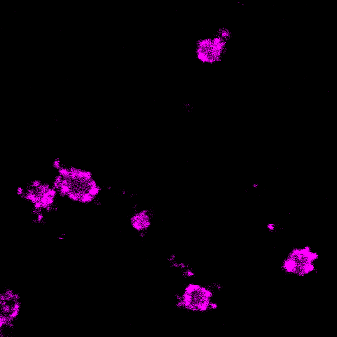

Supplement: Supplementary file 5 — Source data Fig. 4 [file 44319_2026_756_MOESM5_ESM.zip › Figure4/4H/9min/3AB.tif]

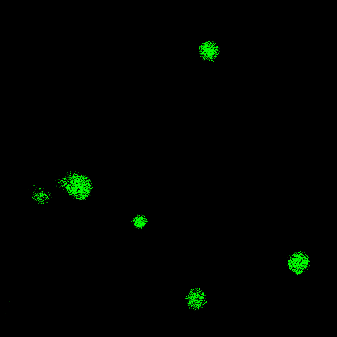

Supplement: Supplementary file 5 — Source data Fig. 4 [file 44319_2026_756_MOESM5_ESM.zip › Figure4/4H/9min/cGAS.tif]

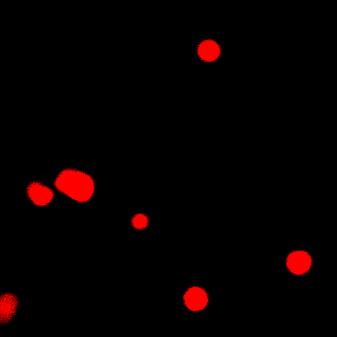

Supplement: Supplementary file 5 — Source data Fig. 4 [file 44319_2026_756_MOESM5_ESM.zip › Figure4/4H/9min/DNA.tif]

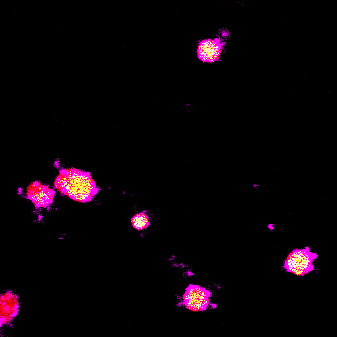

Supplement: Supplementary file 5 — Source data Fig. 4 [file 44319_2026_756_MOESM5_ESM.zip › Figure4/4H/9min/Merge.tif]

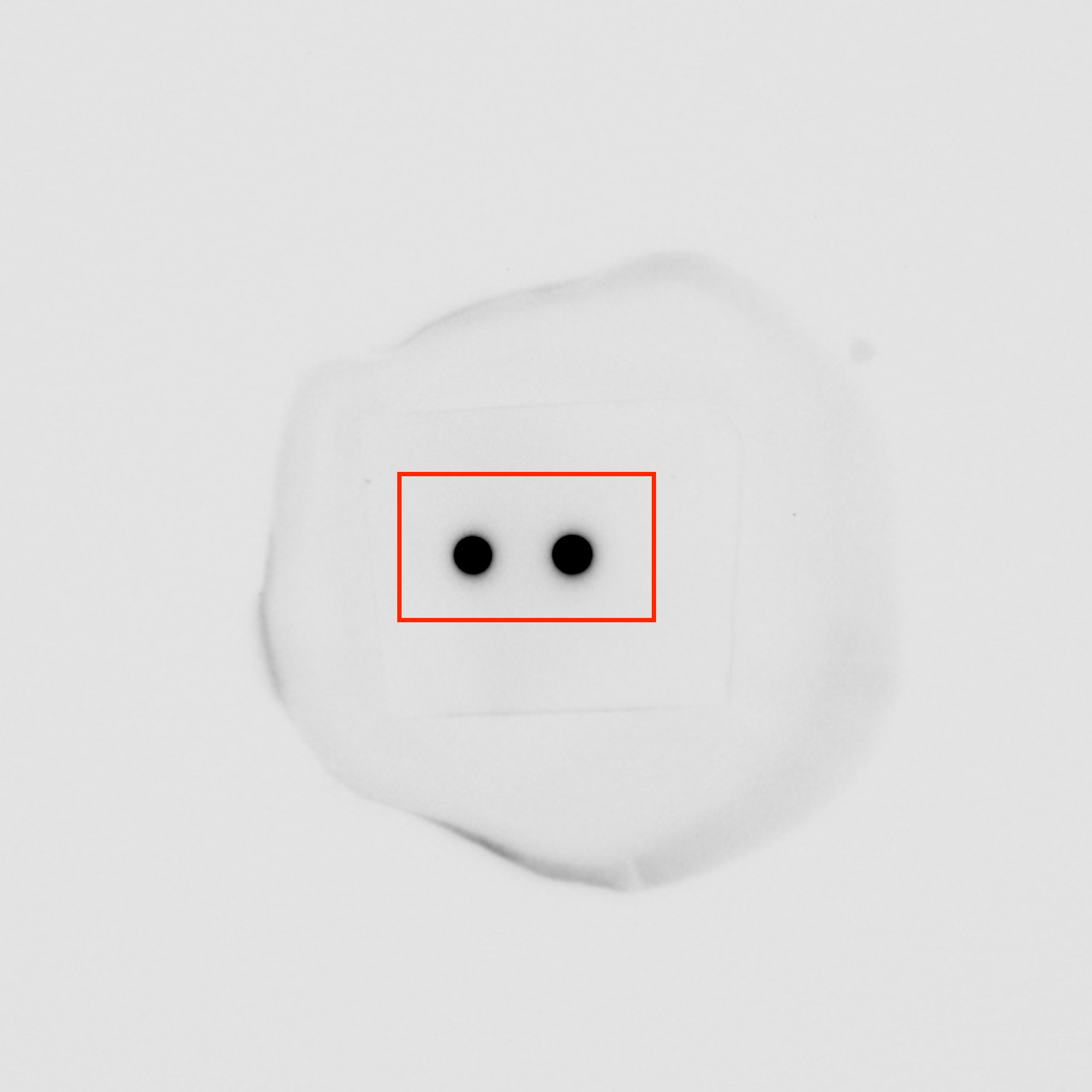

Supplement: Supplementary file 6 — Source data Fig. 5 [file 44319_2026_756_MOESM6_ESM.zip › Figure5/5B/dot blot Input-BrdU.tif]

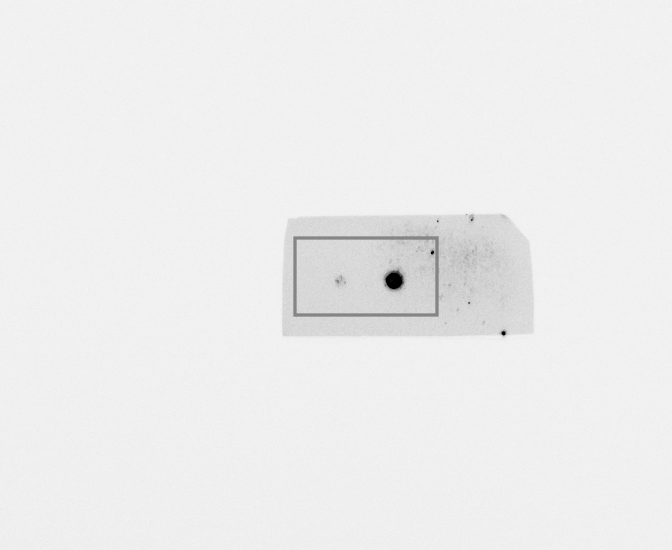

Supplement: Supplementary file 6 — Source data Fig. 5 [file 44319_2026_756_MOESM6_ESM.zip › Figure5/5B/dot blot Ip-BrdU.tif]

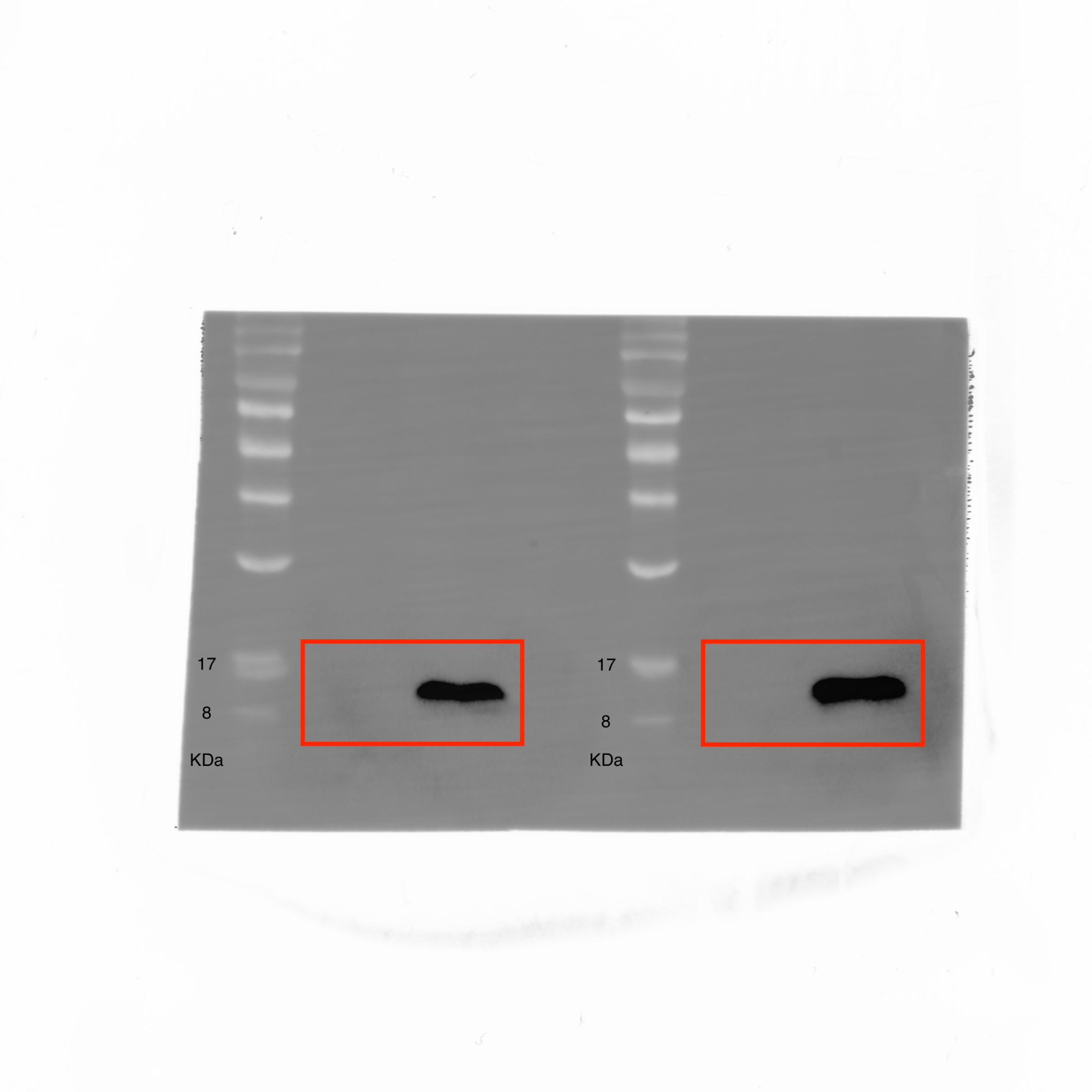

Supplement: Supplementary file 6 — Source data Fig. 5 [file 44319_2026_756_MOESM6_ESM.zip › Figure5/5B/western HA.tif]

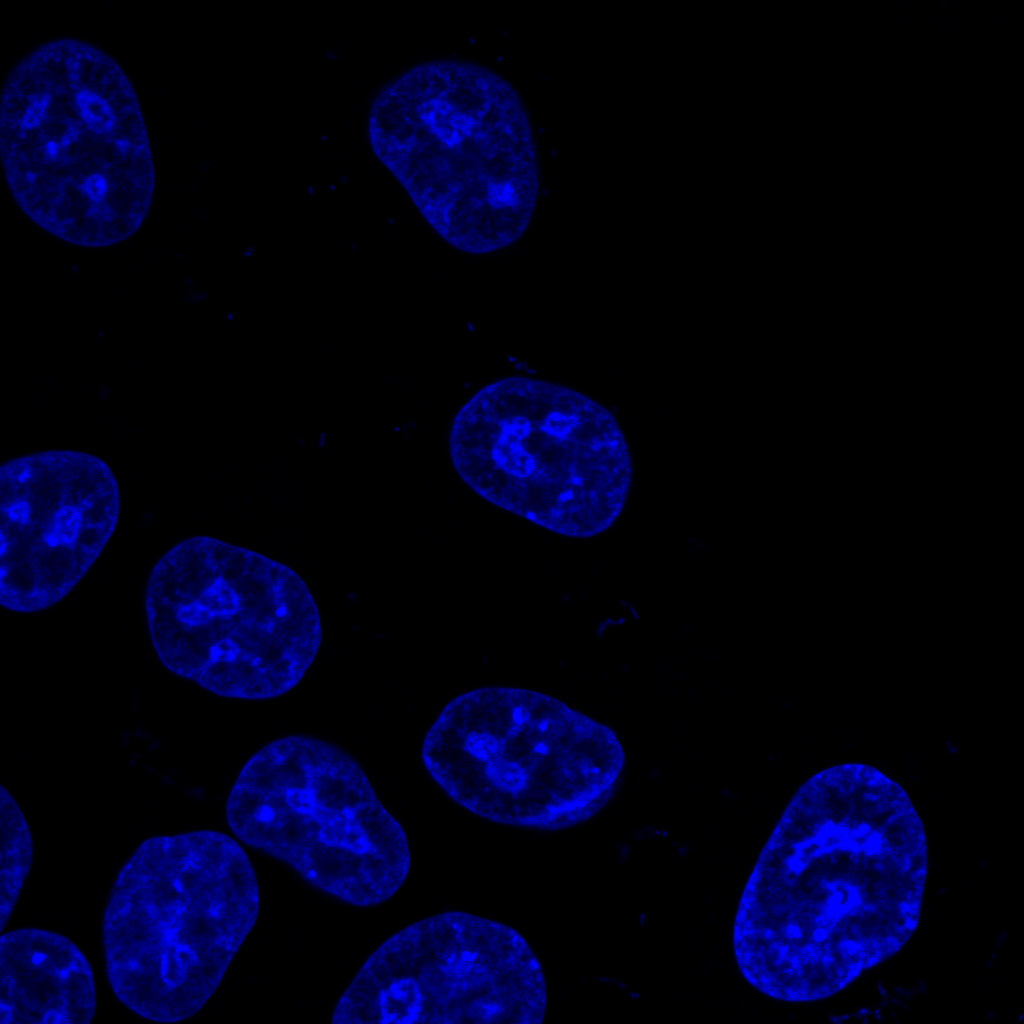

Supplement: Supplementary file 6 — Source data Fig. 5 [file 44319_2026_756_MOESM6_ESM.zip › Figure5/5C/Edu-mtDNA/DAPI.tif]

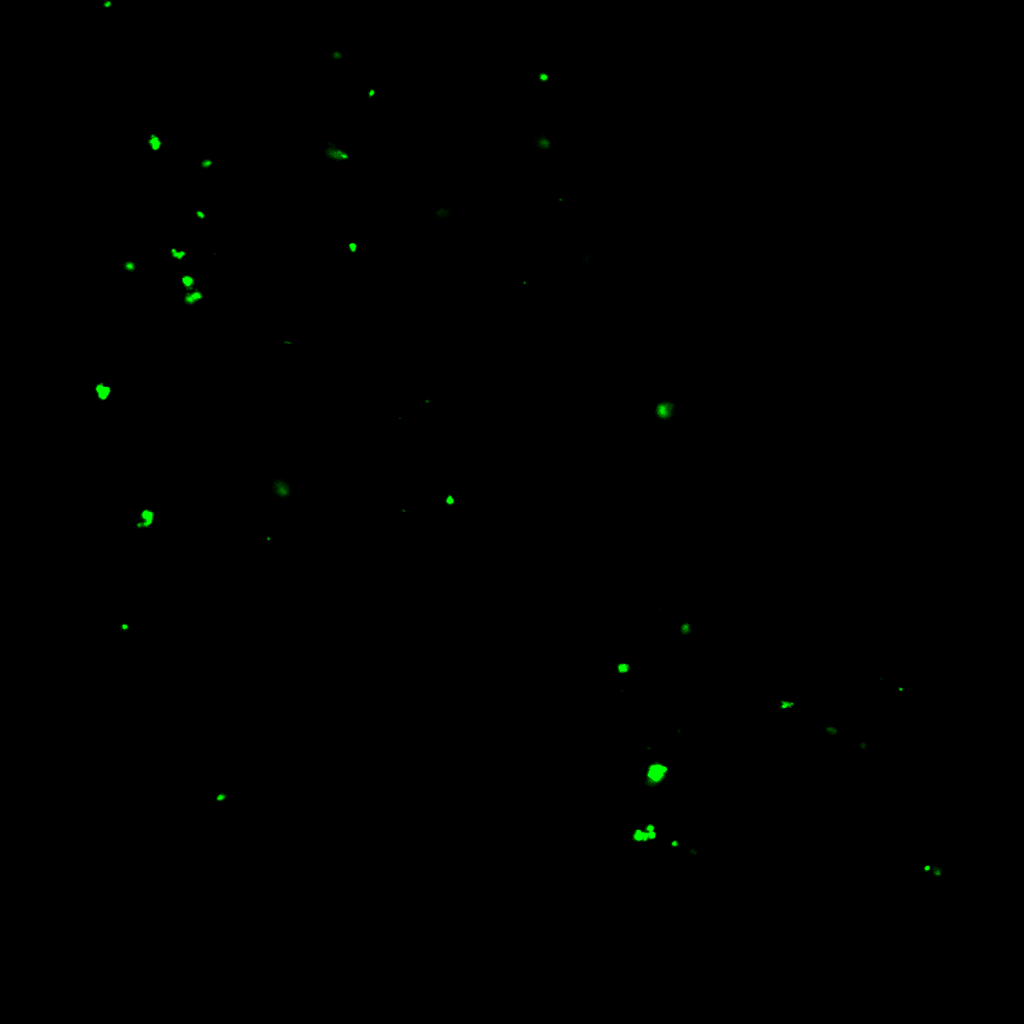

Supplement: Supplementary file 6 — Source data Fig. 5 [file 44319_2026_756_MOESM6_ESM.zip › Figure5/5C/Edu-mtDNA/EdU-mtDNA.tif]

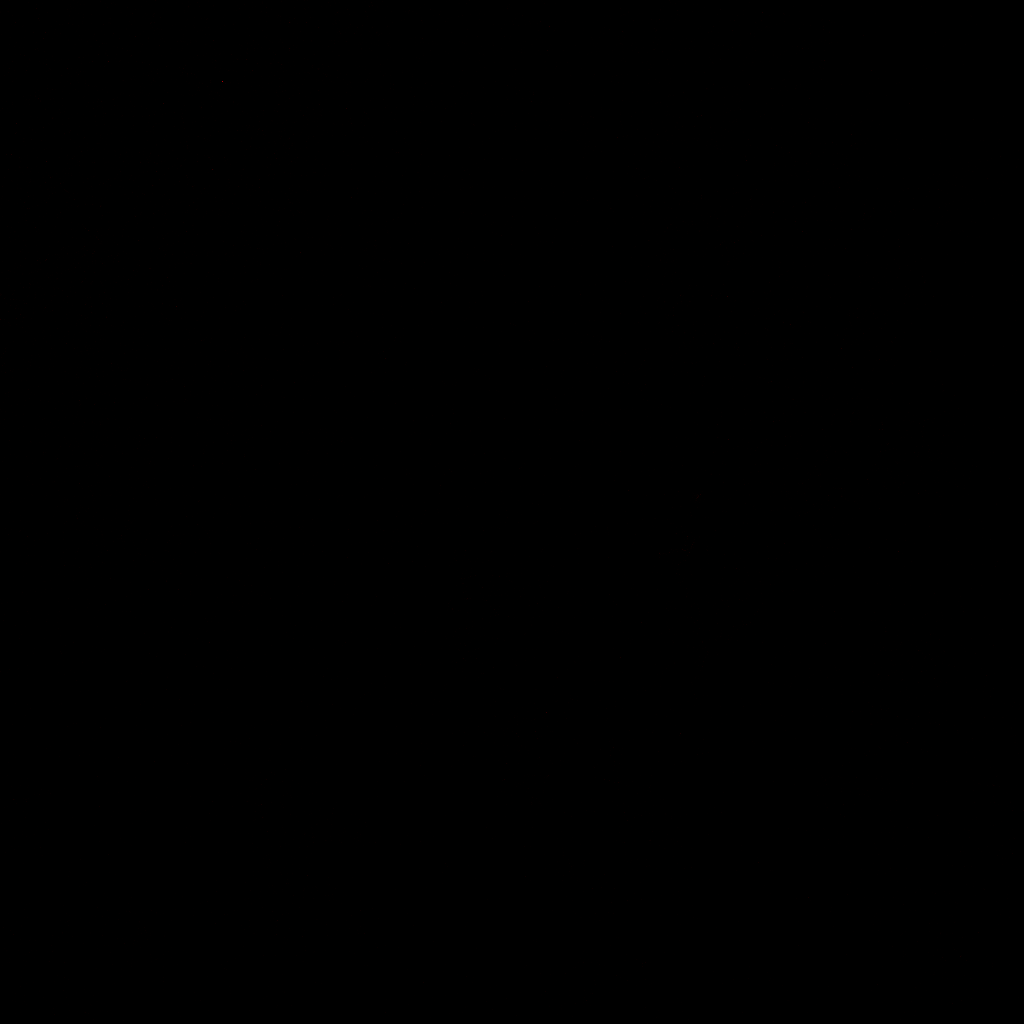

Supplement: Supplementary file 6 — Source data Fig. 5 [file 44319_2026_756_MOESM6_ESM.zip › Figure5/5C/Edu-mtDNA/mcherry-3AB.tif]

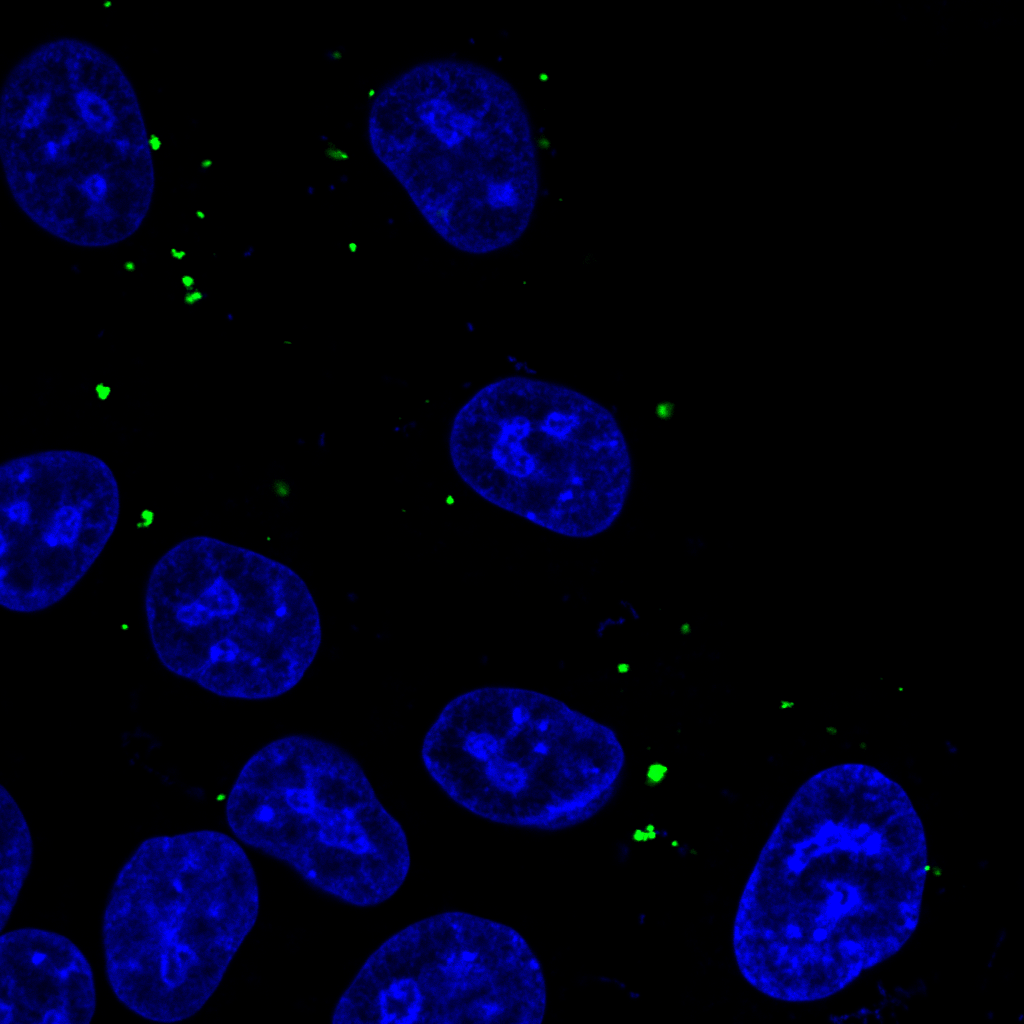

Supplement: Supplementary file 6 — Source data Fig. 5 [file 44319_2026_756_MOESM6_ESM.zip › Figure5/5C/Edu-mtDNA/Merge.tif]

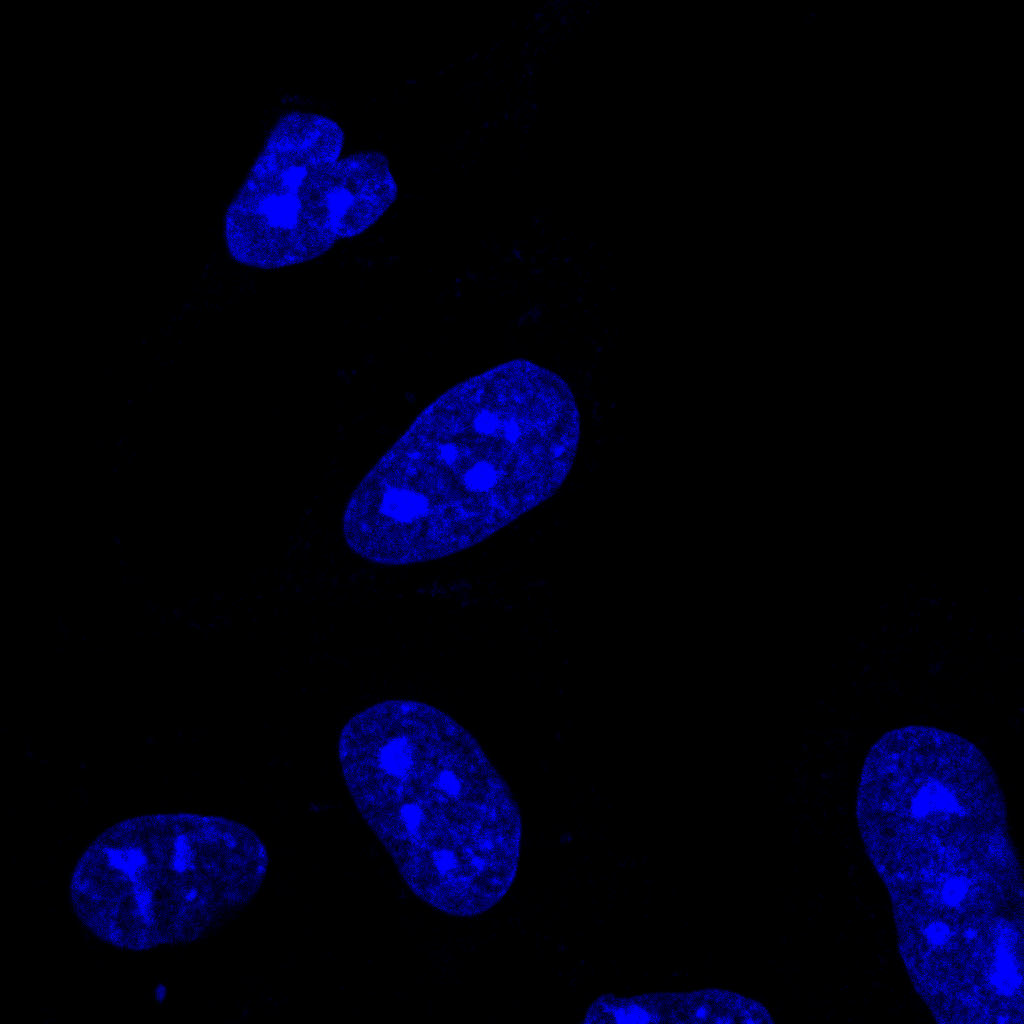

Supplement: Supplementary file 6 — Source data Fig. 5 [file 44319_2026_756_MOESM6_ESM.zip › Figure5/5C/Edu-mtDNA+mcherry-3AB/DAPI.tif]

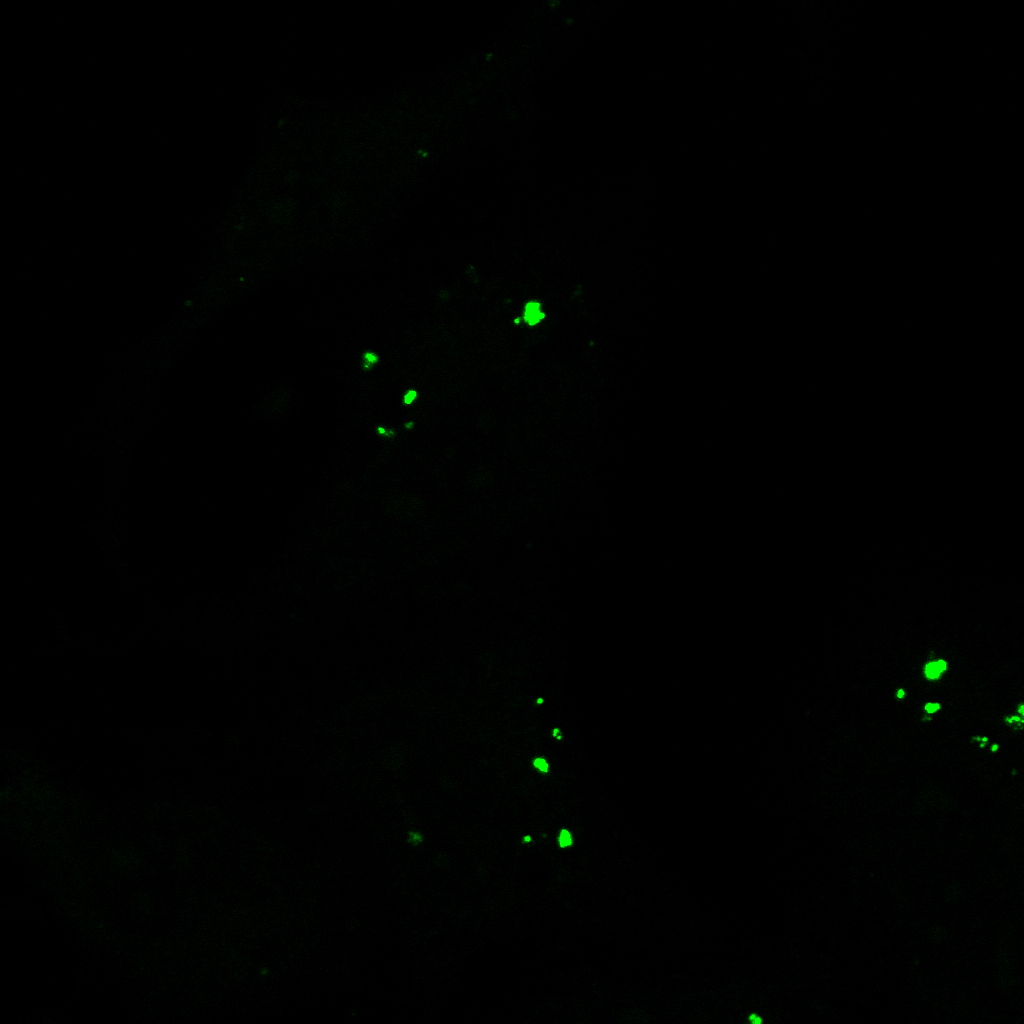

Supplement: Supplementary file 6 — Source data Fig. 5 [file 44319_2026_756_MOESM6_ESM.zip › Figure5/5C/Edu-mtDNA+mcherry-3AB/EdU-mtDNA.tif]

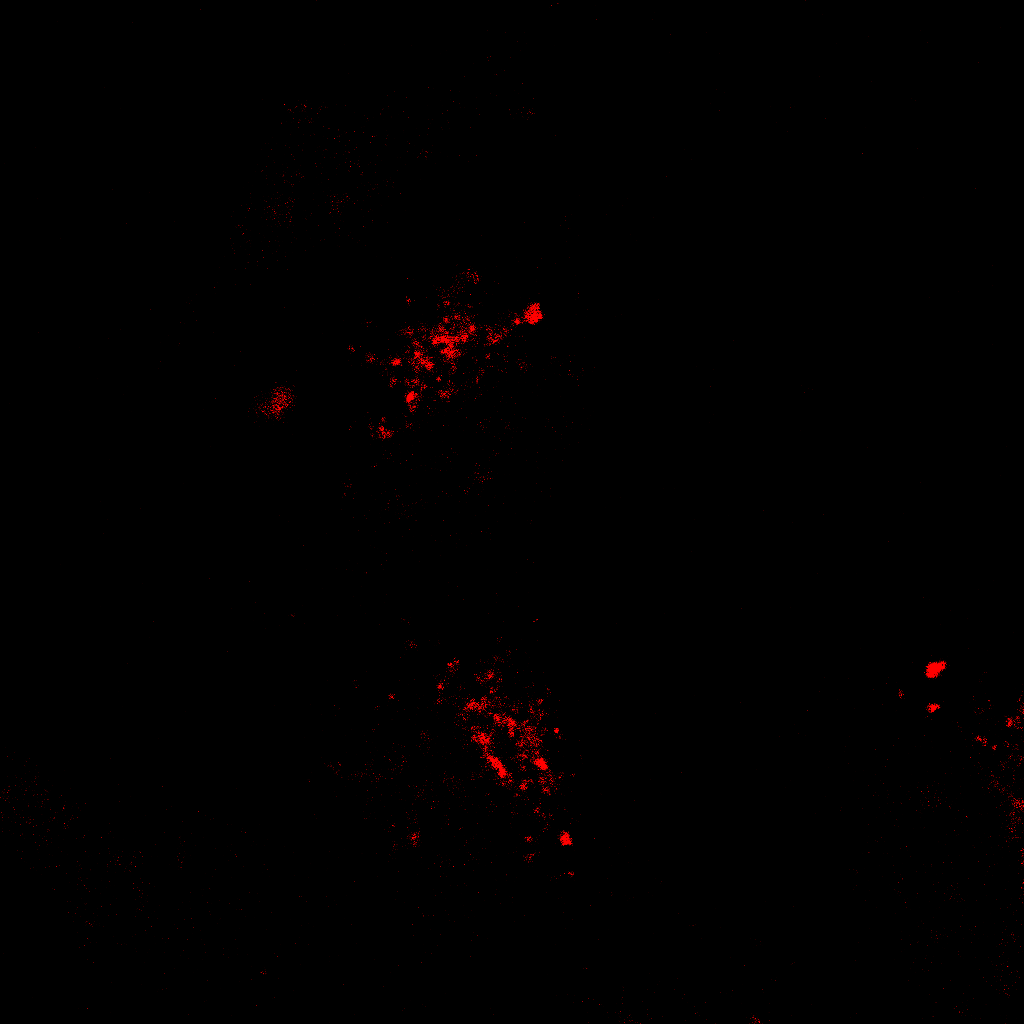

Supplement: Supplementary file 6 — Source data Fig. 5 [file 44319_2026_756_MOESM6_ESM.zip › Figure5/5C/Edu-mtDNA+mcherry-3AB/mcherry-3AB.tif]

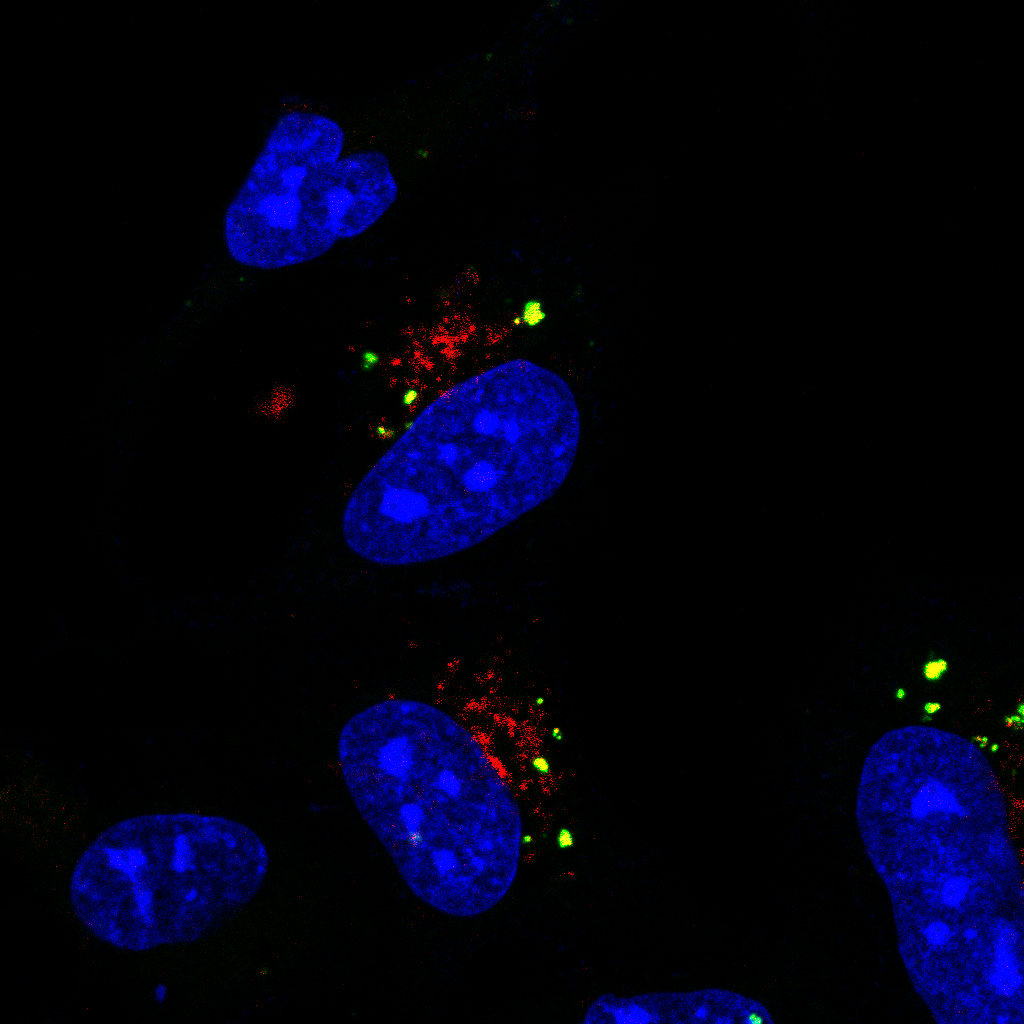

Supplement: Supplementary file 6 — Source data Fig. 5 [file 44319_2026_756_MOESM6_ESM.zip › Figure5/5C/Edu-mtDNA+mcherry-3AB/Merge.tif]

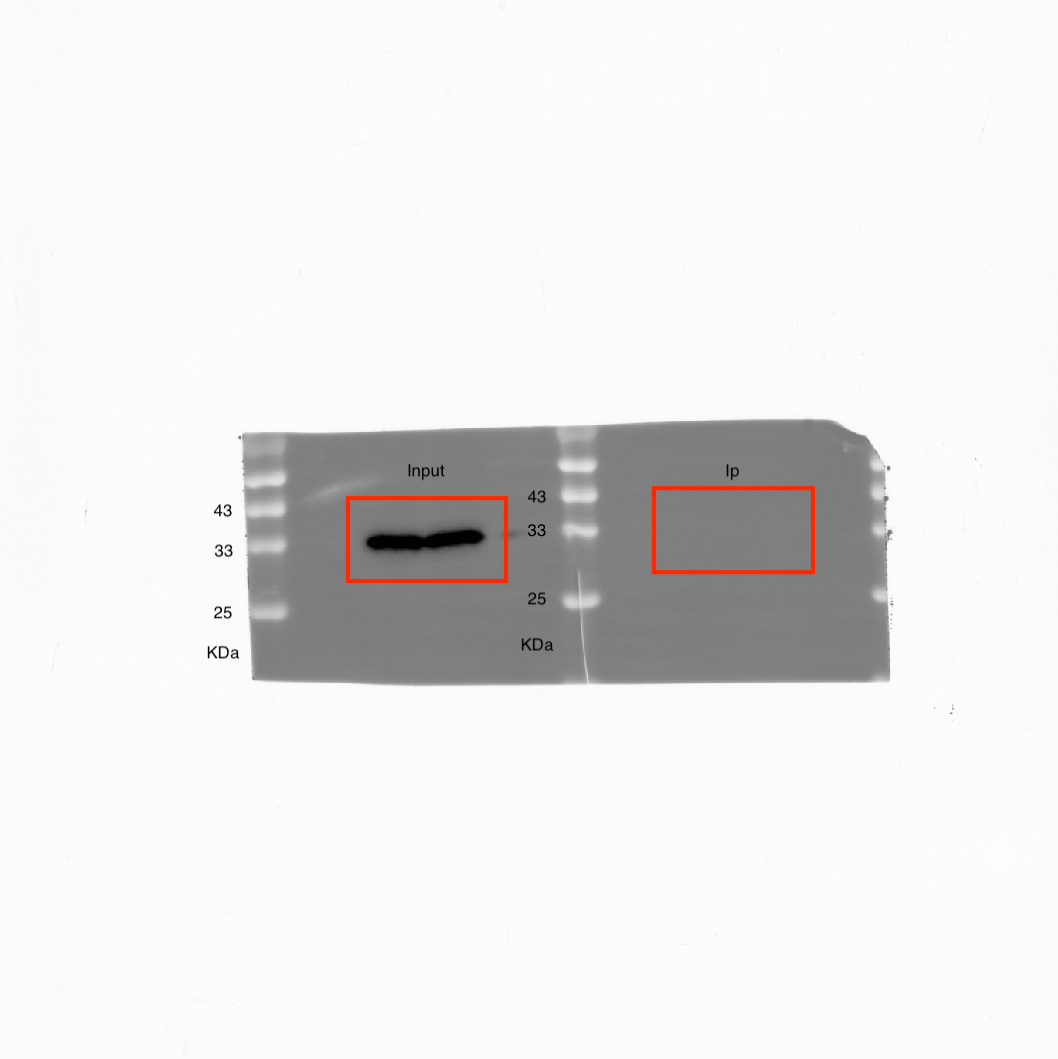

Supplement: Supplementary file 6 — Source data Fig. 5 [file 44319_2026_756_MOESM6_ESM.zip › Figure5/5E/western GAPDH.tif]

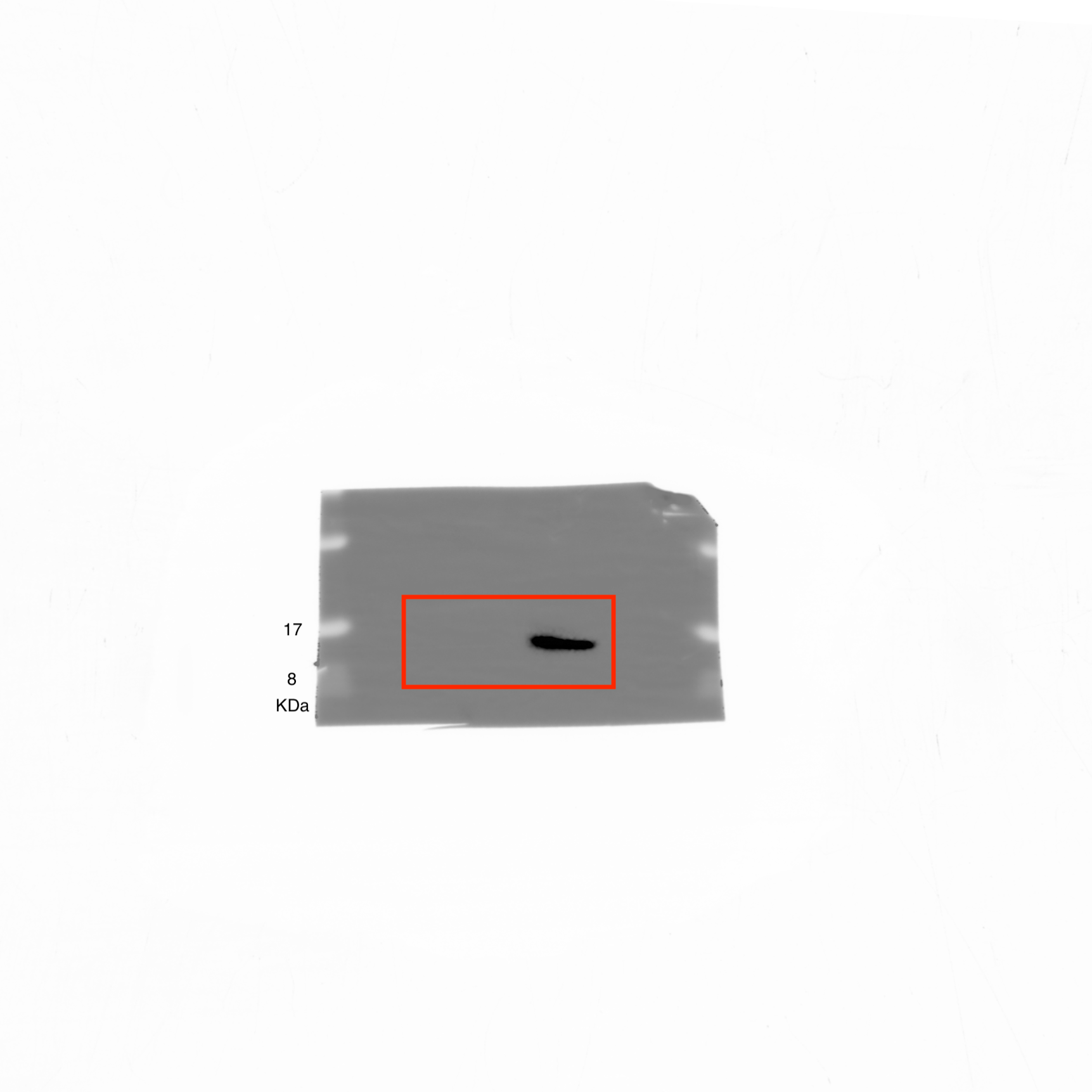

Supplement: Supplementary file 6 — Source data Fig. 5 [file 44319_2026_756_MOESM6_ESM.zip › Figure5/5E/western Input EV-A71-3AB.tif]

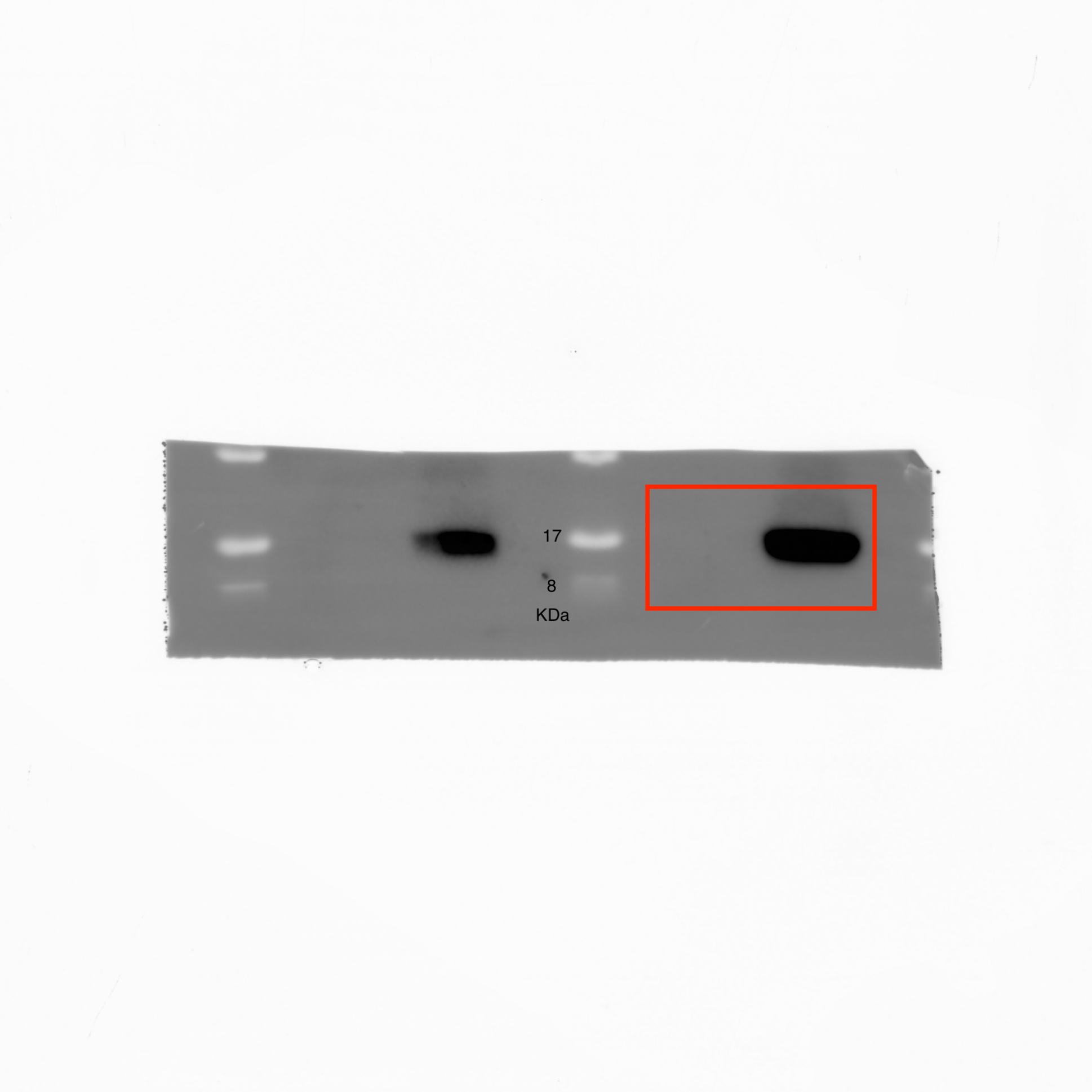

Supplement: Supplementary file 6 — Source data Fig. 5 [file 44319_2026_756_MOESM6_ESM.zip › Figure5/5E/western Ip EV-A71-3AB.tif]

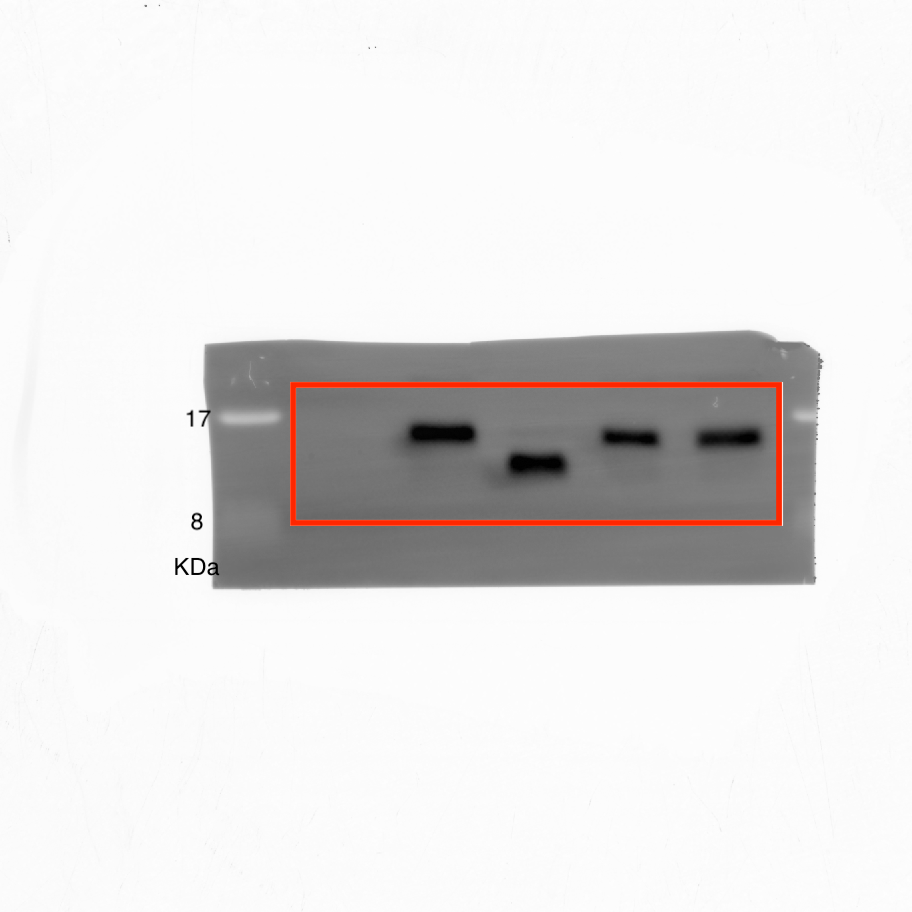

Supplement: Supplementary file 7 — Source data Fig. 6 [file 44319_2026_756_MOESM7_ESM.zip › Figure6/6B/western Anti-HA.tif]

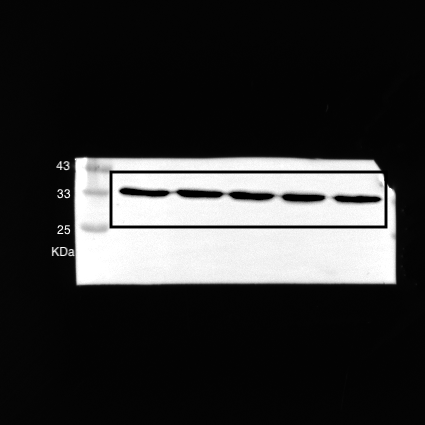

Supplement: Supplementary file 7 — Source data Fig. 6 [file 44319_2026_756_MOESM7_ESM.zip › Figure6/6B/western GAPDH.tif]

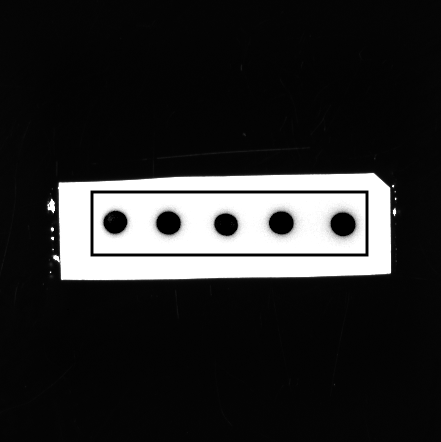

Supplement: Supplementary file 7 — Source data Fig. 6 [file 44319_2026_756_MOESM7_ESM.zip › Figure6/6C/dot blot Input-BrdU.tif]

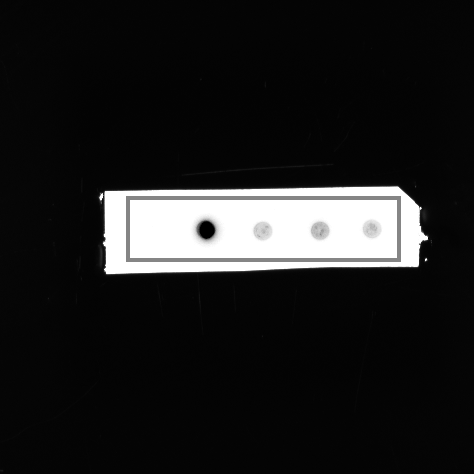

Supplement: Supplementary file 7 — Source data Fig. 6 [file 44319_2026_756_MOESM7_ESM.zip › Figure6/6C/dot blot Ip-BrdU.tif]

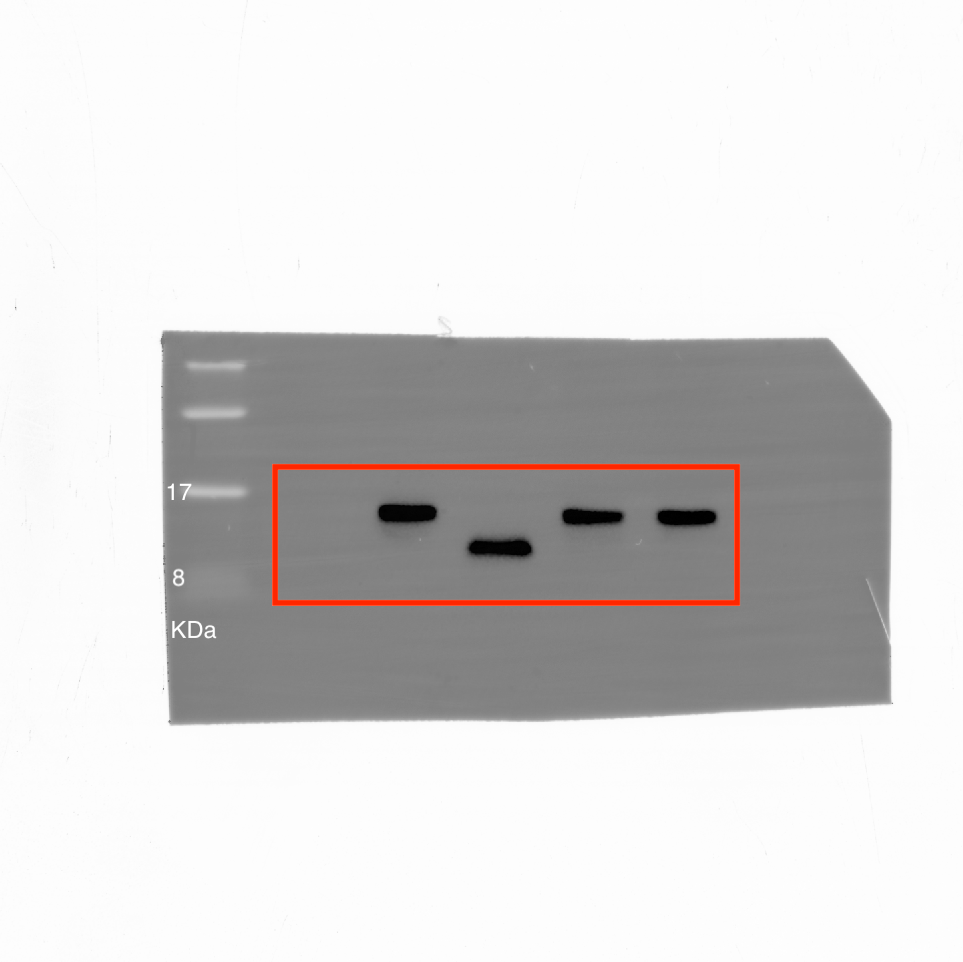

Supplement: Supplementary file 7 — Source data Fig. 6 [file 44319_2026_756_MOESM7_ESM.zip › Figure6/6C/western Input-HA.tif]

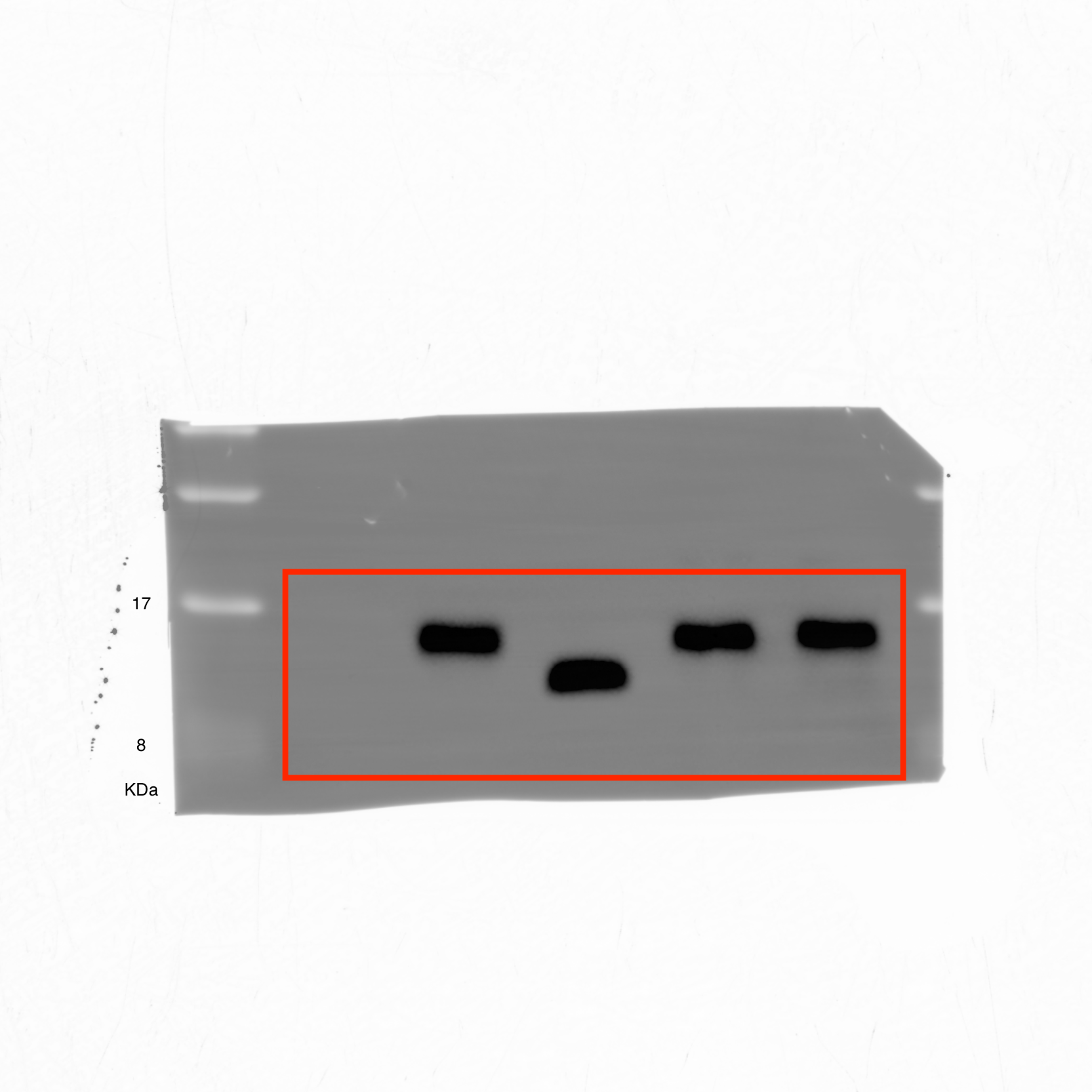

Supplement: Supplementary file 7 — Source data Fig. 6 [file 44319_2026_756_MOESM7_ESM.zip › Figure6/6C/western Ip-HA.tif]

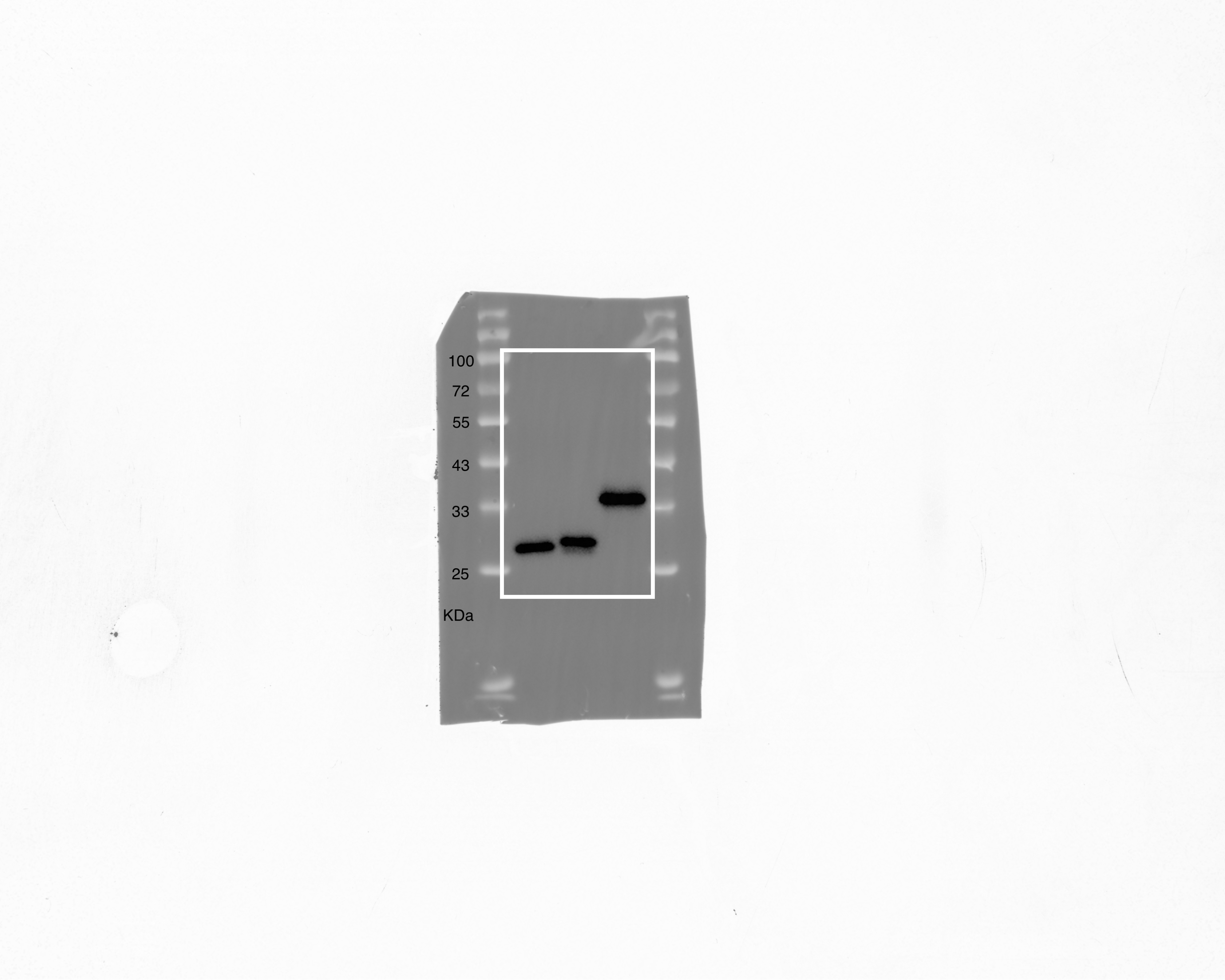

Supplement: Supplementary file 7 — Source data Fig. 6 [file 44319_2026_756_MOESM7_ESM.zip › Figure6/6F/Anti-GST.tif]

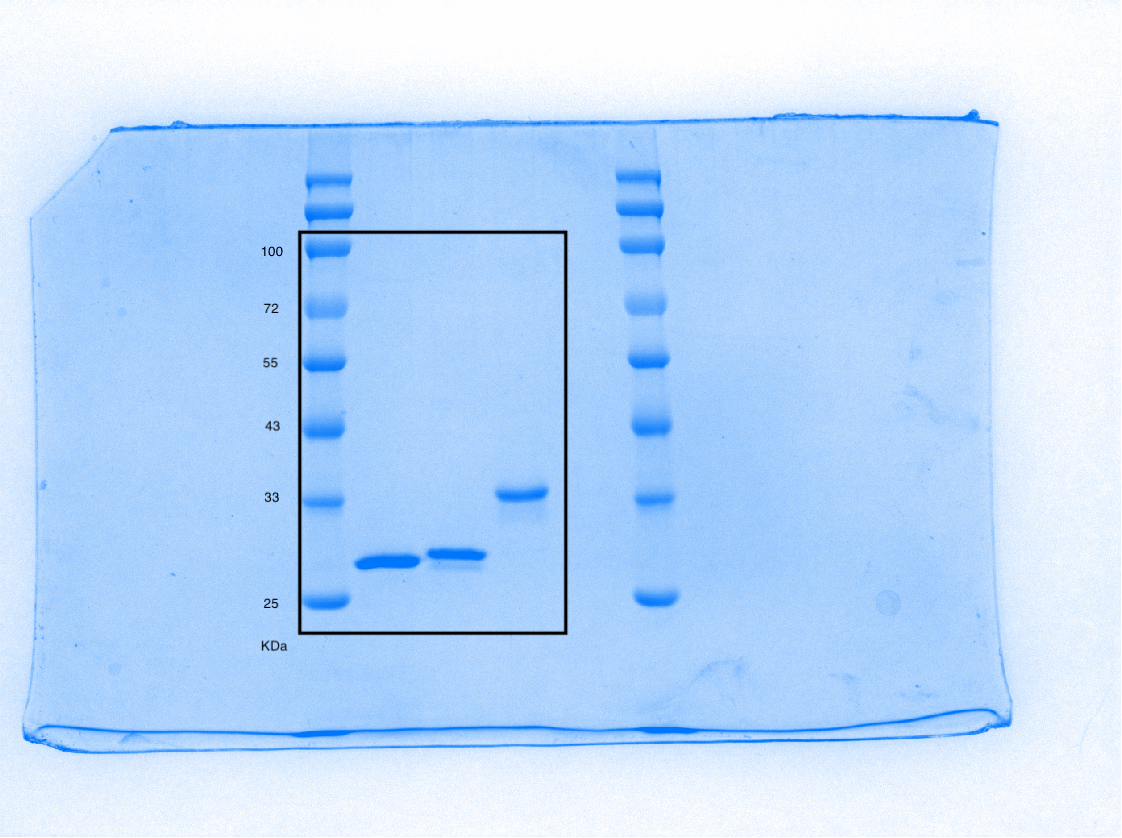

Supplement: Supplementary file 7 — Source data Fig. 6 [file 44319_2026_756_MOESM7_ESM.zip › Figure6/6F/SDS-PAGE.tif]

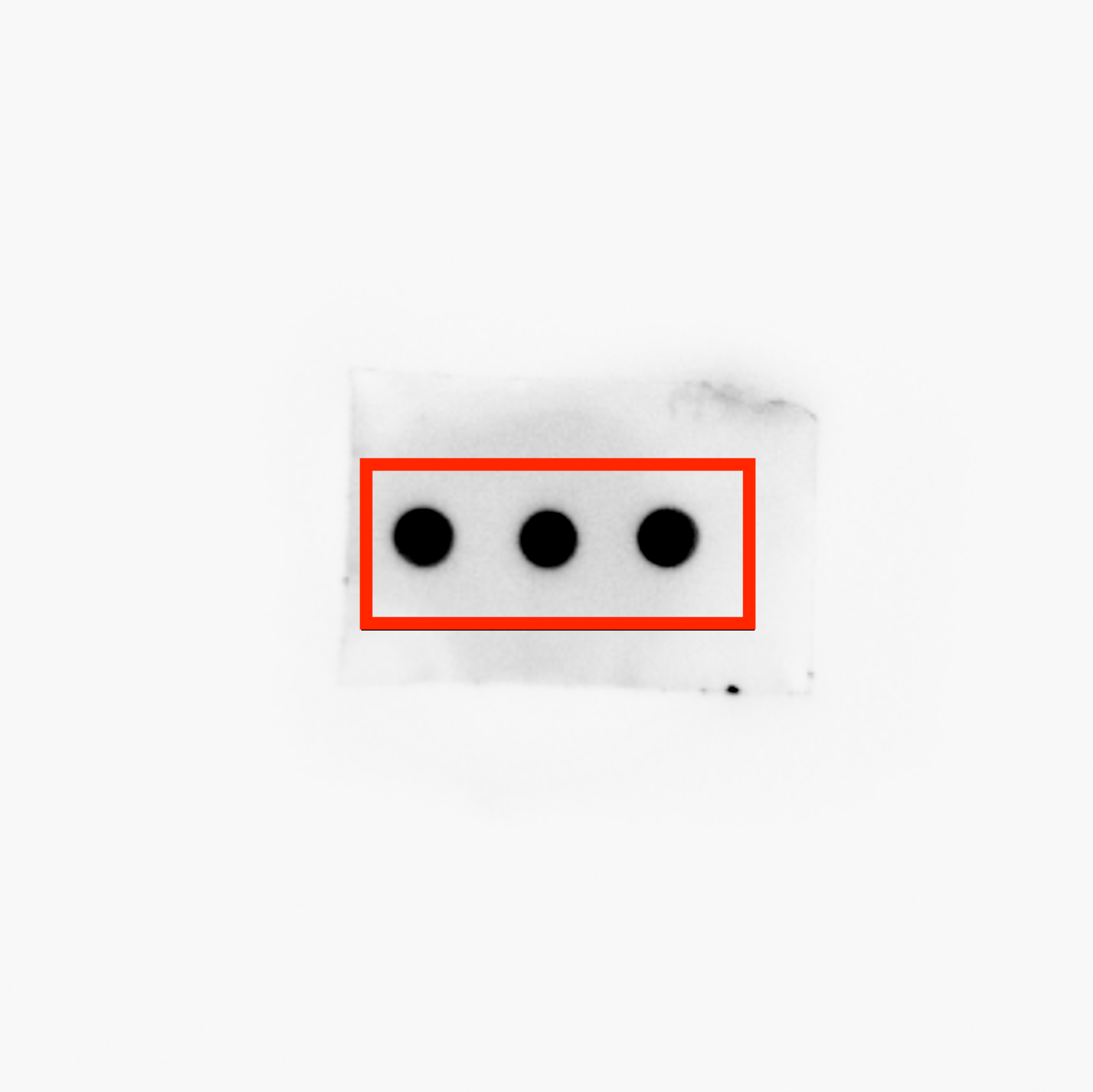

Supplement: Supplementary file 7 — Source data Fig. 6 [file 44319_2026_756_MOESM7_ESM.zip › Figure6/6H/dot blot Input-BrdU.tif]

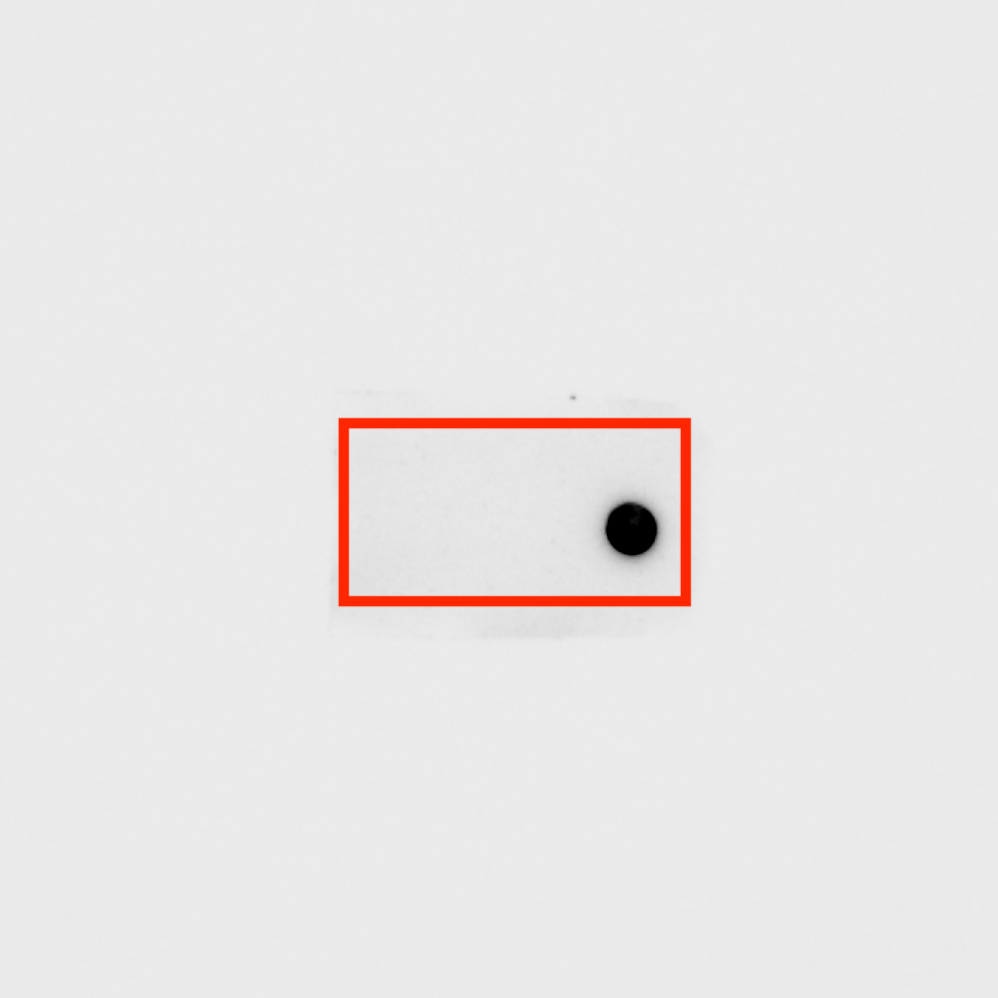

Supplement: Supplementary file 7 — Source data Fig. 6 [file 44319_2026_756_MOESM7_ESM.zip › Figure6/6H/dot blot Ip-BrdU.tif]

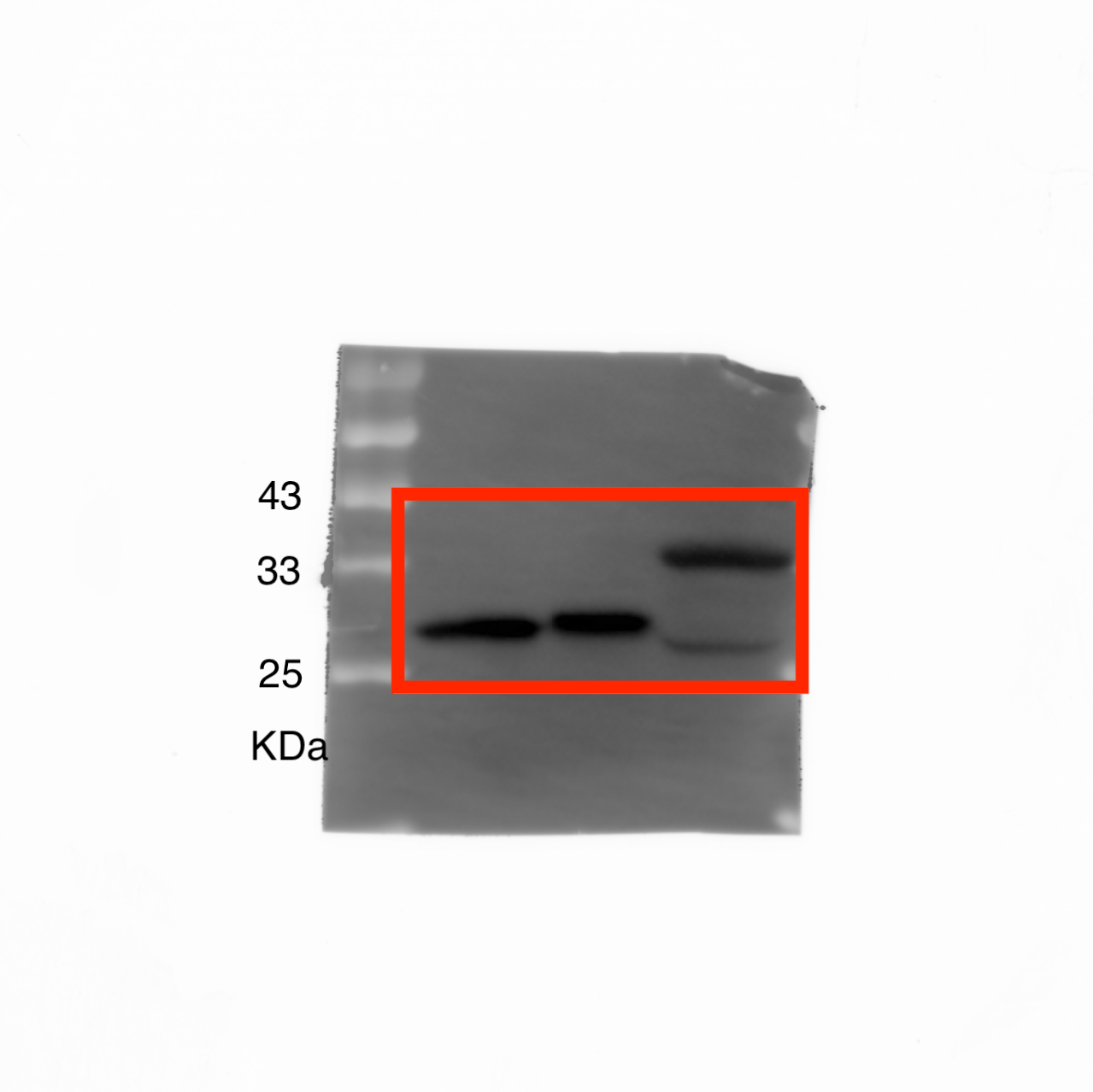

Supplement: Supplementary file 7 — Source data Fig. 6 [file 44319_2026_756_MOESM7_ESM.zip › Figure6/6H/western Input-GST.tif]

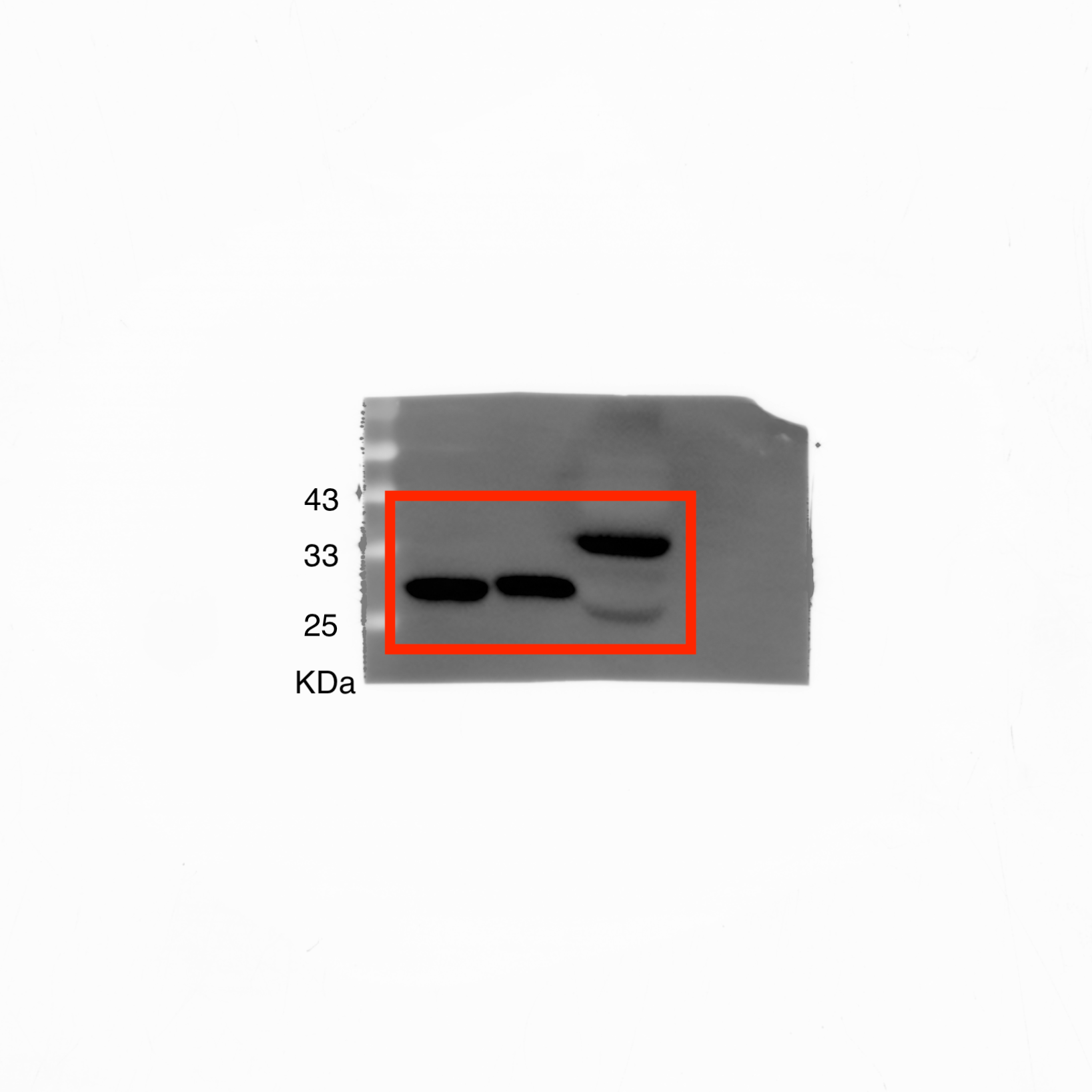

Supplement: Supplementary file 7 — Source data Fig. 6 [file 44319_2026_756_MOESM7_ESM.zip › Figure6/6H/western Ip-GST.tif]

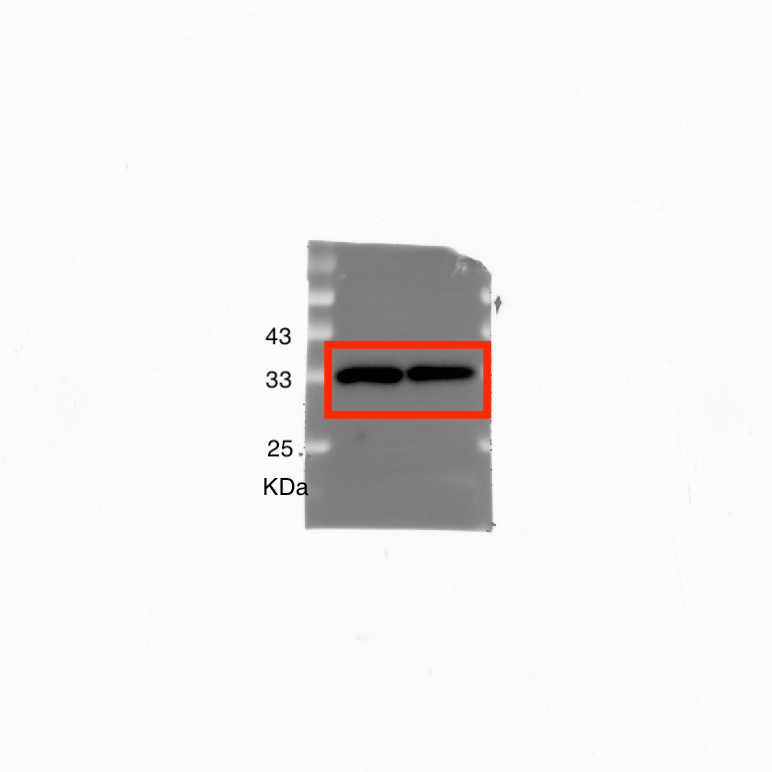

Supplement: Supplementary file 8 — Source data Fig. 7 [file 44319_2026_756_MOESM8_ESM.zip › Figure7/7B/western GAPDH.tif]

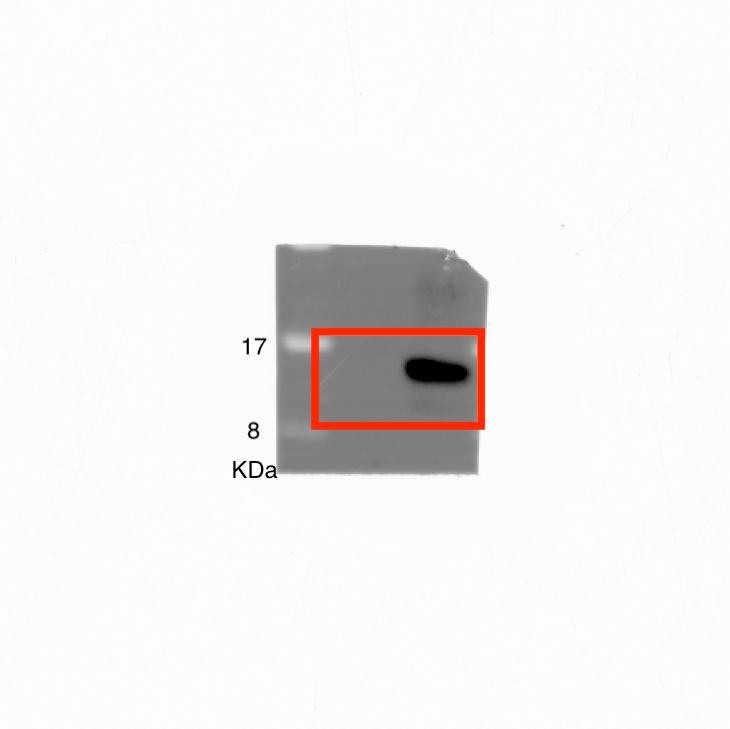

Supplement: Supplementary file 8 — Source data Fig. 7 [file 44319_2026_756_MOESM8_ESM.zip › Figure7/7B/western HA.tif]
