# Supplementary material for: Enterovirus A71 3AB protein facilitates immune evasion by blocking cGAS recognition of mtDNA
Source: EMBO Rep. 2026 Apr 2;27(10):2675–702. doi: 10.1038/s44319-026-00756-x (PMC13219398; doi:10.1038/s44319-026-00756-x)
Supplement: Supplementary file 14 — Expanded View Figures [file 44319_2026_756_MOESM14_ESM.pdf]

Expanded View Figures

Figure EV1. EV-A71 2B protein induces mitochondrial damage.

(**A–D**) HeLa, U251, RD, and THP-1 cells were infected with EV-A71 (MOI = 1) for 12, 12, 6, and 24 h, respectively. Cytosolic fractions were isolated, and whole-cell lysates (WCL) or cytosolic fractions (Cyt) were analyzed by Western blotting. (**E**) HeLa cells were infected with EVA-71-UV for 12 h. Cytosolic fractions were isolated, and qPCR was conducted to measure the cytosolic mtDNA. Data were presented as mean ± SEM, $n = 3$ biological replicates. Unpaired *t* test was used for statistical analysis. (**F**) Mitochondrial morphology analyzed in RD cells transfected with EV-A71 2B plasmid. After 18 h of EV-A71 2B transfection in RD cells, mitochondrial morphology was examined by transmission electron microscopy. Scale bars, 1 µm. (**G**) After 18 h of EV-A71 2B plasmid transfection in HeLa or RD cells, the mitochondrial membrane potential was assessed with JC-10, with CCCP serving as a positive control. Images were examined with a Leica STELLARIS 5 confocal microscope (left panel). Scale bars, 10 µm. The ratio of red to green fluorescence for JC-10 was analyzed with a multifunctional microplate reader (right panel). Data were presented as mean ± SEM, $n = 3$ biological replicates. Unpaired *t* test was used for statistical analysis. (**H**) HeLa cells were transfected with the EV-A71 2B plasmid for 24 h. Western blotting analysis was then conducted to assess the levels of phosphorylated (p-) and total TBK1, as well as total cGAS, STING and IRF3. The experiments were repeated at least three times with the similar results. Source data are available online for this figure.

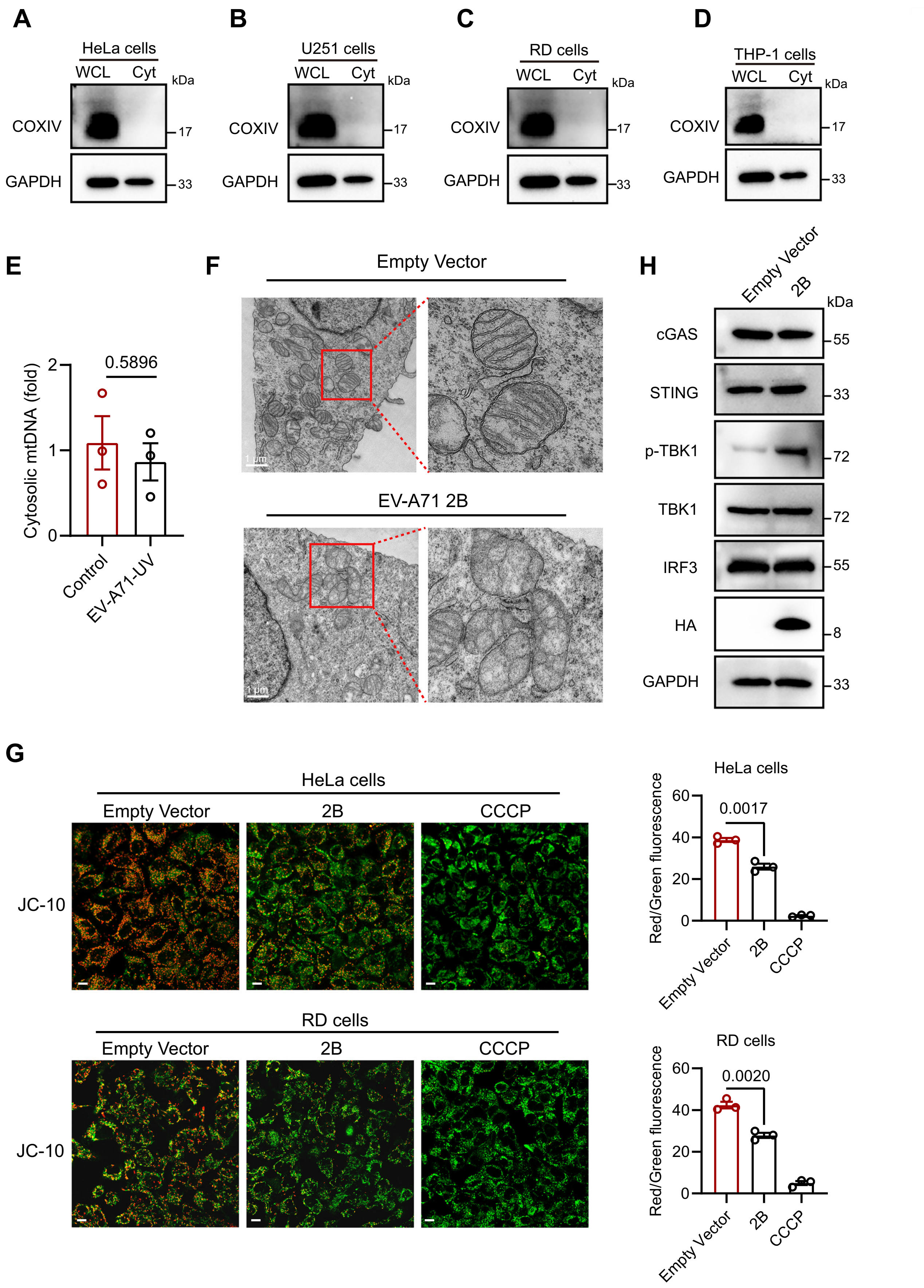

A
HeLa cells
WCL Cyt
kDa
COXIV
17
GAPDH
33
B
U251 cells
WCL Cyt
kDa
COXIV
17
GAPDH
33
C
RD cells
WCL Cyt
kDa
COXIV
17
GAPDH
33
D
THP-1 cells
WCL Cyt
kDa
COXIV
17
GAPDH
33
E
Cytosolic mtDNA (fold)
0.5896
2
1
0
Control
EV-A71-UV
F
Empty Vector
EV-A71 2B
1 µm
H
Empty Vector
2B
kDa
cGAS
55
STING
33
p-TBK1
72
TBK1
72
IRF3
55
HA
8
GAPDH
33
G
HeLa cells
Empty Vector
2B
CCCP
JC-10
RD cells
Empty Vector
2B
CCCP
JC-10
HeLa cells
Red/Green fluorescence
60
40
20
0
0.0017
Empty Vector
2B
CCCP
RD cells
Red/Green fluorescence
60
40
20
0
0.0020
Empty Vector
2B
CCCP

 © The Author(s)

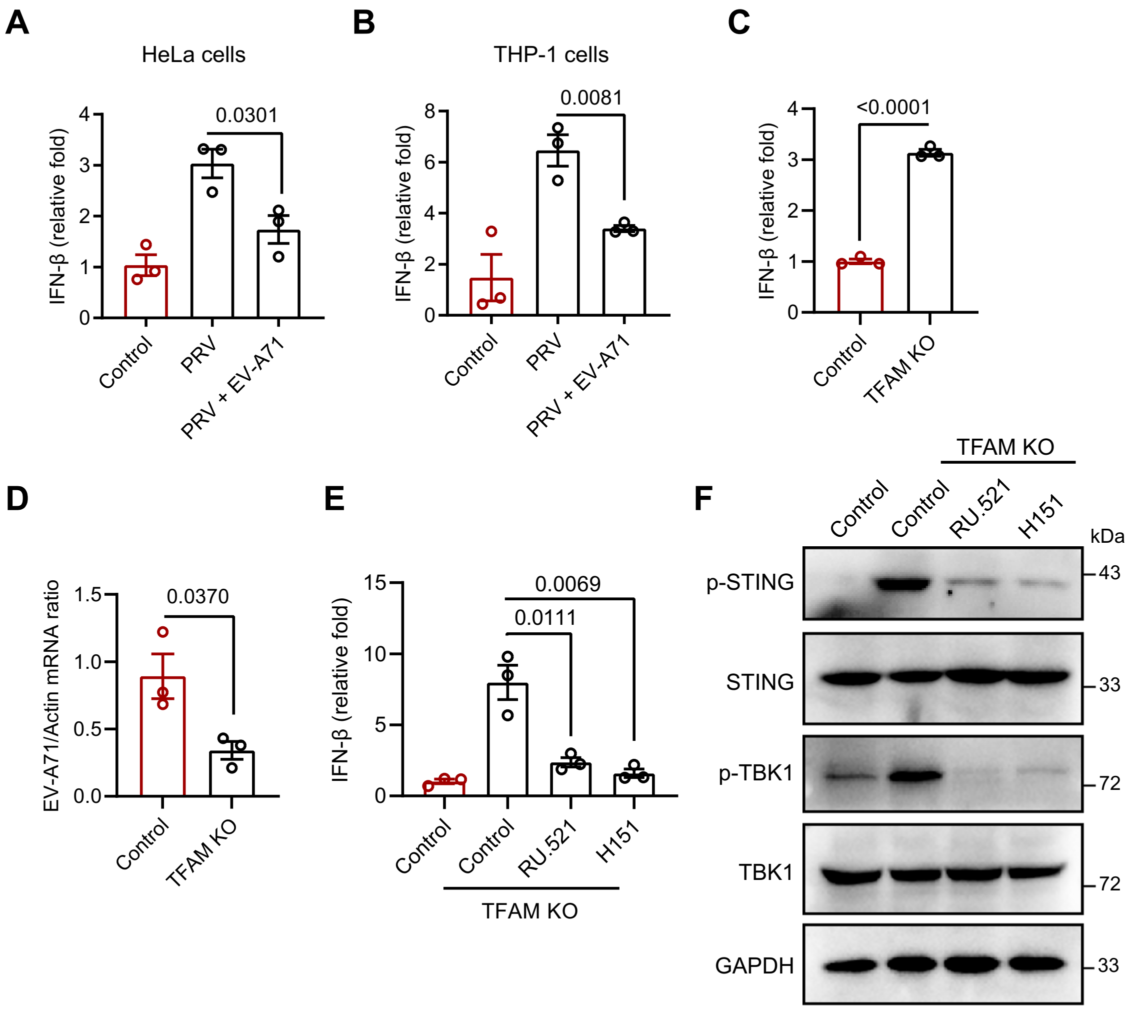

Figure EV2. TFAM knockout enhances IFN-I expression and suppresses EV-A71 infection.

(**A**, **B**) HeLa and THP-1 cells were infected with PRV for 12 h, followed by EV-A71 infection (MOI = 1) for 12 h and 24 h, respectively. qRT–PCR was then performed to measure the expression of IFN-β mRNA. Data were presented as mean ± SEM, $n = 3$ biological replicates. Unpaired *t* test was used for statistical analysis. (**C**) IFN-β mRNA expression was detected by qRT–PCR in TFAM knockout cells. Data were presented as mean ± SEM, $n = 3$ biological replicates. Unpaired *t* test was used for statistical analysis. (**D**) Intracellular viral load was assessed at 12 h post-infection using qRT–PCR in TFAM knockout cells. Data were presented as mean ± SEM, $n = 3$ biological replicates. Unpaired *t* test was used for statistical analysis. (**E**, **F**) TFAM knockout cells were treated with RU.521 (10 μM) or H151 (1 μM) for 4 h, after which IFN-β mRNA expression was measured by qRT–PCR (**E**), and protein levels of total and phosphorylated TBK1 and STING were analyzed by western blotting (**F**). Data were presented as mean ± SEM, $n = 3$ biological replicates. Unpaired *t* test was used for statistical analysis. The experiments were repeated at least three times with the similar results. Source data are available online for this figure.

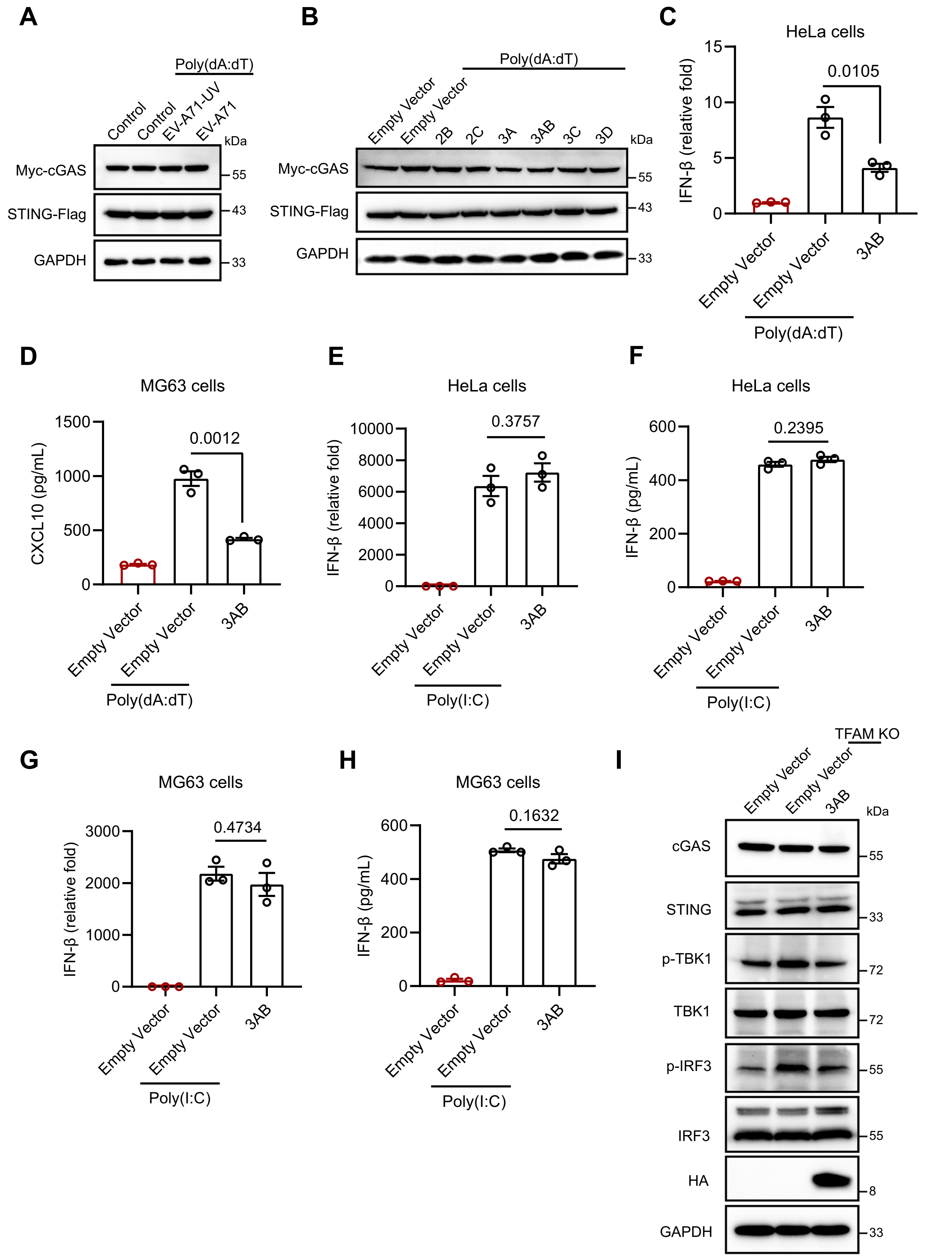
A
Poly(dA:dT)
Control
Control
EV-A71-UV
EV-A71
kDa
Myc-cGAS
55
STING-Flag
43
GAPDH
33
B
Poly(dA:dT)
Empty Vector
Empty Vector
2B
2C
3A
3AB
3C
3D
kDa
Myc-cGAS
55
STING-Flag
43
GAPDH
33
C
HeLa cells
IFN-β (relative fold)
0.0105
Empty Vector
Empty Vector
3AB
Poly(dA:dT)
D
MG63 cells
CXCL10 (pg/mL)
0.0012
Empty Vector
Empty Vector
3AB
Poly(dA:dT)
E
HeLa cells
IFN-β (relative fold)
0.3757
Empty Vector
Empty Vector
3AB
Poly(I:C)
F
HeLa cells
IFN-β (pg/mL)
0.2395
Empty Vector
Empty Vector
3AB
Poly(I:C)
G
MG63 cells
IFN-β (relative fold)
0.4734
Empty Vector
Empty Vector
3AB
Poly(I:C)
H
MG63 cells
IFN-β (pg/mL)
0.1632
Empty Vector
Empty Vector
3AB
Poly(I:C)
I
TFAM KO
Empty Vector
Empty Vector
3AB
kDa
cGAS
55
STING
33
p-TBK1
72
TBK1
72
p-IRF3
55
IRF3
55
HA
8
GAPDH
33

 © The Author(s)

Figure EV3. EV-A71 3AB inhibits DNA-mediated IFN-I responses.

(**A**) cGAS, STING, poly(dA:dT), and IFN-β luciferase reporter plasmids were transfected into 293 T cells, which were then infected with EV-A71 or UV-inactivated EV-A71 for 12 h. The overexpression of cGAS and STING was assessed by Western blotting. (**B**) cGAS, STING, poly(dA:dT), and IFN-β luciferase reporter plasmids were transfected into 293T cells, which were then transfected with EV-A71 nonstructural plasmids for 24 h. The overexpression of cGAS and STING was assessed by Western blotting. (**C**) HeLa cells were transfected with 100 ng/mL poly(dA:dT) for 6 h, followed by transfection with EV-A71 3AB plasmid for 24 h. qRT–PCR was then performed to measure the expression of IFN-β mRNA. Data were presented as mean ± SEM, $n = 3$ biological replicates. Unpaired *t* test was used for statistical analysis. (**D**) MG63 cells were transfected with 100 ng/mL poly(dA:dT) for 6 h, followed by transfection with EV-A71 3AB plasmid for 18 h. CXCL10 protein expression in supernatant was determined by ELISA. Data were presented as mean ± SEM, $n = 3$ biological replicates. Unpaired *t* test was used for statistical analysis. (**E**, **F**) HeLa cells were transfected with EV-A71 3AB plasmid for 24 h, followed by transfection with 500 ng/mL poly(I:C) for 12 h. (**E**) The mRNA expression of IFN-β was assessed by qRT–PCR. (**F**) IFN-β protein expression in supernatant was determined by ELISA. Data were presented as mean ± SEM, $n = 3$ biological replicates. Unpaired *t* test was used for statistical analysis. (**G**, **H**) MG63 cells were transfected with EV-A71 3AB plasmid for 18 h, followed by transfection with 500 ng/mL poly(I:C) for 6 h. (**G**) The mRNA expression of IFN-β was assessed by qRT–PCR. (**H**) IFN-β protein expression in supernatant was determined by ELISA. Data were presented as mean ± SEM, $n = 3$ biological replicates. Unpaired *t* test was used for statistical analysis. (**I**) After 24 h of 3AB overexpression, Western blotting analysis was performed to assess cGAS, STING levels, as well as phosphorylated (p-) and total TBK1 and IRF3 levels, in TFAM knockout and mock cells. The experiments were repeated at least three times with the similar results. Source data are available online for this figure.

© The Author(s)

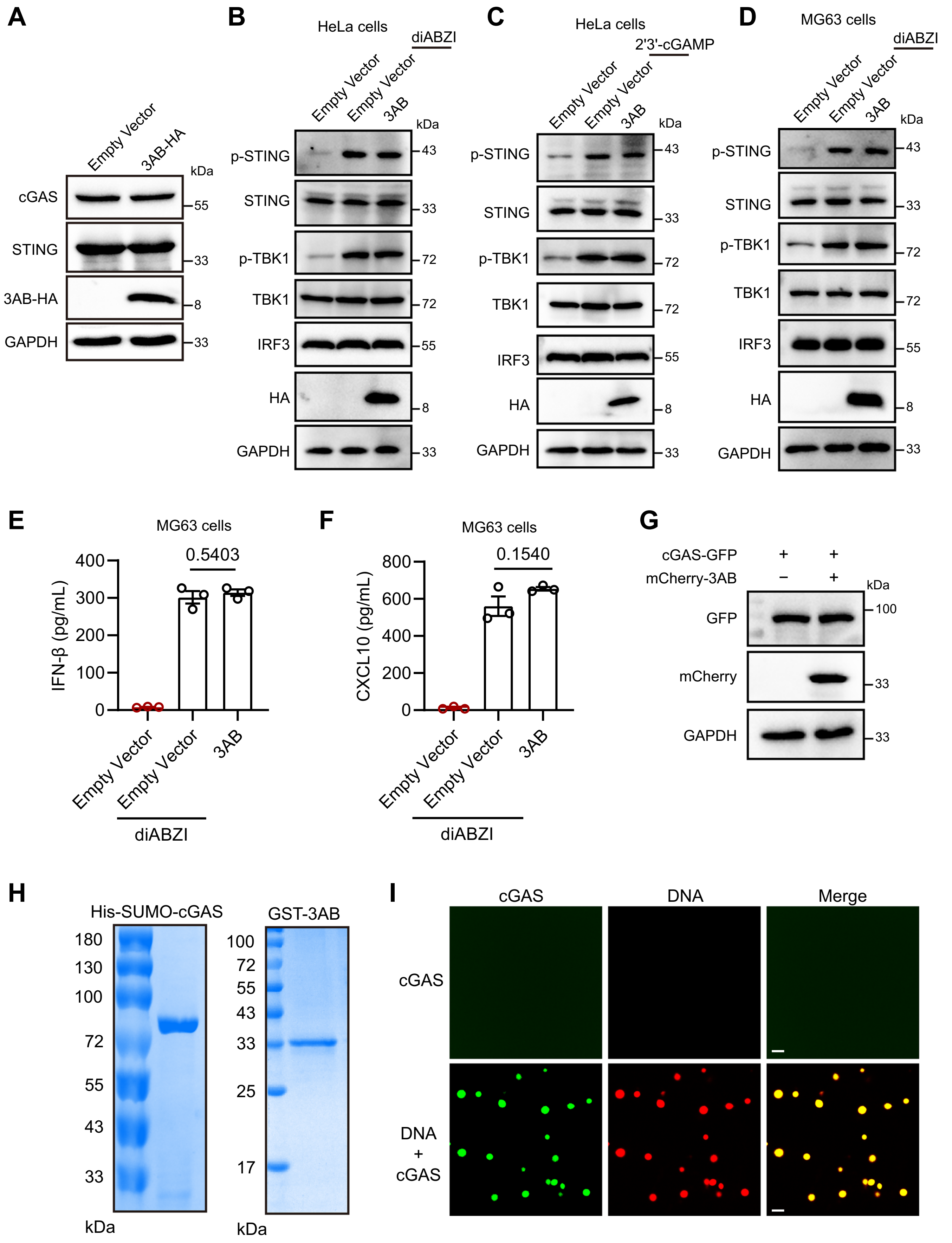
A
Empty Vector
3AB-HA
kDa
cGAS
STING
3AB-HA
GAPDH
55
33
8
33
B
HeLa cells
diABZI
Empty Vector
Empty Vector
3AB
kDa
p-STING
STING
p-TBK1
TBK1
IRF3
HA
GAPDH
43
33
72
72
55
8
33
C
HeLa cells
2'3'-cGAMP
Empty Vector
Empty Vector
3AB
kDa
p-STING
STING
p-TBK1
TBK1
IRF3
HA
GAPDH
43
33
72
72
55
8
33
D
MG63 cells
diABZI
Empty Vector
Empty Vector
3AB
kDa
p-STING
STING
p-TBK1
TBK1
IRF3
HA
GAPDH
43
33
72
72
55
8
33
E
MG63 cells
0.5403
IFN-β (pg/mL)
400
300
200
100
0
Empty Vector
Empty Vector
3AB
diABZI
F
MG63 cells
0.1540
CXCL10 (pg/mL)
800
600
400
200
0
Empty Vector
Empty Vector
3AB
diABZI
G
cGAS-GFP + +
mCherry-3AB − +
kDa
GFP
mCherry
GAPDH
100
33
33
H
His-SUMO-cGAS
180
130
100
72
55
43
33
kDa
GST-3AB
100
72
55
43
33
25
17
kDa
I
cGAS
DNA
Merge
cGAS
DNA
+
cGAS

Figure EV4. EV-A71 3AB does not inhibit STING agonist–induced innate immune responses.

(**A**) After 24 h of 3AB overexpression, Western blotting analysis was performed to assess total cGAS, STING levels in HeLa cells. (**B**) The EV-A71 3AB plasmid was transfected into HeLa cells for 24 h, followed by treatment with diABZI (5 μM) for 4 h. Protein levels of IRF3, as well as total and phosphorylated TBK1 and STING, were analyzed by Western blotting. (**C**) HeLa cells were transfected with EV-A71 3AB plasmid for 24 h, followed by transfection with 2'3'-cGAMP (5 μg/mL) for 6 h. Protein levels of IRF3, as well as total and phosphorylated TBK1 and STING were analyzed by Western blotting. (**D**) The EV-A71 3AB plasmid was transfected into MG63 cells for 18 h, followed by treatment with diABZI (5 μM) for 4 h. Protein levels of IRF3, as well as total and phosphorylated TBK1 and STING, were analyzed by western blotting. (**E**, **F**) MG63 cells were transfected with the EV-A71 3AB plasmid for 18 h, followed by treatment with diABZI (5 μM) for 4 h. The protein levels of IFN-β (**D**) and CXCL10 (**E**) in supernatant were determined by ELISA. Data were presented as mean ± SEM, $n = 3$ biological replicates. Unpaired *t* test was used for statistical analysis. (**G**) HeLa cells were co-transfected with cGAS-GFP, mCherry-3AB plasmids and mtDNA for 24 h. Western blotting analysis was performed to assess cGAS and 3AB using anti-GFP and anti-mCherry antibody. (**H**) Purification of the recombinant His-SUMO-cGAS and GST-3AB proteins produced in the *Escherichia coli* BL21 DE3 strain. The proteins were detected by Coomassie blue staining in SDS–PAGE gels. (**I**) Representative images of phase separation by mixing cGAS (20 μM) with 100-bp dsDNA (10 μM) in buffer containing 20 mM Tris-HCl (pH 7.5), 150 mM NaCl, 10 mM MgCl2 and 1 mg/mL BSA. Images were examined with a Leica STELLARIS 5 confocal microscope. Scale bar, 5 μm. The experiments were repeated at least three times with the similar results. Source data are available online for this figure.

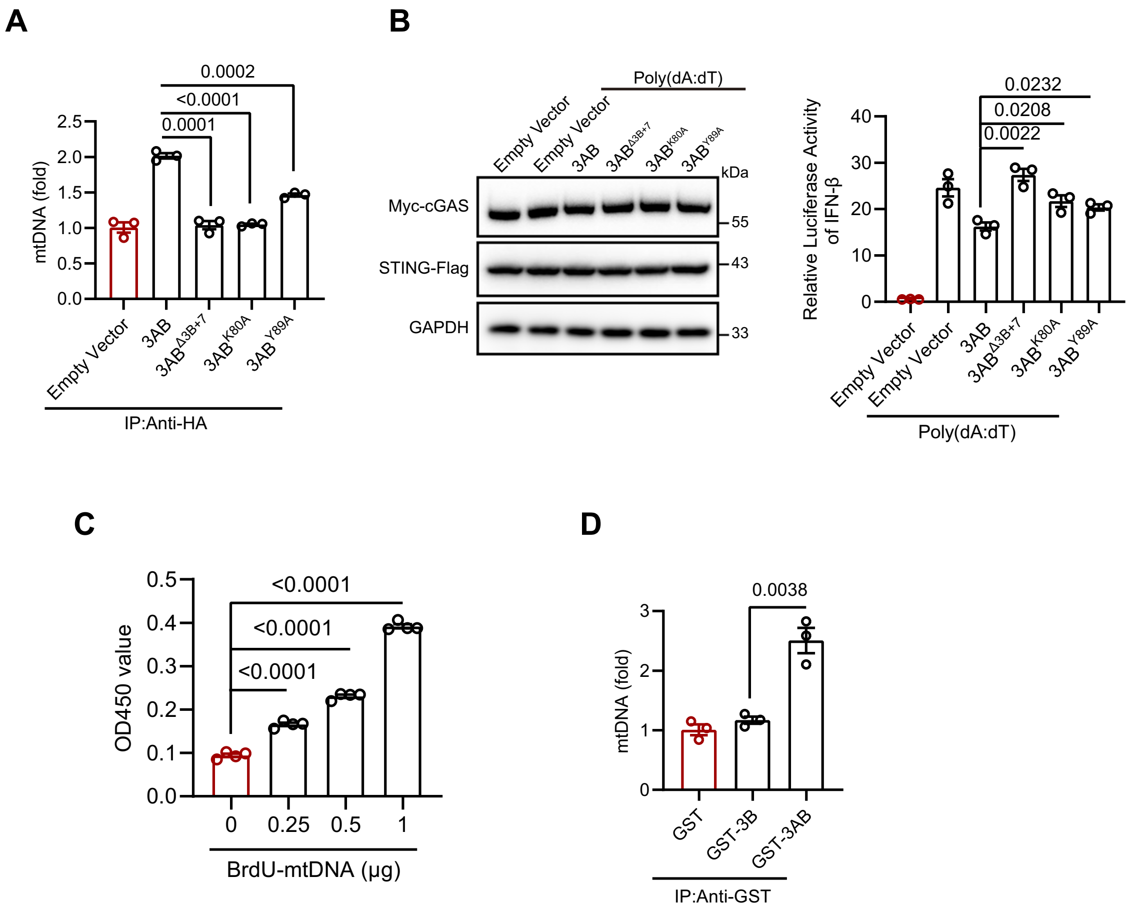

Figure EV5. EV-A71 3AB protein directly interacts with cytosolic mtDNA.

(**A**) After 24 h of co-transfection with 3AB mutants plasmids and mtDNA, the cytosol was isolated, followed by immunoprecipitation with anti-HA. qPCR was performed to measure the cytosolic mtDNA levels. Data were presented as mean ± SEM, $n = 3$ biological replicates. Unpaired *t* test was used for statistical analysis. (**B**) cGAS, STING, poly(dA:dT), and IFN-β luciferase reporter plasmids were transfected into 293 T cells, which were then transfected with EV-A71 mutants plasmids for 24 h. The overexpression of cGAS and STING was assessed by Western blotting, and IFN-β luciferase activity was measured with a dual-luciferase reporter assay. Data were presented as mean ± SEM, $n = 3$ biological replicates. Unpaired *t* test was used for statistical analysis. (**C**) Purified GST-3AB protein was coated onto a 96-well ELISA plate and incubated overnight at 4 °C. BrdU-labeled mtDNA was then added and incubated for 2 h at room temperature. After washing, the bound mtDNA was detected using anti-BrdU antibodies. Data were presented as mean ± SEM, $n = 4$ biological replicates. Unpaired *t* test was used for statistical analysis. (**D**) Purified GST-3AB protein and mtDNA were incubated together in binding buffer. The protein–mtDNA complexes were immunoprecipitated using anti-GST antibodies, and the associated mtDNA was quantified by qPCR. Data were presented as mean ± SEM, $n = 3$ biological replicates. Unpaired *t* test was used for statistical analysis. The experiments were repeated at least three times with the similar results. Source data are available online for this figure.

 © The Author(s)
